# Tangled history of a multigene family: The evolution of *ISOPENTENYLTRANSFERASE* genes

**DOI:** 10.1371/journal.pone.0201198

**Published:** 2018-08-02

**Authors:** Kanae Nishii, Frank Wright, Yun-Yu Chen, Michael Möller

**Affiliations:** 1 Royal Botanic Garden Edinburgh, Scotland, United Kingdom; 2 Tokyo Gakugei University, Koganei, Tokyo, Japan; 3 Biomathematics and Statistics Scotland, Edinburgh, Scotland, United Kingdom; 4 University of Edinburgh, Edinburgh, United Kingdom; USDA-ARS, UNITED STATES

## Abstract

*ISOPENTENYLTRANSFERASE* (*IPT*) genes play important roles in the initial steps of cytokinin synthesis, exist in plant and pathogenic bacteria, and form a multigene family in plants. Protein domain searches revealed that bacteria and plant IPT proteins were to assigned to different protein domains families in the Pfam database, namely Pfam IPT (IPT^Pfam^) and Pfam IPPT (IPPT^Pfam^) families, both are closely related in the P-loop NTPase clan. To understand the origin and evolution of the genes, a species matrix was assembled across the tree of life and intensively in plant lineages. The IPT^Pfam^ domain was only found in few bacteria lineages, whereas IPPT^Pfam^ is common except in Archaea and *Mycoplasma* bacteria. The bacterial IPPT^Pfam^ domain *miaA* genes were shown as ancestral of eukaryotic IPPT^Pfam^ domain genes. Plant *IPT*s diversified into class I, class II tRNA-*IPT*s, and Adenosine-phosphate *IPT*s; the class I tRNA-*IPT*s appeared to represent direct successors of *miaA* genes were found in all plant genomes, whereas class II tRNA-*IPT*s originated from eukaryotic genes, and were found in prasinophyte algae and in euphyllophytes. Adenosine-phosphate *IPT*s were only found in angiosperms. Gene duplications resulted in gene redundancies with ubiquitous expression or diversification in expression. In conclusion, it is shown that *IPT* genes have a complex history prior to the protein family split, and might have experienced losses or HGTs, and gene duplications that are to be likely correlated with the rise in morphological complexity involved in fine tuning cytokinin production.

## Introduction

The evolution of gene families can be complex and may involve duplications within genomes or through polyploidization and loss or conversion events, these being the major forces enlarging gene families, with mutations accumulating over time further differentiating individual family members [1, 2]. ISOPENTENYLTRANSFERASE (IPT) enzymes regulate a rate limiting step in the biosynthesis pathway of cytokinin, an important hormone [3]. They also have other functions such as stabilizing codon recognition of tRNA through the modification of tRNA in yeast. In mammals they are linked to mitochondrial diseases [4, 5]. Cytokinins are not only found in plants, but also in plant pathogenic bacteria such as the crown-gall forming *Agrobacterium tumefaciens* (reviewed in [6]), the cyanobacterium *Nostoc* sp. PCC7120 [7], and the slime-mold *Dictyostelium discoideum* [8].

*IPT* genes were first identified in *A*. *tumefaciens* [9, 10] and only much later in *Arabidopsis thaliana* [11, 12], after the release of its genome sequence [13], and nine *IPT* genes were identified in the genome [14]. To date, *IPT*s have been studied in several angiosperms and mosses (e.g., *Arabidopsis thaliana* [14]; *Oryza sativa* [15]; *Physcomitrella patens* [16]; *Solanum lycopersicum* [17]), and were shown to belong to one multigene family [14, 15, 18, 19, 20, 21]. In *A*. *thaliana*, they are classified into two types depending on the substrates they use; Adenosine-phosphate *IPT*s (AP-*IPT*s) and tRNA-*IPT*s [14]. *Agrobacterium tumefaciens* also retaining AP-*IPT*, preferentially uses AMP whereas those in plants prefer ATP and ADP as substrates [22].

In previous studies, Frébort et al. [18] classified *IPT* genes into five groups: `bacterial adenylate *IPT*s`, `plant adenylate *IPT*s`, `eukaryotic origin plant tRNA *IPT*s`, `bacterial tRNA *IPT*s`, and `prokaryotic origin plant tRNA *IPT*s`, based on an unrooted gene tree reconstructed from full sequence lengths, where members of two plant families (*A*. *thaliana*; *O*. *sativa*) were included. Lindner et al. [19] carried out a more comprehensive analysis with 30 species across kingdoms including 12 plant families, in which they separated plant *IPT*s into `class I tRNA-*IPT*s`, `class II tRNA-*IPTs*`and `adenylate-*IPT*s`, and bacteria *IPT*s into `bacterial tRNA-*IPT*s`and `bacterial AMP-*IPT*s`, using a midpoint rooted Bayesian inference tree. The cytokinin synthesizing genes of the bacteria *A*. *tumefaciens* and the slime-mold *D*. *discoideum* were found to belong to the AMP-*IPT* clade and were separated from plant *IPT* clades in Lindner et al. [19]. The authors further showed that class I tRNA-*IPT*s are closely related to bacteria tRNA-*IPT*s, and class II tRNA-*IPT*s to adenylate-*IPT*s [19].

The two different classifications by Frébort et al. [18] and Lindner et al. [19] are not fully congruent, principally because they did not included the same groups of organisms (Table 1). Furthermore, the evolutionary history of *IPT*s was not fully explained in the two studies since the phylogenetic trees were unrooted, and the direction of evolution as well as the origin of the gene family unexplored. A further complication might have been that the full sequence and protein sequences between the different groups of *IPT*s are highly divergent and their alignment might have included ambiguous alignment information, obscuring the phylogenetic signal [23].

**Table 1:** Classification of *ISOPENTENYLTRANSFERASE* genes.

Gene classification in Frébort et al. [18]	Gene classification in Lindner et al. [19]	Gene classification in this study	Clade/Grade	Domain	Lineages found in clade
Bacterial adenylate *IPT*s	AMT-*IPT*	Outgroup		IPT^Pfam^	Bacteria; Slime-mold
Bacterial tRNA *IPT*s	-	Bacteria *miaA* grade	A	IPPT^Pfam^	Bacteria
Prokaryotic origin plant tRNA *IPT*s	Class I tRNA-*IPT*	Plant class I tRNA-*IPT*	B	IPPT^Pfam^	Algae; Mosses; Lycophytes; Gymnosperms; Angiosperms
-	-	Unikont-SAR tRNA-*IPT* grade	C	IPPT^Pfam^	Mammals; Insect; Fungi; Slime-mold; Zooplankton
-	Class II tRNA-*IPT*	Prasinophyte tRNA-*IPT*	D	IPPT^Pfam^	Prasinophyte algae
Eukaryotic origin plant tRNA *IPT*s	Class II tRNA-*IPT*	Plant class II tRNA-*IPT*	E	IPPT^Pfam^	Gymnosperms; Angiosperms
Plant adenylate *IPT*s	ADP/ATP-*IPT*s	Adenosine-phosphate *IPT*	F	IPPT^Pfam^	Angiosperms

IPT^Pfam^, IPPT^Pfam^–referring to Pfam protein families IPT and IPPT.

Therefore, this study focused on the conserved protein domain of the *IPT*s, to infer the deep origin and evolution of this gene family. The conserved protein domain of *IPT* genes across kingdoms were assembled with a focus on plants and the matrix included 37 plants (of 21 families), three animals, two fungi, one amoeba, and one zooplankton species, selected across the evolutionary breadth of the tree of life [24, 25, 26]. The results of these domain based phylogenetic analyses are discussed in the light of the frequency and timing of duplication events, and linked to expression patterns of the gene copies and their intron positions as reported in previous studies. This is the first detailed analysis to illustrate the origin and pattern of diversification of *IPT* genes in plants in a phylogenetic context.

## Materials and methods

### Genome resources

*IPT* genes were retrieved from publicly accessible genome or transcriptome databases. The list of species analysed and the databases used in this study are listed in S1 Table. The gene accession numbers are listed in S2 Table.

### Domain searches

Domain searches were carried out using deduced amino acid sequences in Pfam v.31.0 [27]. Since *IPT*s are mostly single-domain proteins and retain either IPT^Pfam^ (Pfam family IPT) or IPPT^Pfam^ (Pfam family IPPT) domains, these were searched across kingdoms, including Archaea, bacteria, plants, yeast, animals, and slime-molds (S2 Table). Proteins possessing the IPT^Pfam^ domain are described as isopentenyl transferases or dimethylallyl transferases and synthesise cytokinin, while those possessing the IPPT^Pfam^ domain are IPP transferases/tRNA delta(2)-isopentenylpyrophosphate transferases and modify tRNA to stabilize codon recognition in a wide range of lineages (e.g., bacteria, fungi, mammals). They use AMP/ADP/ATP as substrates and contribute to cytokinin synthesis in plants [3]. The genome and transcriptome databases were BLAST searched (cut-off *E* < 0.1) using IPPT^Pfam^ and IPT^Pfam^ domains from *A*. *thaliana* and *A*. *tumefaciens*. Sequence matches were re-evaluated in Pfam searches, and only gene sequences clearly showing IPPT^Pfam^ and IPT^Pfam^ domain sequences were used for this study (S2 Table).

### Assessing relationships among domain families

The protein families in the clan P-loop NTPase (CL0023), including IPT^Pfam^ (PF01745) and IPPT^Pfam^ (PF01715) protein domain families, were analysed. This clan included 217 protein domain families in Pfam v.31.0, and their Hidden Markov Model (HMM) profiles were downloaded from the Pfam website. HMM profiles estimate the true frequency of protein residues from the observed frequency by a Markov process with hidden status [28]. The HMM profile relationships were analysed and a distance matrix of HMM profiles and its unrooted Neighbor Joining tree generated using pHMM-tree [29].

Following the topology of the pHMM-tree, IPT^Pfam^ (PF01715) and IPPT^Pfam^ (PF01745) domain sequences were analysed using VirE^Pfam^ (PF05272) domain sequences as outgroup to focus on the phylogenetic relationship between IPT^Pfam^ and IPPT^Pfam^. Sequences in the seed alignments of the three families were combined into a matrix. The seed alignment of IPT^Pfam^ contains seven, and that of VirE^Pfam^ six sequences and all were used in the analyses. The IPPT^Pfam^ seed alignment is large and contains 1247 sequences, and only representative sequences were selected for the analyses: to select sequences, preliminary phylogenetic analyses were carried out on the IPPT^Pfam^ seed alignment using all sequences. Hypervariable regions of the original IPPT^Pfam^ seed alignment were trimmed with BMGE v.1.12 [30], and a phylogenetic tree reconstructed with FastTree [31], and 162 topology-representative sequences selected. Finally, the reduced IPPT^Pfam^ seed alignment (162 sequences), IPT^Pfam^ seed alignment (7 sequences), and VirE^Pfam^ seed alignment (6 sequences), were combined with the MAFFT-merge subprogram in MAFFT v.7 [32], and the matrix was trimmed with BMGE v.1.12 [30]. An ML tree estimated with PhyML v.3.0 [33] with Smart Model Selection (SMS) [34] with the tree rooted on VirE^Pfam^ sequences. For branch support, values of an approximate likelihood ratio test with non-parametric branch support based on a Shimodaira-Hasegawa-like procedure (αLRT SH-like support) were estimated using PhyML. Additionally, an ultrafast bootstrap (UFBT) analysis of 1,000 replicates was carried out in W-IQ-TREE [35].

### Building IPPT^Pfam^ HMM alignments with extended N-terminus region

**Fig 1:**
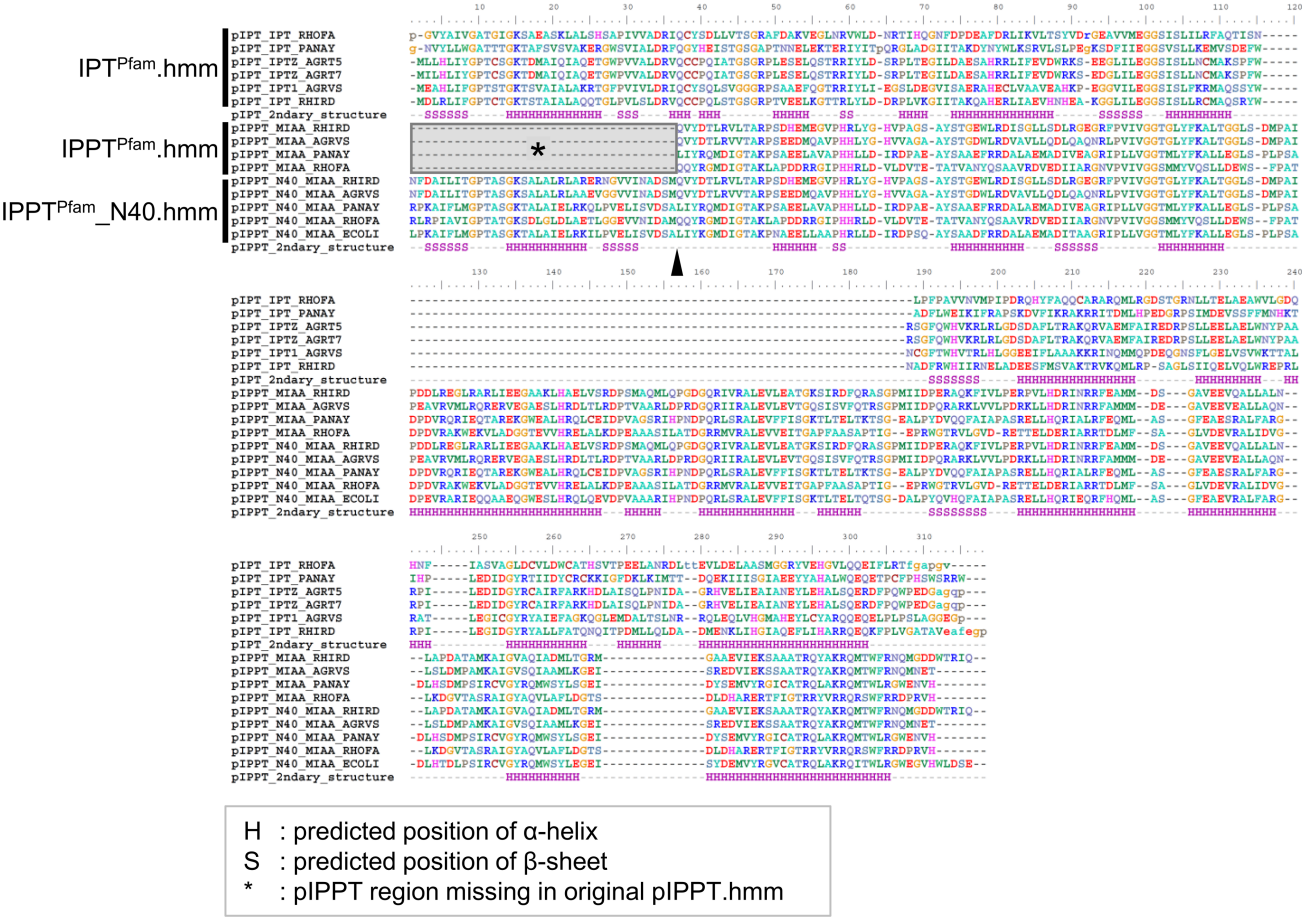
Domain sequences used for HMM profiles. Original IPPT^Pfam^ domain sequences were shorter than the IPT^Pfam^ domain by approximately 40 AA at the N-terminus. The expanded profile was retrieved from full sequences and used for the new HMM profile (IPPT^Pfam^_N40.hmm). Arrowhead indicates the starting position of the original IPPT^Pfam^.hmm. Box marked with an asterisk indicate the IPPT^Pfam^ region missing in the original IPPT^Pfam^.hmm. Predicted positions of α-helixes are indicated by ‘H’, and those of β-sheet by ‘S’.

The IPPT^Pfam^ original seed alignment with 1247 sequences was reduced to 103 representative sequences as described above. To confirm the similarity between the original (1247 sequences) and the representative sequences, HMM profiles were built for the 1247 and 103 sequences respectively, with hmmerbuild in HMMER v.3.0 [28], and HMM logos were generated with Skylign [36] and the logos compared. After confirming their similarity, full-lengths of the 103 representative sequences were retrieved from the database and the N-terminus region aligned manually. 101 out of the 103 sequences were found to have retained the approximately 40 AA long conserved region located in front of the starting point of the original IPPT^Pfam^ HMM (Fig 1). A new HMM profile was built that included those 40 AA sequences with hmmerbuild, its HMM logo generated, and the profile named IPPT^Pfam^_N40.hmm. To annotate and check the protein alignment, the protein structures of IPT^Pfam^ and IPPT^Pfam^ domains were retrieved from the PDBsum-EMBL-EBI database (http://www.ebi.ac.uk/pdbsum), for IPT^Pfam^ from *Agrobacterium tumefaciens* (PDBsum accession number: 2ze5) and for IPPT^Pfam^ from *Escherichia coli* (3foz) as references.

### Assessing plant IPT^Pfam^ domain in Pfam database

Fragmental IPT^Pfam^ domains were found in species in a few plant families in the Pfam database (e.g., *Musa acuminata*, *Solanum lycopersicum*). Those plant IPT^Pfam^ domain genes registered in Pfam were retrieved and assessed with hmmersearch in HMMER v.3.0, which compares the protein sequences with IPPT^Pfam^.hmm and IPT^Pfam^.hmm from Pfam, and IPPT^Pfam^_N40.hmm built in this study, to examine the similarities between the domain sequences and the HMM profiles.

In addition, a phylogenetic analysis was carried out with plant genes registered under IPT^Pfam^ domains in the database. The matrix was assembled with plant IPT^Pfam^ domain genes together with the bacterial IPT^Pfam^ domain genes, the bacterial IPPT^Pfam^ genes (*miaA*), and IPPT^Pfam^ genes from *P*. *patens*, *A*. *thaliana*, *O*. *sativa*, *S*. *lycopersicum*, *S*. *tuberosum*, *M*. *acuminata*. The IPPT^Pfam^ and IPT^Pfam^ domain sequences were first aligned separately using the hmmeralign in the HMMER v.3.0 with IPT^Pfam^.hmm or IPPT^Pfam^_N40.hmm. The two alignments were merged using MAFFT merge v.7 [32] and trimmed using BMGE v.1.12 [30]. The WAG model was selected under the AIC criterion [37] using Prottest v.3.0 [38], and an ML tree and αLRT SH-like support values were estimated with PhyML v.3.0 [33].

### Detecting the presence of IPT^Pfam^ domain genes in bacteria and slime-mold and their phylogenetic relationship to IPPT^Pfam^ domain genes

To show the presence or absence of IPPT^Pfam^ and IPT^Pfam^ domain genes in bacteria and slime-mold, a species tree based on Battistuzzi et al. [39] and Tomitani et al. [40] was generated and annotated with the presence and absence of the domain genes. Yeast was added as outgroup. A Newick file was generated manually in a text editor and the tree modified in TreeView v.1.6.6 [41] and FigTree v.1.4.2 (http://tree.bio.ed.ac.uk/software/figtree/).

The phylogenetic tree of IPPT^Pfam^ and IPT^Pfam^ domain sequences of bacteria, slime-mold and yeast was build alongside the species tree generated above. IPPT^Pfam^ and IPT^Pfam^ domain sequences were aligned separately using hmmeralign with IPPT^Pfam^_N40.hmm or IPT^Pfam^.hmm. The two alignments were merged using MAFFT merge v.7 and trimmed using Gblocks [42]. The LG model was selected under the AIC criterion using Prottest v.3.0, and an ML tree estimated with PhyML v.3.0. αLRT SH-like support values were estimated using PhyML and an UFBT analysis of 10,000 replicates carried out in W-IQ-TREE [35].

### Comprehensive phylogeny of IPPT^Pfam^ domain genes across kingdoms

To build the comprehensive phylogenetic IPPT^Pfam^ domain gene tree, IPPT^Pfam^ domain genes were retrieved from genome databases from algae to angiosperms. bacteria, animals, yeast, slime-molds, and zooplankton genes were also included in the analyses (S2 Table). The matrix was generated as described above for the bacterial IPPT^Pfam^ and IPT^Pfam^ phylogeny and trimmed using BMGE v.1.12. The LG model was selected under the AIC criterion using Prottest v.3.0, and an IPT^Pfam^ rooted ML tree with branch support value estimated as above with PhyML and W-IQ-TREE.

### Estimating the timing of duplication of *ISOPENTENYLTRANSFERASE* in plants

To estimate the number and timings of duplications of *IPT*s specifically among plants, gene duplication and losses (DL) analyses were carried. Gene subtrees containing plant *IPT*s were reconciled and rearranged with plant species trees separately for class I tRNA *IPT*s and class II tRNA-*IPT/*AP-*IPT*s (S9–S14 Figs) in DL mode, considering duplications and losses, with NOTUNG v.2.9 using default settings [43]. To allow the topological support existing within the *IPT* clades to optimize the duplication-loss events, the αLRT SH-like branch support values of the ML analysis were transferred to the gene subtree in the NOTUNG analyses.

To place the history of *IPT*s in a phylogenetic timeframe, divergence times for major lineages and species were referred from key published analyses (angiosperms-liverworts [44], charophytes-red algae [45], eukaryotic lineages [46], prokaryotic lineages [39]), and a metric summary tree of life phylogeny constructed and the transfer and duplication event placed in that tree.

### Intron distribution

Intron-exon structures were also examined, by interrogating databases and comparing genome and transcribed sequences. The number of nucleotides in exons and introns were determined and schematic illustrations based on their number, size and position drawn with GSDS v.2.0 [47].

### Diversification of expression patterns

Literature searches were carried out to obtain an overview of gene expression patterns in relation to the duplication history of *IPT* genes. For interspecific comparisons, the expression data were categorised into root, leaf, flower, and fruit. For mosses, the protonema, mature gametophytic stage, and sporophytic stage were reported and these categories were used here. The literature used in this study regarding gene expression patterns are summarized in S3 and S4 Tables.

### Accession numbers

The accession numbers of the sequences used in this study are listed in S2 and S5 Tables.

### Datasets used in this study

**Fig 2:**
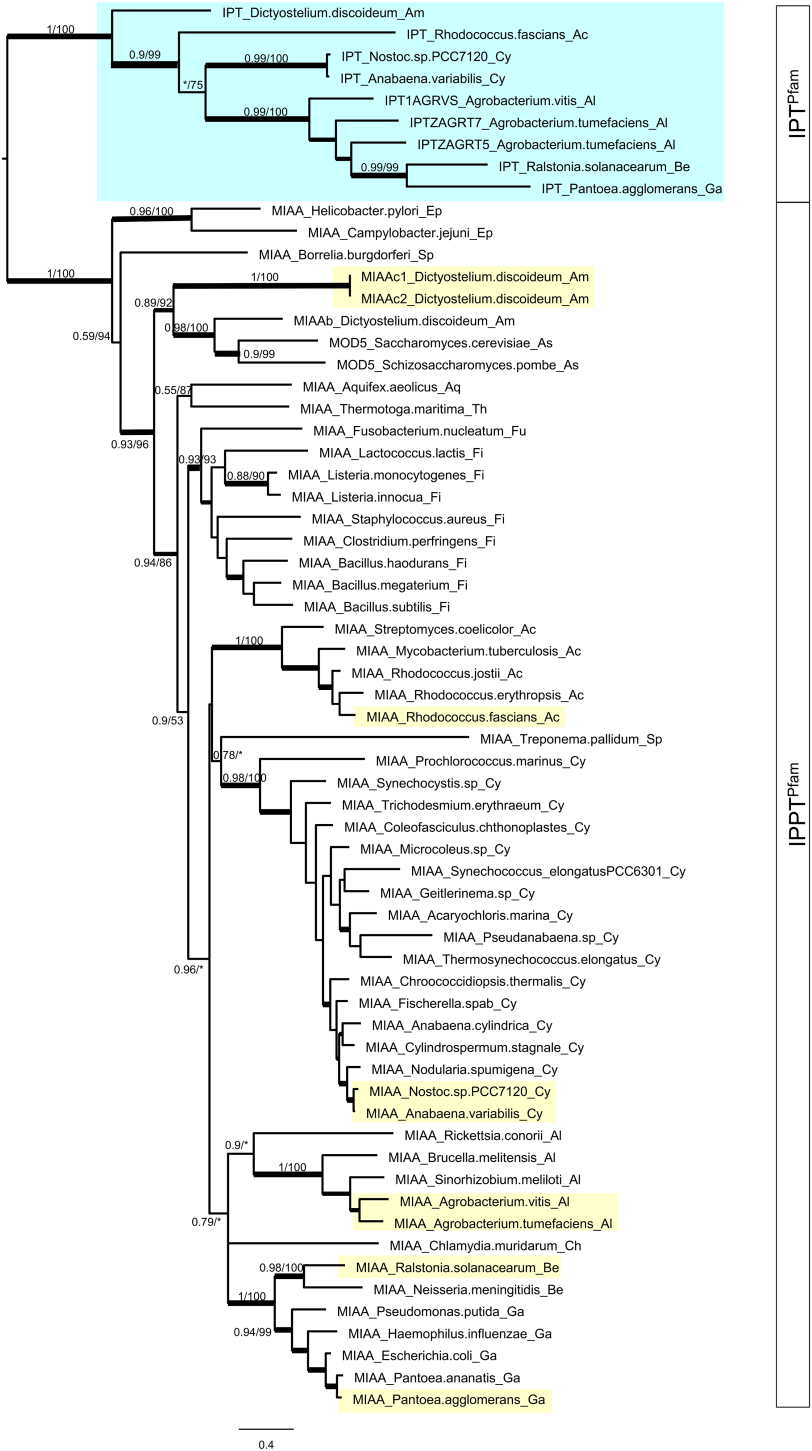
ML tree of the bacterial IPT^Pfam^ and IPPT^Pfam^ domain genes. The αLRT (left) and UFBT values (right) are shown along major branches. An asterisk indicates support values < 0.5 and < 50%. Thickened branches indicate support values > 0.9 and > 90%, medium-thick branches indicate > 0.7 and > 70%. A tree with all support values is shown in S7 Fig. The species with both IPT^Pfam^ and IPPT^Pfam^ domain genes are highlighted blue and yellow. The classification of the species is indicated by two characters at the end of the gene names; Ac: Actinobacteria, Al: α-Proteobacteria, Am: Amoebozoa, Aq: Aquficae, As: Ascomycota, Be: β-Proteobacteria, Ch: Chlamydiae, Cy: Cyanobacteria, Ep: ε-Proteobacteria, Fi: Firmicutes, Fu: Fusobacteria, Ga: γ-Proteobacteria, Sp: Spirochaetes, Th: Thermotogae.

**Fig 3:**
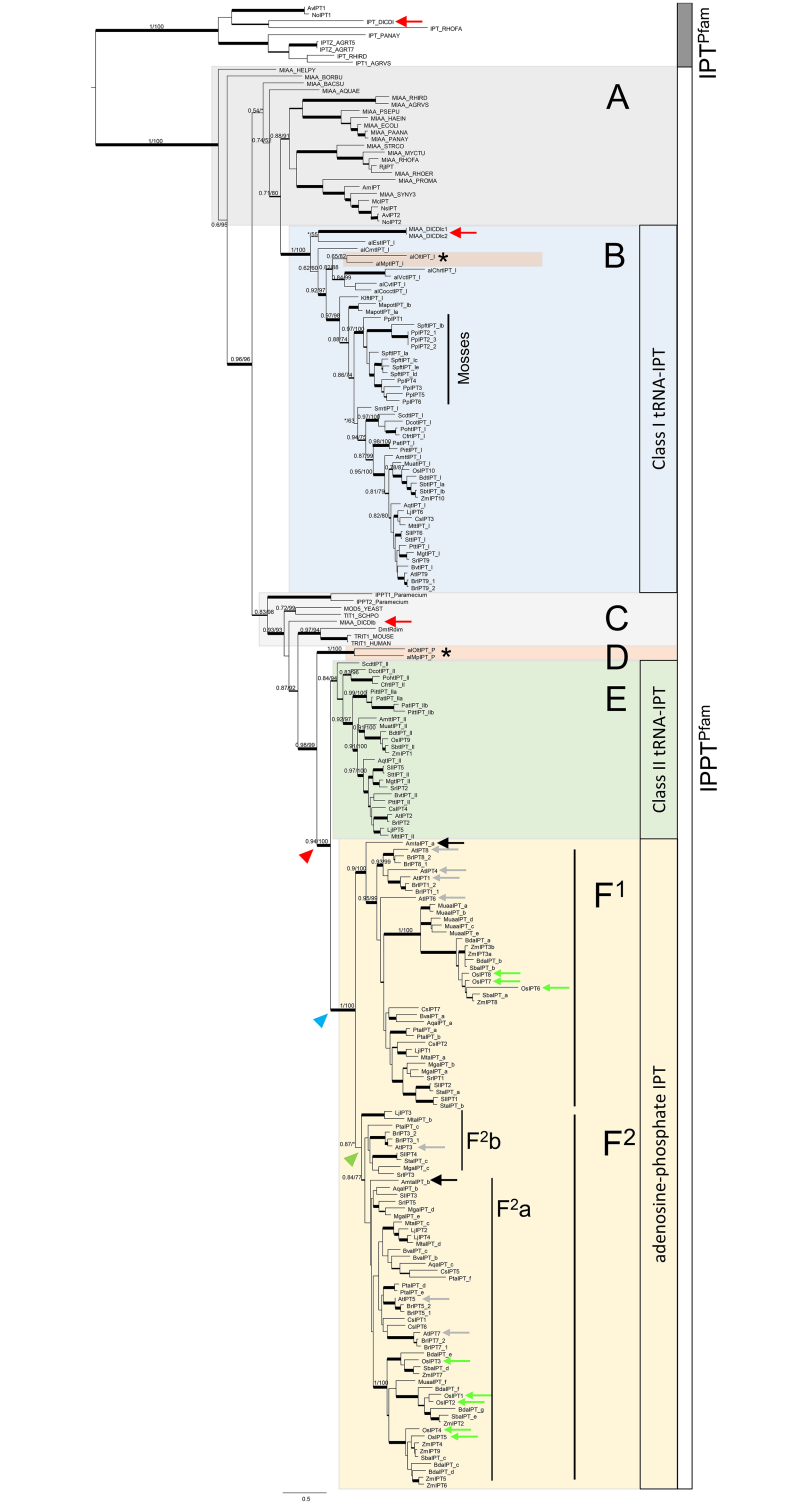
ML tree of IPPT^Pfam^ domain genes across kingdoms. IPT^Pfam^ domain genes were used as outgroup. The αLRT (left) and UFBT support values (right), are shown along the major branches. An asterisk indicates support values < 0.5 and < 50%. Thickened branches indicate support values > 0.9 and > 90%, medium-thick branches indicate > 0.7 and > 70%. Trees with all support values are shown in S8 Fig. **A**. Bacterial IPPT^Pfam^ genes, *miaA*. **B**. Plant class I tRNA-*IPT*s. Two IPPT^Pfam^ domain genes from *Dictyostelium discoideum* are nested in this clade (red arrow). The Mosses IPPT^Pfam^ clade included multiple copies of tRNA-*IPT*s from S*phagnum fallax* and *Physcomitrella patens*. **C**. Unikont-SAR *IPT*s. IPPT^Pfam^ domain genes of zooplankton, yeast, animals arranged in grades. One copy of the IPPT^Pfam^ gene of *D*. *discoideum* appeared as sister grade to the animal clade (red arrow). **D**. Prasinophyte algae tRNA-*IPT*s. Prasinophyte clade indicated by asterisk and pink box in B and D. **E**. Plant class II tRNA-*IPT*s. **F**. AP-*IPT*s. Two clades (**F**^**1**^, **F**^**2**^) were observed and the basal angiosperm *Amborella trichopoda* retained two copies, one belonging to each clade (black arrows). Derived angiosperms retained diverged copies within F^2^ (F^2^a,b). The multiple copies of *Arabidopsis thaliana* (grey arrows) and *Oryza sativa* (green arrows) are indicated. Arrowheads indicate gene duplication events inferred from NOTUNG analyses (see also Fig 5). Red arrowhead indicates gene duplication event prior to class II tRNA-*IPT* and AP-*IPT* splits, and blue and green arrowhead indicates events within plant AP-*IPT*s.

The matrixes and tree files used in this study are deposited in TreeBASE (study accession http://purl.org/phylo/treebase/phylows/study/TB2:S22409). The files include a FastTree inferred approximately-ML tree of the IPPT^Pfam^ domain seed alignment with 1247 sequences (M46567), a IPPT^Pfam^_N40.hmm new seed alignment with 103 sequences (M46568), the IPPT^Pfam^/IPT^Pfam^/VirE^Pfam^ merged matrix (with 175 sequences) and tree shown in S3 Fig (M46562, Tr112785), a plant IPT^Pfam^ domain matrix (with 101 sequences) and tree file shown in S6 Fig (M46563, Tr112786), the bacterial IPT^Pfam^/IPPT^Pfam^ domain matrix (with 64 sequences) and tree shown in Fig 2 and S7 Fig (M46565, Tr112787), and a IPT^Pfam^/IPPT^Pfam^ domain matrix (with 215 sequences) and tree across kingdoms shown in Fig 3, S8 Fig (M46566, Tr112788).

## Results

### Protein domains of cytokinin biosynthesis genes

Both *A*. *thaliana* and *A*. *tumefaciens IPT*s (*AtIPT*s for *A*. *thaliana*, *Tzs* and *Ipt* for *A*. *tumefaciens*) are single domain proteins of about 250–460 amino acid (AA) length (S1 Fig). We found that the cytokinin biosynthesis *IPT* genes in *A*. *thaliana*, *AtIPT*, and *Oryza sativa*, *OsIPT*s, possess an IPPT^Pfam^ domain, while these genes in *A*. *tumefaciens*, *Ipt* and *Tzs*, and *Nostoc* sp. PCC7120 *NoIPT1*, have an IPT^Pfam^ domain (S6 Table). Thus, cytokinin biosynthesis *IPT*s in plants and bacteria retain different domains.

IPPT^Pfam^ has a 228 AA long Hidden Markov Model (HMM) profile, and AP-*IPT* in *A*. *thaliana* has a truncated IPPT^Pfam^ domain lacking ca. 75–140 AA of the IPPT^Pfam^ HMM profile, while those of tRNA-*IPT* have almost the full length of the IPPT^Pfam^ HMM profile (S1 Fig). IPT^Pfam^ has a 233 AA HMM profile and *Tzs* or *Ipt* of *A*. *tumefaciens* possess almost the entire region of the IPT^Pfam^ HMM profile. Both, the IPPT^Pfam^ and IPT^Pfam^ domain, belong to the P-loop NTPase clan (CL0023) in the Pfam database v.31.0 [27]. This clan contains 217 families and these often perform chaperone-like functions [48, 49]. The pHMM-tree analyses of the P-loop NTPase clan suggested that IPPT^Pfam^ and IPT^Pfam^ HMM profiles are closely related and appear as sisters in the Neighbor Joining tree (S2 Fig).

When adding VirE^Pfam^ sequences as outgroups to IPPT^Pfam^ and IPT^Pfam^ domain sequences, the ML phylogenetic analyses performed using the protein domain sequence alignment showed that IPPT^Pfam^ and IPT^Pfam^ domain sequences formed individual clades with high branch support each (IPT^Pfam^: αLRT SH-like = 0.89, UFBT = 99; IPPT^Pfam^: αLRT SH-like = 0.85, UFBT = 82) and were highly supported sister to each other (αLRT SH-like = 0.97, UFBT = 100), suggesting that the origin of IPPT^Pfam^ and IPT^Pfam^ proteins could be traced back to before the emergence of the protein families (S3 Fig).

### IPPT^Pfam^ and IPT^Pfam^ domain proteins in plants and bacteria

The presence of IPPT^Pfam^ and IPT^Pfam^ domains assigned to *IPT*s was investigated across kingdoms including Archaea, bacteria, slime-mold, yeast, plants and animals. Intriguingly, IPT^Pfam^ domain genes were only found in the genomes of bacteria and the slime-mold *D*. *discoideum* (S1 Table), and in very few plant species: *P*. *patens*, *S*. *lycopersicum*, *S*. *tuberosum*, *Musa acuminata*, and *Oryza barthii* and *Oryza brachyntha* (S5 Table). On the other hand, IPPT^Pfam^ domain genes were found in most other organisms examined, except in Archaea and the *Mycoplasma* lineage in Firmicutes of bacteria (S1 Table, S4 Fig).

The bacterial IPT^Pfam^ domain genes (e.g., *Tzs* and *Ipt* in *A*. *tumefaciens*; S1 Fig) are well characterized, whereas those in plants only exist in a few species, many of those are located as very fragmented proteins shorter than 100 AA. These sequences matched only positions 2 to 112 of the 288 AA IPT^Pfam^.hmm, which indicated that they only retain the N-terminus region of IPT^Pfam^.hmm (S5 Table). In the seed alignment the IPT^Pfam^ domain was found to be about 40 AA longer than those of the IPPT^Pfam^ domain towards the N-terminus (Fig 1). Evaluation of the sequences in the IPPT^Pfam^ seed alignment showed that the IPPT^Pfam^ HMM profile can be extend towards the N-terminus to match the length of the IPT^Pfam^.hmm (Fig 1, S5 Fig). Thus, a new HMM alignment was built that included an additional 40 AA (IPPT^Pfam^_N40.hmm; S5 Fig). HMM searches revealed that plant IPT^Pfam^ domain gene sequences had a higher or equivalent similarity to IPPT^Pfam^_N40.hmm compared to IPT^Pfam^.hmm (S5 Table). The ML tree also showed that the plant IPT^Pfam^ domain genes grouped together in the IPPT^Pfam^ domain gene clade with a high support value (αLRT = 1), and not in the IPT^Pfam^ domain gene clade (S6 Fig). Therefore, the plant IPT^Pfam^ domains might be mis-assigned in the IPT^Pfam^ domains in the Pfam database since the original IPPT^Pfam^.hmm lacks the N-terminus region where IPPT^Pfam^ and IPT^Pfam^ domains have high similarities. However, our analyses indicated that those mis-assigned plant IPT^Pfam^ domains were more similar to IPPT^Pfam^ domain genes. Since those plant IPT^Pfam^ domains lack a functional annotation and are fragmental, these were excluded from further analyses.

Across bacteria, *D*. *discoideum*, and yeast, the phylogenetic analyses of IPPT^Pfam^ and IPT^Pfam^ domain genes showed that each clustered separately with maximal clade support (αLRT = 1; UFBT = 100) (Fig 2, S7 Fig). The bacterial IPPT^Pfam^ domain genes, termed *miaA*, clustered predominantly following species tree relationships (S4 Fig; see [39, 50]), except for those in ɛ-Proteobacteria (Ep) and *Borrelia burgdorferi* (Spirochaetes, Sp). IPT^Pfam^ domain genes were only found in a few species: in Proteobacteria (α-Proteobacteria: Al, ß-Proteobacteria: Be, γ-Proteobacteria: Ga) they formed a clade (αLRT = 0.99; UFBT = 100), and with Cyanobacteria (Cy) and Actinobacteria (Ac) in sister grades (Fig 2, S4 Fig). One gene of *D*. *discoideum* (amoeba: Am) was also assigned to the IPT^Pfam^ domain clade.

### Origin and diversification of *ISOPENTENYLTRANSFERASE*s

**Fig 4:**
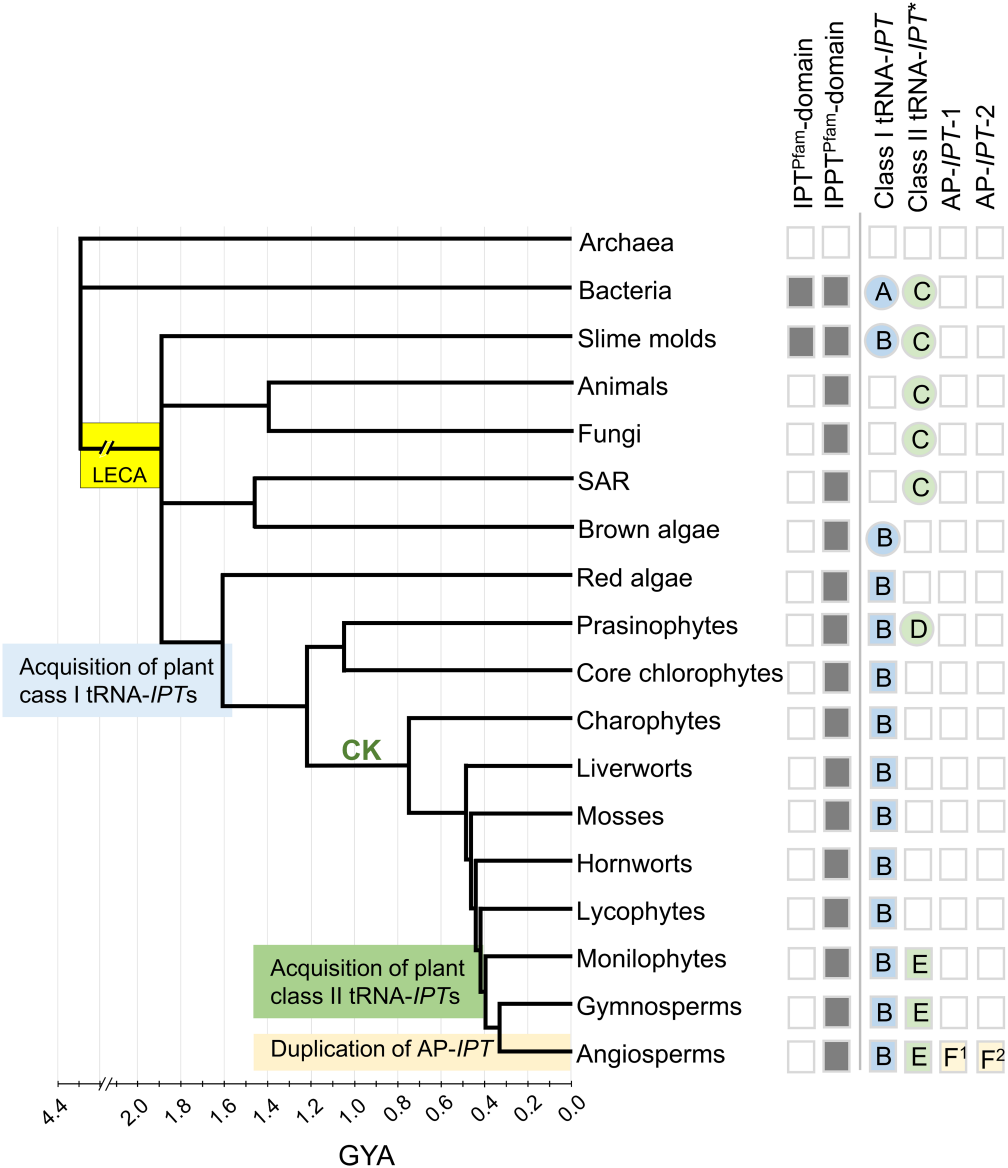
Schematic dated tree of life with absence and presence of IPT^Pfam^ and IPPT^Pfam^ domain genes and IPPT^Pfam^ gene clades/grades shown in Fig 3. Grey-shaded or open squares indicate IPT^Pfam^ and IPPT^Pfam^ domain presence or absence respectively. Presence (coloured squares) or absence (open squares) of class I tRNA-*IPT*s, class II tRNA-*IPT*s, and Adenosine-phosphate *IPT*s (AP-*IPT*s) for plants indicated by blue, green, or orange respectively. Class I and class II tRNA-*IPT*s and AP-*IPT*s are IPPT^Pfam^ domain genes. Shaded-circles indicate the presence of the possible direct ancestral IPPT^Pfam^ domain genes of plant class I and class II tRNA-*IPT*s. LECA: the last eukaryotic common ancestor, CK: point of cytokinin signal establishment [51]. Organism phylogeny is based on the Tree of Life Web Project [52], Qiu et al. [53] and Hug et al. [54], Popper et al. [55], Derelle et al. [56]. Dates are transferred from Magallón et al. [44], Heron et al. [45], Parfrey et al. [46], Battistuzzi et al. [39].

The cytokinin synthesizing *IPT*s in the plant species examined here all retained IPPT^Pfam^ domains (S1 and S6 Tables). In the phylogenetic tree rooted on IPT^Pfam^ domain genes (αLRT = 1; UFBT = 100), the IPPT^Pfam^ domain genes formed a maximally supported clade (αLRT = 1; UFBT = 100) and could be divided into two grades and four clades with mostly high branch support (Fig 3, Table 1, S8 Fig). The bacterial *miaA* genes formed grades at the base of the IPPT^Pfam^ clade and each of the two IPPT^Pfam^ subclades, one leading to plant class I tRNA-*IPT*s (Fig 3 clade B, S1 Table), the other to Unikont-SAR tRNA-*IPT*s including animal, fungi, zooplankton, and some copies from slime-mold (Fig 3 grade C). The prasinophyte tRNA-*IPT*s followed in the next grade (Fig 3 clade D), to which euphyllophyte *IPT*s were sister (Fig 3 clades E + F). Class II tRNA-*IPT*s (Fig 3 clade E) included genes from euphyllophytes, i.e. monilophytes, gymnosperms, and angiosperms. The clade and grade structures shown in Fig 3 is summarized along the tree of life in Fig 4.

### Duplications of *ISOPENTENYLTRANSFERASEs* within plant clades

The high copy number of IPPT^Pfam^ genes found in mosses and angiosperms had different patterns of distribution: the mosses *Sphagnum fallax* and *Physcomitrella patens* possessed five and eight IPPT^Pfam^ genes respectively, all of which belonged to the class I tRNA-*IPT* clade (‘Mosses’ in Fig 3). Most angiosperms in this clade, on the other hand, had only single copies, except for *Brassica rapa* and *Sorghum bicolor* which had two copies. Angiosperms, however, possessed additional IPPT^Pfam^ genes across the class II tRNA-*IPT*s, and a high-copy number in the AP-*IPT*s clade (Fig 3, S1 and S2 Tables). The basal angiosperm *Amborella trichopoda* possessed two copies of AP-*IPT*s and each was assigned to a different clade (black arrows in Fig 3 clade F), where otherwise extensive gene duplications had occurred. For instance, *A*. *thaliana* possessed four genes in clade F^1^ and three in clade F^2^, and *Oryza sativa* three in F^1^ and five in F^2^ respectively. Within the clades the gene trees roughly followed the species tree with some discrepancies, but many of these branches were not highly supported or unsupported (S8 Fig). The NOTUNG analyses provided some context for the interpretation of these discrepancies.

**Fig 5:**
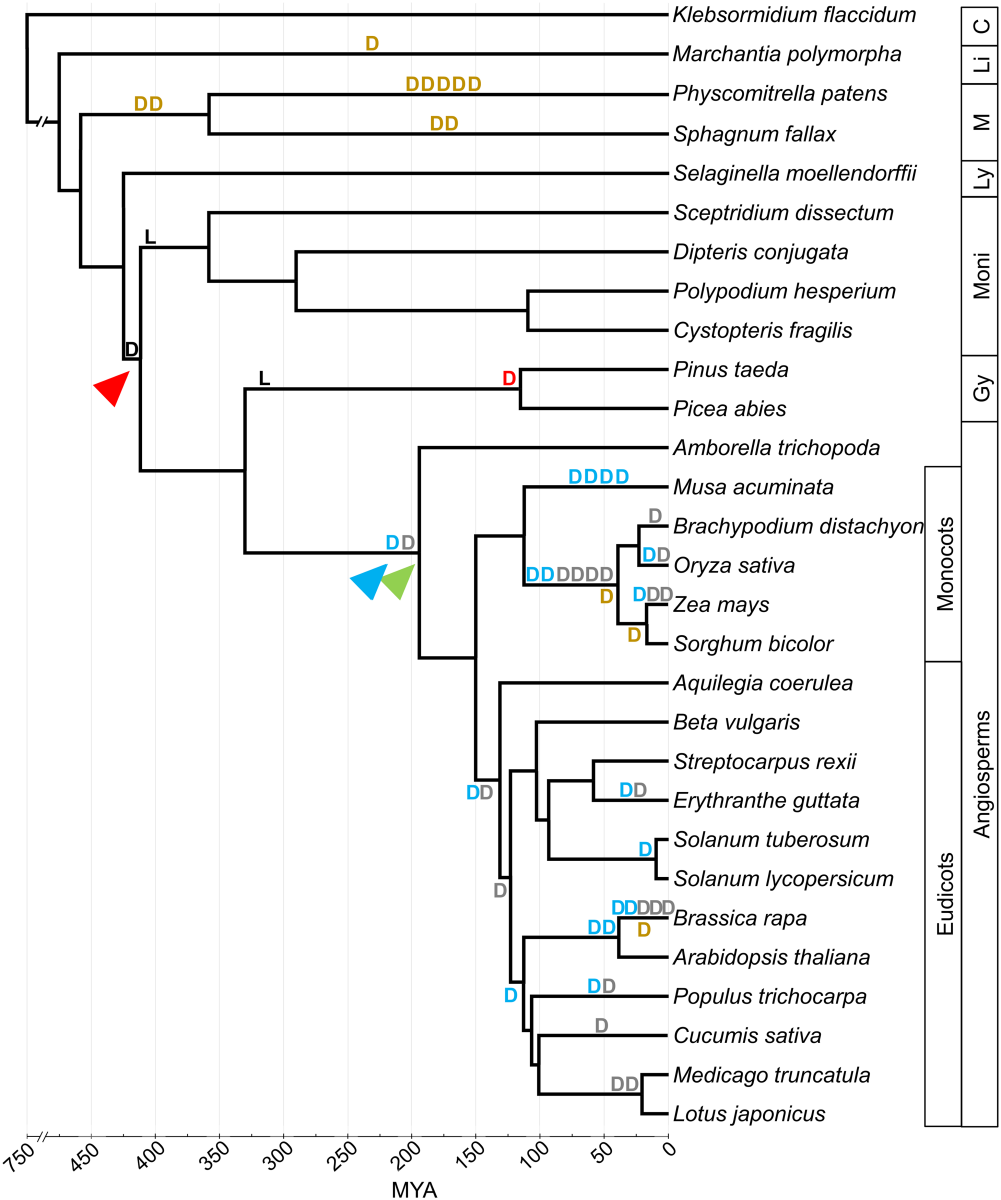
Duplications and major losses in *IPT* genes inferred in NOTUNG analyses on the tree of life for plants. Gene duplication resulting in class II tRNA-*IPT* and AP-*IPT* (red arrowhead), was followed by AP-*IPT* losses in ferns and gymnosperms (`L`in black). AP-*IPT*s duplications were inferred before or at angiosperm diversification (blue and green arrowheads). D: gene duplications, L: gene losses, `D`in black: duplication leading to class II tRNA-*IPT* and AP-*IPT*, `D`in blue: duplications within AP-*IPT*-1, `D`in grey: duplications within AP-*IPT*-2, `D`in red: duplication within class II tRNA-*IPT*, `D`in brown: duplications within class I tRNA-*IPT*.

The reconciled NOTUNG tree for plant class I tRNA-*IPT* genes had a DL score (duplications and losses event score) of 48, and suggested 18 duplications and 21 losses. Rearranging the tree topology around poorly supported branches resulted in a greatly reduced DL score of 23.5, with 13 duplications and 4 losses (S11 Fig). Most duplications were inferred in the moss lineage, with two of the nine occurring at the time of diversification of *Physcomitrella patens* and *Sphagnum fallax* and alone five in *P*. *patens* after its diversification from *S*. *fallax* (Fig 5). Isolated duplications of class I tRNA-*IPT* inferred to have occurred once in *Marchantia polymorpha*. In angiosperms, class I tRNA-*IPT* duplications were rarely inferred, once prior or at the time of diversification of Poaceae, once within Poaceae at or prior to the split between *Zea mays* and *Sorghum bicolor*, and once in *Brassica rapa* after its split from *A*. *thaliana* (Fig 5).

For class II tRNA-*IPT*s/AP-*IPT*s, the reconciled NOTUNG tree prior rearrangement had a DL score of 220.5, involving 61 duplications and 129 losses. After rearrangement, the DL score was reduced to 88.5 with 39 duplications and 30 losses (S14 Fig). One early duplication of class II tRNA-*IPT* and AP-*IPT* was inferred to have occurred after the acquisition of *IPT* genes by euphyllophytes perhaps coinciding with the diversification of the lineage (S14 Fig, Figs 4 and 5, red arrowhead), with the monilophytes and gymnosperms appear to have consecutively lost their AP-*IPT* copies. Two successive duplications were inferred for angiosperms prior or at the time of their first diversification, the first giving rise to AP-*IPT*-1 (Fig 3F^1^) and AP-*IPT*-2 (Fig 3F^2^), the following one resulting in AP-*IPT*-2a (Fig 3F^2^a) and AP-*IPT*-2b (Fig 3F^2^b). Some lineages such as *Amborella trichopoda* and monocots were inferred to have lost their AP-*IPT*-2b copy (S14 Fig). More local duplications are scattered across the angiosperms. The monocot lineage Poaceae and Brassicaceae showed a high clustering of duplications, with the former having six duplication events prior or at the time of diversification and five such events were inferred for the lineage of *Brassica rapa* (Fig 5).

The exon-intron structure showed that class I and class II tRNA-*IPT*s possessed multiple introns, but in Poaceae intron losses occurred in class I tRNA-*IPT*s (S15 Fig, S2 Table). Unlike tRNA-*IPT*s, AP-*IPT*s in general rarely possessed introns (S15 Fig, S2 Table). To understand the differentiation and similarities of function of the multiple copies of *IPT*, published results for gene expression patterns in moss, gymnosperm, and angiosperms were summarised alongside the phylogenetic IPT^Pfam^/IPPT^Pfam^ tree (S15 Fig).

## Discussion

### IPPT^Pfam^ and IPT^Pfam^ domains

The Pfam database v.31.0 (released on 8 March 2017) contains 16,712 protein families and 604 clans. Each family is based on the manually curated seed-alignment of protein domains and thus each has a unique Hidden Markov Model (HMM) profile. A Pfam clan is a structural unit of families that share a related structure, function, and significantly matching HMM profile, suggesting that they have a single evolutionary origin [57, 58]. The two protein families, IPPT^Pfam^ and IPT^Pfam^, assigned for cytokinin biosynthesis *IPT* genes are both in the P-loop NTPase clan and closely related, suggesting that genes in the IPPT^Pfam^ and IPT^Pfam^ families share a common ancestor before the two protein families diverged, and followed independent evolutionary trajectories. This has been confirmed here in our analysis including the VirE^Pfam^ family (S3 Fig).

IPT^Pfam^ domain genes are only found in a few bacteria, whereas IPPT^Pfam^ domain genes are found in most organisms except the Archaea and *Mycoplasma* lineages. It is unclear whether IPPT^Pfam^ is lost in Archaea or gained in bacteria since the relationships between the two groups are still unclear (e.g. [52]). It appears, however, to more likely represent a gain in bacteria that spread into the eukaryote lineages (see e.g. [49]). The Firmicute *Mycoplasma* is known to have a very small genome that is missing many genes, which might be a reason for the absence of IPPT^Pfam^ domain genes here [59].

The IPT^Pfam^ domain genes are phylogenetically scattered and found only in some members of Actinobacteria, Cyanobacteria, ɑ-Proteobacteria, β-Proteobacteria, and γ-Proteobacteria and in the eukaryote *D*. *discoideum*. The IPT^Pfam^ domain clade showed long branches and its topology was mostly congruent with the species tree. One could hypothesize that they were present in the ancestor of bacteria, and as a result of a strong evolutionary selection only the plant pathogenic lineages retained the IPT^Pfam^ domain genes, perhaps because of the importance of cytokinins in plant pathogenicity (e.g. [60]). However, this would require multiple losses of IPT^Pfam^ domain genes in the other bacteria lineages. Overall, a more parsimonious scenario would be HGTs that caused the scattered distribution of IPT^Pfam^ domain genes in bacteria, perhaps events that occurred in the more distant past that allowed some phylogenetic patterns to be retained among the IPT^Pfam^ domain genes. In support of this scenario, *D*. *discoideum* could be cited where HGT events are widely observed in its genome and this might explain the presence of IPT^Pfam^ in this organism [61].

One might expect that cytokinin synthesising genes in bacteria and plants are closely related. However, bacteria and slime-mold cytokinin synthesising *IPT*s appear to be only distantly related to plant *IPT*s. Plant IPPT^Pfam^ domain *IPT*s were found indeed closer related to bacteria IPPT^Pfam^ domain *miaA* genes that however, do not synthesise cytokinins (Fig 3). Thus we infer that the cytokinin synthesis pathways in plants and bacteria have evolved or have been acquired twice independently.

### Origins and early evolution of *ISOPENTENYLTRANSFERASEs*

**Fig 6:**
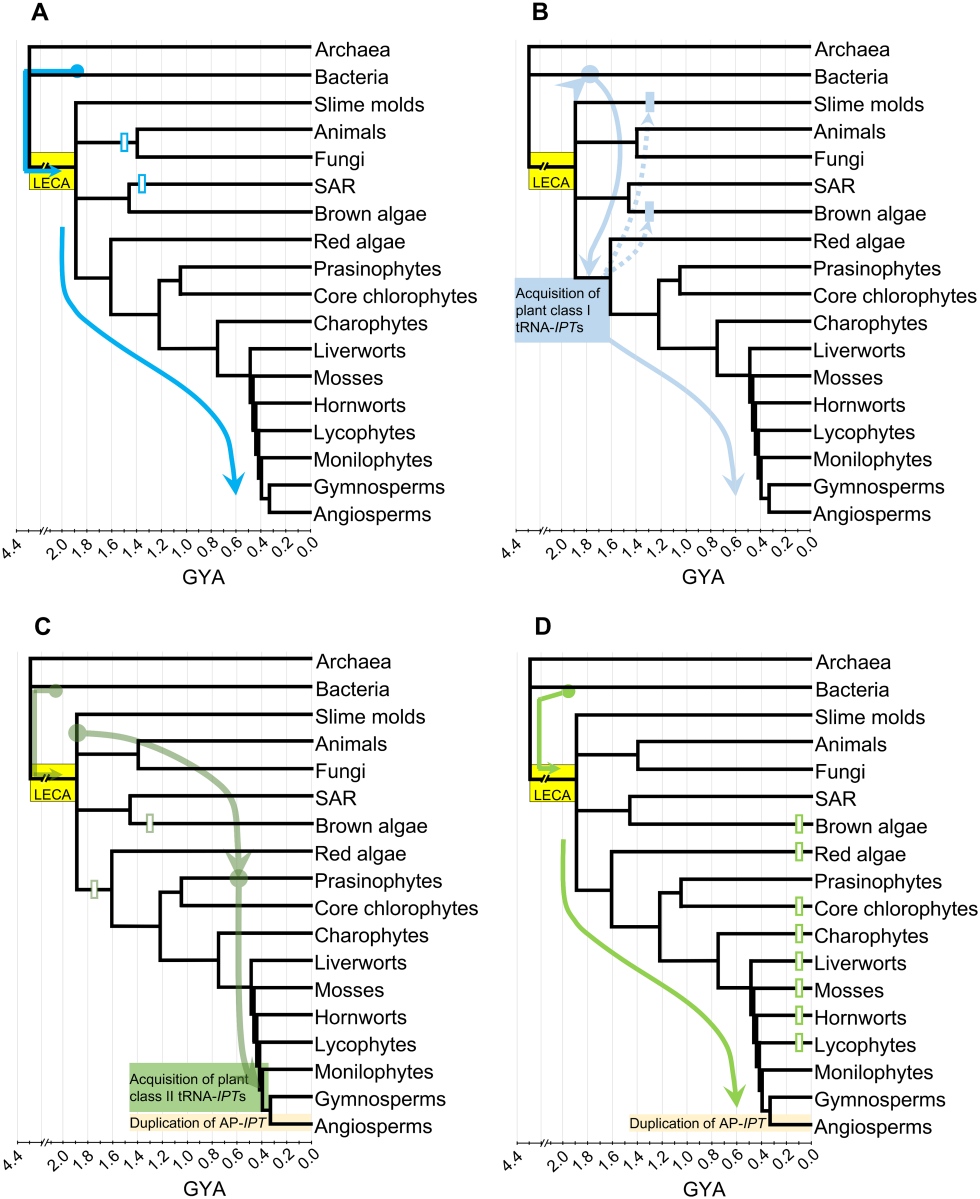
Schematic summary of hypotheses for *ISOPENTENYLTRANSFERASE* gene evolution inferred in this study. Lines indicate possible evolutionary pathways from bacterial or eukaryotic ancestral IPPT^Pfam^ domain genes to plant *IPT* genes with IPPT^Pfam^ domain. Open boxes: gene loss, shaded boxes: gene gain, LECA: the last eukaryotic common ancestor. **A, B**. Hypotheses for class I tRNA-*IPT* evolution. **A**. class I tRNA-*IPT*s in plants directly descended from LECA gene and loss in Unikont and SAR independently. **B**. class I tRNA-*IPT*s in plants acquired via HGT from bacteria and secondary transfer to brown algae and slime molds. **C, D**. Hypotheses for class II tRNA-*IPT*/AP-*IPT* evolution. **C**. class II tRNA-*IPT*s in euphyllophytes were obtained via HGT from eukaryotic organisms, using prasinophytes as stepping stone. **D**. class II tRNA-*IPT*s evolved directly from LECA, but loss in brown algae, red algae and in several basal lineages of green plants independently.

The present study has shown that plants *IPT*s have two different evolutionary sources, class I tRNA-*IPT*s originating from bacterial *miaA* genes, and class II tRNA-*IPT*s and AP-*IPT*s linked to the Unikont-SAR *IPT* grade (Fig 3C) through prasinophyte algae tRNA-*IPT*s (Fig 3D). The class I tRNA-*IPT* clade included all plant lineages examined in this study, ranging from red algae to angiosperms. The basal relationships of the tree of life around the last eukaryotic common ancestor (LECA) are still unresolved which somewhat hampers the clarification of the origin of *IPT* genes as well as the limited sampling in non-plant lineages in this study. However, based on the distribution of the genes among lineages (Figs 3–5), several hypotheses can be proposed (Fig 6): It is possible that plants have acquired class I tRNA-*IPT* genes from bacteria through their LECA early on in time 1,900 MYA and then following the tree of life with subsequent losses in the lineages leading to animals/fungi (Unikonts) and SAR (Fig 6A). Alternatively, plants could have acquired class I tRNA-*IPT*s via HGT from bacteria, perhaps before the diversification of plantae 1,600 MYA (Fig 6B). In this case, the brown algae and slime mold lineage would have acquired the genes independently, perhaps through further HGT events.

Also for the origin of plant class II tRNA-*IPT*/AP-*IPT*, two hypotheses for can be postulated (Fig 6C and 6D): In one hypothesis, a common ancestor of the red algae and green plants (green lineage) lost the original eukaryotic tRNA-*IPT* of the LECA, and around 411 MYA, euphyllophytes secondarily acquired class II tRNA-*IPT*s by two HGT events from Unikont-SAR tRNA-*IPT* using the prasinophyte algae as stepping stone (Fig 6C). This hypothesis is supported by the unique genome structure of prasinophyte algae. It harbours large viral DNA in addition to their own genome [62, 63], and HGT events are commonly observed between eukaryote genomes and viral DNAs [62, 63, 64]. In an alternative hypothesis, class II tRNA-*IPT*/AP-*IPT* could have originated by descent of the original eukaryotic tRNA-*IPT* from the LECA, following the tree of life to the green lineages, but was later lost in several plant lineages (Fig 6D). However, this would require seven independent losses of the genes, in red algae, core chlorophytes and charophytes in green algae, liverworts, mosses, hornworts, and lycophytes (Fig 6D). The fact that the publicly available 14 genomes of the seven lineages investigated here all lack class II tRNA-*IPT* genes might suggest that the stepping-stone hypothesis is more likely because it requires fewer events to explain the scenario. There is some controversy surrounding the paraphyly of bryophytes, with the latest work suggesting various scenarios [65, 66]. Even if they were monophyletic, this would reduce the number of losses of class II tRNA-*IPT* genes by only two. Overall, a better understanding of the deep origin of tRNA-*IPT* genes can only be gleaned once the number of available genomes increases in the future and a better resolution of the eukaryote origin is achieved.

Among plants, only prasinophyte algae, monilophytes, gymnosperms and angiosperms possessed additional tRNA-*IPT*s besides class I tRNA-*IPT*s. In a previous study, these were classified together as class II tRNA-*IPT*s [19]. The present study showed that prasinophyte algae tRNA-*IPT*s formed a grade between Unikont-SAR tRNA-*IPT*s, and a clade with plant class II tRNA-*IPT*s and AP-*IPT*s. None of the other algae lineages (i.e. red algae, core chlorophytes, charophytes), bryophytes, and lycophytes retained class II tRNA-*IPT*s and AP-*IPT*s (Fig 4, S1 Table). A study on the evolution of cytokinin receptor genes suggested that the cytokinin signal transduction pathway established later towards the evolution of land plants in charophytes. Since prasinophytes algae lack the complete set of genes responsible for cytokinin signal transductions [51, 67], the additional copies of tRNA-*IPT*s in prasinophyte algae might not possess the function for cytokinin production but have their own as yet unknown roles. Therefore, in this study prasinophyte algae tRNA-*IPT*s (Fig 3D) were placed in their own class, `prasinophyte tRNA-*IPT*s`, separate from plant class II tRNA-*IPT*s (Table 1).

### Duplication and redundancy of plant *ISOPENTENYLTRANSFERASEs*

The evolutionary history of *IPT*s in plants is marked by multiple gene duplication and major loss events that strikingly differed between plant lineages (Figs 3 and 5). It was noticeable that class I tRNA-*IPT*s showed many duplications in mosses, and very few in angiosperms, while the reverse was the case for class II tRNA-*IPT*/AP-*IPT* genes. This might be linked to functional redundancies (see below). The time of acquisition of a second set of tRNA-*IPT*s in euphyllophytes was estimated to around 411 MYA, sometime after the emergence of land plants [68], and coincided with a gene duplication event that gave rise to class II tRNA-*IPT* and AP-*IPT*. The latter was apparently lost in monilophytes and gymnosperms (Figs 4 and 5, S14 Fig), or not yet found at least in gymnosperms where only two genomes of one family, Pinaceae, were available at present. Two further duplications among AP-*IPT*s led to a further increase in copy numbers around the time of first divergence of angiosperms 194 MYA. Further duplications occurred, often in parallel in AP-*IPT*-1 and AP-*IPT*-2 throughout the diversification of angiosperms (Figs 3–5). Some of the earlier events might be linked to whole genome duplications that have been indicated in the evolution of seed plants and angiosperms (e.g. [69]]. The strong clustering of duplication events in Brassicaceae and Poaceae may stem from the much denser genome data available for these lineages that included model plants such as *A*. *thaliana* or *O*. *sativa*.

Overall, the pattern of *IPT* gene duplications across plants showed a tendency of an increased rate towards derived clades and increased morphological complexity with a peak in the AP-*IPT* clade with some plants possessing more than 10 copies (Figs 3 and 5, S1 Table). Comparing the function of these copies indicated that some *IPT*s show ubiquitous expression, while others show tissue specific patterns, and great redundancies among copies (S15 Fig; [14, 20]). A tendency was observed in that copies with specific roles occur in the most derived class of *IPT* genes in each species. For mosses it was the class I tRNA-*IPT*s, for gymnosperm class II tRNA-*IPT*s, and for angiosperm AP-*IPT*s; e.g. suppression of *PpIPT4* expression in the moss sporophytic stage (S9 Fig; [21]), differential expression of *PatIPT_IIa* and *PatIPT_IIb* in female cones (S9 Fig; [70]), and in angiosperms, AP-*IPT*s showed differential expression patterns in different organs and differential response to external cytokinin treatments (S15 Fig, S4 Table). This might be a typical pattern for gene duplications from a ubiquitously expressed copy that allowed the acquisition of redundant copies to have a specific roles [71]. Thus, multiple but specific plant *IPT* copies may be important in fine-tuning the cytokinin concentration locally.

Introns are rarely found in AP-*IPT*s in contrast to class II tRNA-*IPT*s (S15 Fig). Considering the more likely stepping stone origin for class II tRNA-*IPT*s through prasinophytes, the lack of introns in prasinophytes might indicate that intron-gain in plant class II-*IPT*s is more likely (Figs 2 and 5, S9 Fig) rather than the intron-loss in AP-*IPT*s. The expression of AP-*IPT*s with few or no introns might be regulated by specific promoters reacting in the temporal-spatial manner at different plant growth stages (e.g. [20]). Considering the effects of presence and absence of introns, it was shown that rapidly transcribed genes retained lower numbers of introns [72]. It can be speculated that intron-less AP-*IPT* genes might result in more rapid transcription during different developmental stages when a finely tuned rapid cytokinin production is required, for example during flower development or when responding rapidly to environmental changes (e.g. [73]). A unique case was found in the Poales clade showing an absence of introns in class I tRNA-*IPT*s, whereas other lineages retained introns. While AP-*IPT*s produce *trans*-zeatin or isopentenyladenine type cytokinins, which have been considered as major cytokinins in angiosperms, tRNA-*IPT*s are thought to produce *cis*-zeatin type cytokinin, which is supposed to have minor or no function as cytokinin [3]. However, *cis*-zeatins are abundant in Poales [52, 74] and even retain their biological functions as cytokinins [75]. It might just be that intron loss in Poales class I tRNA-*IPT*s affect the regulation of *cis*-zeatin type cytokinin production in plants, an aspect that would be worthwhile testing in the future.

## Conclusions

The roles and functions of *ISOPENTENYLTRANSFERASEs*, key genes for the production of cytokinins, have been studied intensively over the last two decades. The accumulating genome knowledge of model and non-model plants and an accompanying advancement in statistical analytical methodology applied here allowed us to reveal the phylogenetic origin and evolution of these genes across the tree of life. This study revealed that plant *IPT*s are closely related to bacteria *miaA* genes (IPPT^Pfam^) and not to bacteria *IPT* genes (IPT^Pfam^). Further, plants possess two independent *IPT*s, class I tRNA-*IPT*s and class II tRNA-*IPT*/AP-*IPT*s. Their exact deep origin could not be fully resolved due to uncertain relationships in basal eukaryotes. However, class II tRNA-*IPT*s and AP-*IPT*s are the consequence of a gene duplication event at the onset of euphyllophyte diversification. Further gene duplication events in the plant lineage were inferred with increasing frequency towards angiosperms, coinciding with emerging increased specialisation of functions. This study is an example for the elucidation of the deep history of cytokinin synthesis genes that involved an interplay of possible horizontal gene transfers, gene duplications, losses and diversification in function in the evolution of a multigene family.

## Supporting information

S1 Fig Domain structure of *ISOPENTENYLTRANSFERASEs* in *Arabidopsis thaliana* and *Tzs*, *Ipt*, and *miaA* genes in *Agrobacterium tumefaciens*. Domains are shown as green boxes. Coordinates to the Pfam HMM profiles are shown below the boxes.
(PDF)

S2 Fig Neighbor-joining tree of HMM profiles of P-loop NTPases in the Pfam database. The tree was calculated by pHMM-tree. IPT^Pfam^ and IPPT^Pfam^ families appeared as sister clades (arrow).
(PDF)

S3 Fig ML tree based on sequences of IPPT^Pfam^, IPT^Pfam^, and pVirE^Pfam^ seed alignments. The tree is rooted on VirE^Pfam^ sequences. The αLRT SH-like values (left) and UFBT values (right) are shown on major branches leading to each protein family. Branches with above 70% support values are emphasized by a thick line.
(PDF)

S4 Fig Species tree of bacteria. Species retaining the IPPT^Pfam^ domain gene are shown in black, for species with both IPPT^Pfam^ and IPT^Pfam^ domain genes in orange, and for species lacking IPPT^Pfam^ and IPT^Pfam^ domain genes in grey.
(PDF)

S5 Fig Comparison of HMM logos between the original HMM registered in Pfam v.31.0 and the expanded HMM build in this study. IPT^Pfam^.hmm and the original IPPT^Pfam^.hmm (1247 seed seq) were retrieved from Pfam v.31.0. IPPT^Pfam^.hmm (103 seed seq) built with 103 representative out of 1247 seed sequences. Logos build from 1247 sequences in the original seed alignment and the 103 representative sequences were very similar. New HMM profile with extended N-terminus (IPPT^Pfam^_N40.hmm) built in this study. N40: additional N-terminus region in IPPT^Pfam^_N40.hmm.
(PDF)

S6 Fig ML tree calculated by PhyML including plant sequences registered in the IPT^Pfam^ family of Pfam database. Plant sequences in IPT^Pfam^ family of Pfam indicated by red arrows, and those shown in the IPPT^Pfam^ domain clade but not in the IPT^Pfam^ clade (αLRT SH-like = 1).
(PDF)

S7 Fig ML tree shown in Fig 2 with all support values. The αLRT (left) and UFBT values (right) are shown along major branches. An asterisk indicates support values < 0.5 and < 50%. Thickened branches indicate support values > 0.9 and > 90%, medium-thick branches indicate > 0.7 and > 70%. The classification of the species is indicated by two characters at the end of the gene names; Ac: Actinobacteria, Al: α-Proteobacteria, Am: Amoebozoa, Aq: Aquficae, As: Ascomycota, Be: β-Proteobacteria, Ch: Chlamydiae, Cy: Cyanobacteria, Ep: ε-Proteobacteria, Fi: Firmicutes, Fu: Fusobacteria, Ga: γ-Proteobacteria, Sp: Spirochaetes, Th: Thermotogae.
(PDF)

S8 Fig ML tree shown in Fig 3 with all support values. IPT^Pfam^ domain genes were used as outgroup. The αLRT (left) and UFBT support values (right), are shown along the major branches. An asterisk indicates support values < 0.5 and < 50%. Thickened branches indicate support values > 0.9 and > 90%, medium-thick branches indicate > 0.7 and > 70%.
(PDF)

S9 Fig Species tree used for NOTUNG analyses of plant class I tRNA-*IPT*s. (PDF)

S10 Fig Gene tree used for NOTUNG analyses of plant class I tRNA-*IPT*s. (PDF)

S11 Fig NOTUNG DL analyses of plant class I tRNA-*IPT*s. Weak edges highlighted yellow. Gene duplications marked by red ‘D’.
(PDF)

S12 Fig Species tree used for NOTUNG analyses of plant class II tRNA-*IPT*s/AP-*IPT*s. (PDF)

S13 Fig Gene tree used for NOTUNG analyses of plant class II tRNA-*IPT*s/AP-*IPT*s. (PDF)

S14 Fig NOTUNG DL analyses of plant class II tRNA-IPTs/AP-*IPT*s. Weak edges highlighted yellow. Gene duplication marked by red ‘D’.
(PDF)

S15 Fig Summary of intron positions, expression patterns, cytokinin interaction of plant *ISOPENTENYLTRANSFERASEs* alongside the phylogenetic tree. The tree is a cladogram of the tree shown in Fig 3. Intron positions are shown as schematic illustrations. Asterisks indicate absence of introns in the gene. Genes without intron information are shown with ‘?’. Gene expressions are shown in square boxes: red indicates strong expression, orange indicates medium expression or expression without quantification, white indicates very weak or no expression in the tissues indicated (see also S3 Table). The response to external cytokinin treatments are indicated by upper or lower triangles: Upper triangles indicate the responses in the above ground parts of plants, and lower triangles indicate the responses in roots. Increase in gene expression is shown in yellow, no change in blue, and reduced expression in white (see also S4 Table).
(PDF)

S1 Table List of species used in this study and their classification, with the numbers of IPT^Pfam^ and IPPT^Pfam^ domain genes. (PDF)

S2 Table Gene accession numbers used in this study. Gene ID is the ID used in the large phylogeny in Fig 3. Asterisks indicate the gene name retrieved from Frébort et al. (2011) [18].
(PDF)

S3 Table References used for the summary of gene expressions in S15 Fig. (PDF)

S4 Table References used for cytokinin interactions in S15 Fig. (PDF)

S5 Table List of plant IPT^Pfam^ domain genes in the Pfam database and results of the hmmsearch. (PDF)

S6 Table Domains assigned in *ISOPENTENYLTRANSFERASE* genes in model plants and cytokinin biosynthesizing bacteria. (PDF)

## References

[1] Ohta T. Evolution of gene families. Gene. 2000; 259: 45–52. 11163960 10.1016/s0378-1119(00)00428-5

[2] Jiao Y, Wickett NJ, Ayyampalayam S, Chanderbali AS, Landherr L, Ralph PE, et al Ancestral polyploidy in seed plants and angiosperms. Nature. 2011; 473: 97–100. 10.1038/nature09916 21478875

[3] Sakakibara H. Cytokinins: Activity, Biosynthesis, and Translocation. Ann Rev Plant Biol. 2006; 57: 431–449. 16669769 10.1146/annurev.arplant.57.032905.105231

[4] Chimnaronk S, Forouhar F, Sakai J, Yao M, Tron CM, Atta M, et al Snapshots of dynamics in synthesizing *N*^*6*^-Isopentenyladenosine at the tRNA anticodon. Biochemistry. 2009; 48:5057–5065. 10.1021/bi900337d 19435325 PMC2786004

[5] Schweizer U, Bohleber S, Fradejas-Villar N. The modified base isopentenyladenosine and its derivatives in tRNA. RNA Biol. 2017; 17: 1–12. 10.1080/15476286.2017.1294309 PMC5699536 28277934

[6] Kado CI. Historical account on gaining insights on the mechanism of crown gall tumorigenesis induced by *Agrobacterium tumefaciens*. Front Microbiol. 2014; 5: 340 10.3389/fmicb.2014.00340 25147542 PMC4124706

[7] Frébortova J, Greplova M, Seidl MF, Heyl A, Frebort I. Biochemical characterization of putative adenylate dimethylallyltransferase and cytokinin dehydrogenase from *Nostoc sp*. PCC 7120. PLoS One. 2015; 10: e0138468 10.1371/journal.pone.0138468 26376297 PMC4574047

[8] Nomura T, Tanaka Y, Abe H, Uchiyama M. Cytokinin activity of discadenine: A spore germination inhibitor of *Dictyostelium discoideum*. Phytochemistry. 1977; 16: 1819–1820.

[9] Akiyoshi DE, Klee H, Amasino RM, Nester EW, Gordon MP. T-DNA of *Agrobacterium tumefaciens* encodes an enzyme of cytokinin biosynthesis. Proc Natl Acad Sci USA. 1984; 81: 5994–5998 6091129 10.1073/pnas.81.19.5994 PMC391845

[10] Barry GF, Rogers SG, Fraley RT, Brand L. Identification of a cloned cytokinin biosynthetic gene. Proc Natl Acad Sci USA. 1984; 81: 4776–4780. 16593495 10.1073/pnas.81.15.4776 PMC391573

[11] Takei K, Sakakibara H, Sugiyama T. Identification of genes encoding adenylate isopentenyltransferase, a cytokinin biosynthesis enzyme, in *Arabidopsis thaliana*. J Biol Chem. 2001; 276: 26405–26410. 10.1074/jbc.M102130200 11313355

[12] Kakimoto T. Identification of plant cytokinin biosynthetic enzymes as dimethylallyl diphosphate:ATP/ADP isopentenyltransferases. Plant Cell Physiol. 2001; 42: 677–685. 11479373 10.1093/pcp/pce112

[13] The Arabidopsis Genome Initiative. Analysis of the genome sequence of the flowering plant *Arabidopsis thaliana*. Nature. 2000; 408: 796–815. 10.1038/35048692 11130711

[14] Miyawaki K, Matsumoto-Kitano M, Kakimoto T. Expression of cytokinin biosynthetic isopentenyltransferase genes in *Arabidopsis*: tissue specificity and regulation by auxin, cytokinin, and nitrate. Plant J. 2004; 37: 128–138. 14675438 10.1046/j.1365-313x.2003.01945.x

[15] Sakamoto T, Sakakibara H, Kojima M, Yamamoto Y, Nagasaki H, Inukai Y, et al Ectopic expression of KNOTTED1-like homeobox protein induces expression of cytokinin biosynthesis genes in rice. Plant Physiol. 2006; 142: 54–62. 10.1104/pp.106.085811 16861569 PMC1557621

[16] Yevdakova NA, von Schwartzenberg K. Characterisation of a prokaryote-type tRNA-isopentenyltransferase gene from the moss *Physcomitrella patens*. Planta. 2007; 226: 683–695. 10.1007/s00425-007-0516-0 17450376

[17] Matsuo S, Kikuchi K, Fukuda M, Honda I, Imanishi S. Roles and regulation of cytokinins in tomato fruit development. J Exp Bot. 2012; 63: 5569–5579. 10.1093/jxb/ers207 22865911 PMC3444270

[18] Frébort I, Kowalska M, Hluska T, Frébortova J, Galuszka P. Evolution of cytokinin biosynthesis and degradation. J Exp Bot. 2011; 62: 2431–2452. 10.1093/jxb/err004 21321050

[19] Lindner AC, Lang D, Seifert M, Podlesakova K, Novak O, Strnad M, et al Isopentenyltransferase-1 (IPT1) knockout in *Physcomitrella* together with phylogenetic analyses of IPTs provide insights into evolution of plant cytokinin biosynthesis. J Exp Bot. 2014; 65: 2533–2543. 10.1093/jxb/eru142 24692654 PMC4036517

[20] Miyawaki K, Tarkowski P, Matsumoto-Kitano M, Kato T, Sato S, Tarkowska D, et al Roles of Arabidopsis ATP/ADP isopentenyltransferases and tRNA isopentenyltransferases in cytokinin biosynthesis. Proc Natl Acad Sci USA. 2006; 103: 16598–16603. 10.1073/pnas.0603522103 17062755 PMC1637627

[21] Patil G, Nicander B. Identification of two additional members of the tRNA isopentenyltransferase family in *Physcomitrella patens*. Plant Mol Biol. 2013; 82: 417–426. 10.1007/s11103-013-0072-x 23712255

[22] Blackwell JR, Horgan R. Cloned *Agrobacterium tumefaciens ipt1* gene product, DMAPP:AMP isopentenyl transferase. Phytochemistry. 1993; 34: 1477–1481.

[23] Page RDM, Holmes EC. Molecular Evolution, A Phylogenetic Approach. Oxford: Blsckwell Science Ltd; 1998.

[24] Lee EK, Cibrian-Jaramillo A, Kolokotronis SO, Katari MS, Stamatakis A, Ott M, et al A functional phylogenomic view of the seed plants. PLoS Genet. 2011; 7: e1002411 10.1371/journal.pgen.1002411 22194700 PMC3240601

[25] Soltis DE, Smith SA, Cellinese N, Wurdack KJ, Tank DC, Brockington SF, et al Angiosperm phylogeny: 17 genes, 640 taxa. Am J Bot. 2011; 98: 704–730. 10.3732/ajb.1000404 21613169

[26] The Angiosperm Phylogeny Group. An update of the Angiosperm Phylogeny Group classification for the orders and families of flowering plants: APG IV. Bot J Linnean Soc. 2016; 181: 1–20.

[27] Finn RD, Bateman A, Clements J, Coggill P, Eberhardt RY, Eddy SR, et al Pfam: the protein families database. Nucleic Acids Res. 2014; 42: D222–D230. 10.1093/nar/gkt1223 24288371 PMC3965110

[28] Eddy S. A probabilistic model of local sequence alignment that simplifies statistical significance estimation. PLoS Comput Biol. 2008; 4: e1000069 10.1371/journal.pcbi.1000069 18516236 PMC2396288

[29] Huo L, Zhang H, Huo X, Yang Y, Li X, Yin Y. pHMM-tree: phylogeny of profile hidden Markov models. Bioinformatics. 2017; 33: 1093–1095. 10.1093/bioinformatics/btw779 28062446 PMC5860389

[30] Criscuolo A, Gribaldo S. BMGE (Block Mapping and Gathering with Entropy): a new software for selection of phylogenetic informative regions from multiple sequence alignments. BMC Evol Biol. 2010; 10: 210 10.1186/1471-2148-10-210 20626897 PMC3017758

[31] Price MN, Dehal PS, Arkin AP. FastTree: computing large minimum evolution trees with profiles instead of a distance matrix. Mol Biol Evol. 2009; 26: 1641–1650. 10.1093/molbev/msp077 19377059 PMC2693737

[32] Katoh K, Standley DM. MAFFT multiple sequence alignment software version 7: improvements in performance and usability. Mol Biol Evol. 2013; 30: 772–780. 10.1093/molbev/mst010 23329690 PMC3603318

[33] Guindon S, Dufayard JF, Lefort V, Anisimova M, Hordijk W, Gascuel O. New algorithms and methods to estimate maximum-likelihood phylogenies: assessing the performance of PhyML 3.0. Syst Biol. 2010; 59: 307–321. 10.1093/sysbio/syq010 20525638

[34] Lefort V, Longueville JE, Gascuel O. SMS: Smart Model Selection in PhyML. Mol Biol Evol. 2017; 34: 2422–2424. 10.1093/molbev/msx149 28472384 PMC5850602

[35] Trifinopoulos J, Nguyen LT, von Haeseler A, Minh BQ. W-IQ-TREE: a fast online phylogenetic tool for maximum likelihood analysis. Nucleic Acids Res. 2016; 44: W232–235. 10.1093/nar/gkw256 27084950 PMC4987875

[36] Wheeler TJ, Clements J, Finn RD. Skylign: a tool for creating informative, interactive logos representing sequence alignments and profile hidden Markov models. BMC Bioinformatics. 2014; 15: 7 10.1186/1471-2105-15-7 24410852 PMC3893531

[37] Akaike H. A new look at the statistical model identification. IEEE Trans Automatic Control. 1974; 19: 716–723.

[38] Darriba D, Taboada GL, Doallo R, Posada D. ProtTest 3: fast selection of best-fit models of protein evolution. Bioinformatics 2011; 27: 1164–1165. 10.1093/bioinformatics/btr088 21335321 PMC5215816

[39] Battistuzzi FU, Feijao A, Hedges AB. A genomic timescale of prokaryote evolution: insights into the origin of methanogenesis, phototrophy, and the colonization of land. BMC Evol Biol. 2004; 4: 44 10.1186/1471-2148-4-44 15535883 PMC533871

[40] Tomitani A, Knoll AH, Cavanaugh CM, Ohno T. The evolutionary diversification of cyanobacteria: molecular-phylogenetic and paleontological perspectives. Proc Natl Acad Sci USA. 2006; 103: 5442–5447. 10.1073/pnas.0600999103 16569695 PMC1459374

[41] Page RDM. TreeView: an application to display phylogenetic trees on personal computers. Comput Appl Biosci. 1996; 12: 357–358. 8902363 10.1093/bioinformatics/12.4.357

[42] Castresana J. Selection of conserved blocks from multiple alignments for their use in phylogenetic analysis. Mol Biol Evol. 2000; 17: 540–552. 10.1093/oxfordjournals.molbev.a026334 10742046

[43] Chen K, Durand D, Farach-Colton M. NOTUNG: a program for dating gene duplications and optimizing gene family trees. J Comput Biol. 2000; 7: 429–447. 10.1089/106652700750050871 11108472

[44] Magallón S, Hilu KW, Quandt D. Land plant evolutionary timeline: gene effects are secondary to fossil constraints in relaxed clock estimation of age and substitution rates. Am J Bot. 2013; 100:556–573. 10.3732/ajb.1200416 23445823

[45] Herron MD, Hackett JD, Aylward FO, Michod RE. Triassic origin and early radiation of multicellular volvocine algae. Proc Natl Acad Sci USA. 2009; 106: 3254–3258. 10.1073/pnas.0811205106 19223580 PMC2651347

[46] Parfrey LW, Lahr DJ, Knoll AH, Katz LA. Estimating the timing of early eukaryotic diversification with multigene molecular clocks. Proc Natl Acad Sci USA. 2011; 108: 13624–13629. 10.1073/pnas.1110633108 21810989 PMC3158185

[47] Hu B, Jin J, Guo AY, Zhang H, Luo J, Gao G. GSDS 2.0: an upgraded gene feature visualization server. Bioinformatics. 2015; 31: 1296–1297. 10.1093/bioinformatics/btu817 25504850 PMC4393523

[48] Neuwald AF, Aravind L, Spouge JL, Koonin EV. AAA+: A class of chaperone-like ATPases associated with the assembly, operation, and disassembly of protein complexes. Genome Res. 1999; 9: 27–43. 9927482

[49] Leipe DD, Koonin EV, Aravind L. Evolution and classification of P-loop kinases and related proteins. J Mol Biol. 2003; 333: 781–815. 14568537 10.1016/j.jmb.2003.08.040

[50] Osugi A, Sakakibara H. Q&A: How do plants respond to cytokinins and what is their importance? BMC Biol. 2015; 13: 102 10.1186/s12915-015-0214-5 26614311 PMC4662812

[51] Wang C, Liu Y, Li SS, Han GZ. Insights into the origin and evolution of the plant hormone signaling machinery. Plant Physiol. 2015; 167: 872–886. 10.1104/pp.114.247403 25560880 PMC4348752

[52] Maddison DR, Schulz KS eds. The Tree of Life Web Project. 2007. http://tolweb.org.

[53] Qiu YL, Li L, Wang B, Chen Z, Knoop V, Groth-Malonek M, et al The deepest divergences in land plants inferred from phylogenomic evidence. Proc Natl Acad Sci USA. 2006; 103: 15511–15516. 10.1073/pnas.0603335103 17030812 PMC1622854

[54] Hug LA, Baker BJ, Anantharaman K, Brown CT, Probst AJ, Castelle CJ, et al A new view of the tree of life. Nat Microbiol. 2016; 1: 16048 10.1038/nmicrobiol.2016.48 27572647

[55] Popper ZA, Michel G, Herve C, Domozych DS, Willats WG, Tuohy MG, et al Evolution and diversity of plant cell walls: from algae to flowering plants. Annu Rev Plant Biol. 2011; 62: 567–590. 10.1146/annurev-arplant-042110-103809 21351878

[56] Derelle R, Torruella G, Klimeš V, Brinkmann H, Kim E, Vlčcek Č, et al Bacterial proteins pinpoint a single eukaryotic root. Proc Natl Acad Sci USA. 2015; 112: E693–E699. 10.1073/pnas.1420657112 25646484 PMC4343179

[57] Finn RD, Mistry J, Schuster-Bockler B, Griffiths-Jones S, Hollich V, Lassmann T, et al Pfam: clans, web tools and services. Nucleic Acids Res. 2006; 34: D247–251. 10.1093/nar/gkj149 16381856 PMC1347511

[58] Punta M, Coggill PC, Eberhardt RY, Mistry J, Tate J, Boursnell C, et al The Pfam protein families database. Nucleic Acids Res. 2012; 40: D290–301. 10.1093/nar/gkr1065 22127870 PMC3245129

[59] Fraser CM, Gocayne JD, White O, Adams MD, Clayton RA, Fleischmann RD, et al The minimal gene complement of *Mycoplasma genitalium*. Science. 1995; 270: 397–403. 7569993 10.1126/science.270.5235.397

[60] Jameson P Cytokinins and auxins in plant-pathogen interactions—An overview. Plant Growth Regulation. 2000; 32: 369–380.

[61] Eichinger L, Pachebat JA, Glockner G, Rajandream MA, Sucgang R, Berriman M, et al The genome of the social amoeba *Dictyostelium discoideum*. Nature. 2005; 435: 43–57. 10.1038/nature03481 15875012 PMC1352341

[62] Moreau H, Piganeau G, Desdevises Y, Cooke R, Derelle E, Grimsley N. Marine prasinovirus genomes show low evolutionary divergence and acquisition of protein metabolism genes by horizontal gene transfer. J Virol. 2010; 84: 12555–12563. 10.1128/JVI.01123-10 20861243 PMC3004350

[63] Finke JF, Winget DM, Chan AM, Suttle CA. Variation in the genetic repertoire of viruses infecting *Micromonas pusilla* reflects horizontal gene transfer and links to their environmental distribution. Viruses. 2017; 9: 116. 10.3390/v9050116 PMC5454428 28534829

[64] Keeling PJ, Palmer JD. Horizontal gene transfer in eukaryotic evolution. Nat Rev Genet. 2008; 9: 605–618. 10.1038/nrg2386 18591983

[65] Wickett NJ, Mirarab S, Nguyen N, Warnow T, Carpenter E, Matasci N, et al Phylotranscriptomic analysis of the origin and early diversification of land plants. Proc Natl Acad Sci USA. 2014; 111: E4859–E4868. 10.1073/pnas.1323926111 25355905 PMC4234587

[66] Puttick MN, Morris JL, Williams TA, Cox CJ, Edwards D, Kenrick P, et al The interrelationships of land plants and the nature of the ancestral embryophyte. Curr Biol. 2018; 28: 733–745. 10.1016/j.cub.2018.01.063 29456145

[67] Pils B, Heyl A. Unraveling the evolution of cytokinin signaling. Plant Physiol. 2009; 151: 782–791. 10.1104/pp.109.139188 19675156 PMC2754637

[68] Morris JL, Puttick MN, Clark JW, Edwards D, Kenrick P, Pressel S, et al The timescale of early land plant evolution. Proc Natl Acad Sci USA. 2017; 115: E2274–E2283. 10.1073/pnas.1719588115 PMC5877938 29463716

[69] Clark JW, Donoghue PCJ. Constraining the timing of whole genome duplication in plant evolutionary history. Proc R Soc B. 2017; 284: 20170912 10.1098/rspb.2017.0912 28679730 PMC5524505

[70] Nystedt B, Street NR, Wetterbom A, Zuccolo A, Lin YC, Scofield DG, et al The Norway spruce genome sequence and conifer genome evolution. Nature. 2013; 497: 579–584. 10.1038/nature12211 23698360

[71] Stearns SC, Hoekstra RF. Evolution, an introduction. New York: Oxford University Press; 2005.

[72] Jeffares DC, Penkett CJ, Bahler J. Rapidly regulated genes are intron poor. Trends Genet. 2008; 24: 375–378. 10.1016/j.tig.2008.05.006 18586348

[73] Takei K, Ueda N, Aoki K, Kuromori T, Hirayama T, Shinozaki K, et al *AtIPT3* is a key determinant of nitrate-dependent cytokinin biosynthesis in *Arabidopsis*. Plant Cell Physiol. 2004; 45: 1053–1062. 10.1093/pcp/pch119 15356331

[74] Gajdošová S, Spíchal L, Kamínek M, Hoyerová K, Novák O, Dobrev PI, et al Distribution, biological activities, metabolism, and the conceivable function of *cis*-zeatin-type cytokinins in plants. J Exp Bot. 2011; 62: 2827–2840. 10.1093/jxb/erq457 21282330

[75] Kudo T, Makita N, Kojima M, Tokunaga H, Sakakibara H. Cytokinin activity of cis-zeatin and phenotypic alterations induced by overexpression of putative cis-Zeatin-*O*-glucosyltransferase in rice. Plant Physiol. 2012; 160: 319–331. 10.1104/pp.112.196733 22811434 PMC3440209

