# Supplementary material for: Tangled history of a multigene family: The evolution of ISOPENTENYLTRANSFERASE genes
Source: PLoS One. 2018 Aug 2;13(8):e0201198. doi: 10.1371/journal.pone.0201198 (PMC6071968; doi:10.1371/journal.pone.0201198)
Supplement: S1 Fig — Domains are shown as green boxes. Coordinates to the Pfam HMM profiles are shown below the boxes. (PDF) [file pone.0201198.s001.pdf]

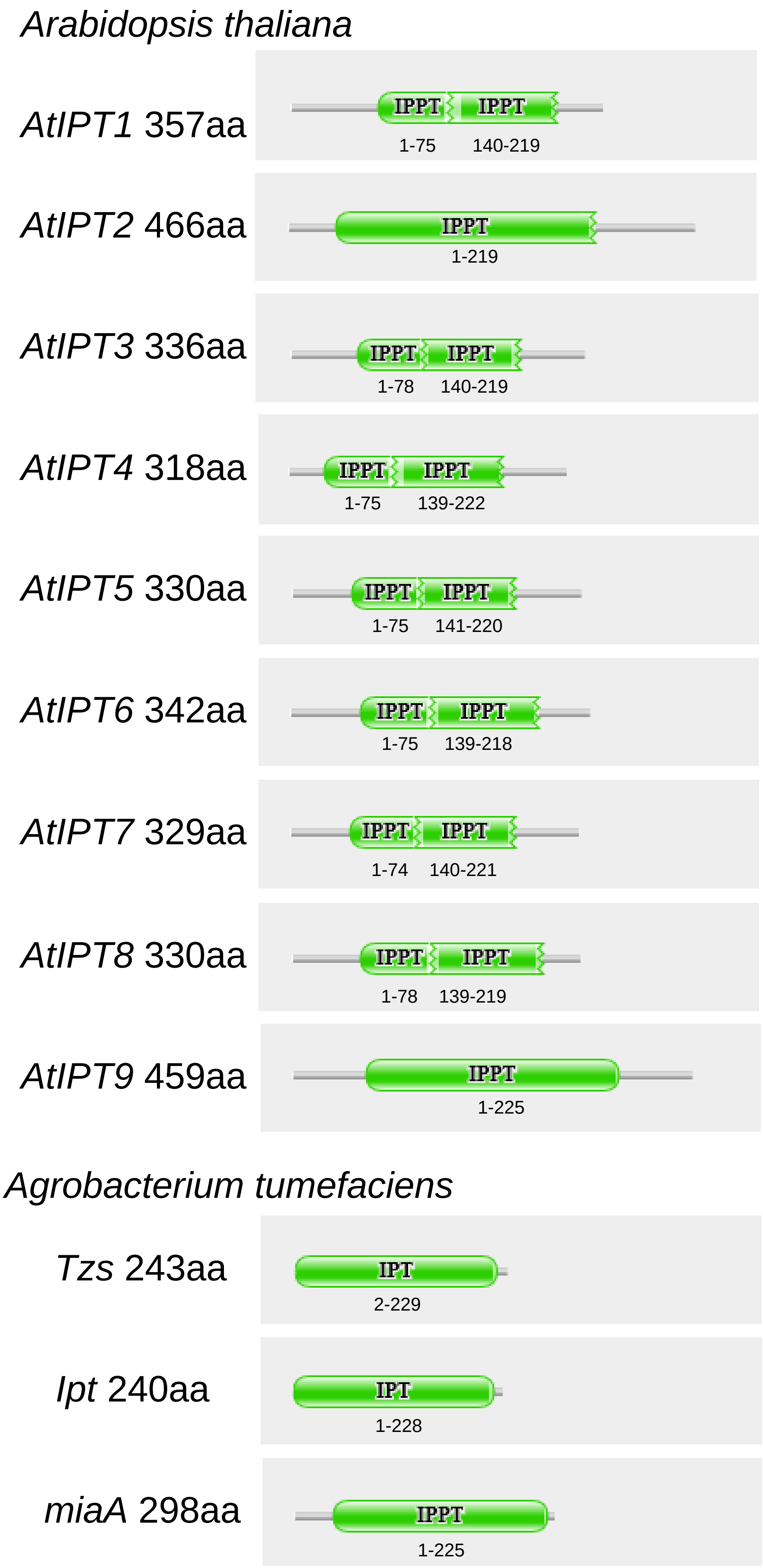

S1 Fig. Domain structure of *ISOPENTENYLTRANSFERASE*s in *Arabidopsis thaliana* and *Tzs*, *Ipt*, and *miaA* genes in *Agrobacterium tumefaciens*. Domains are shown as green boxes. Coordinates to the Pfam HMM profiles are shown below the boxes.
