# Supplementary material for: Tangled history of a multigene family: The evolution of ISOPENTENYLTRANSFERASE genes
Source: PLoS One. 2018 Aug 2;13(8):e0201198. doi: 10.1371/journal.pone.0201198 (PMC6071968; doi:10.1371/journal.pone.0201198)
Supplement: S2 Fig — The tree was calculated by pHMM-tree. IPTPfam and IPPTPfam families appeared as sister clades (arrow). (PDF) [file pone.0201198.s002.pdf]

S2 Fig.

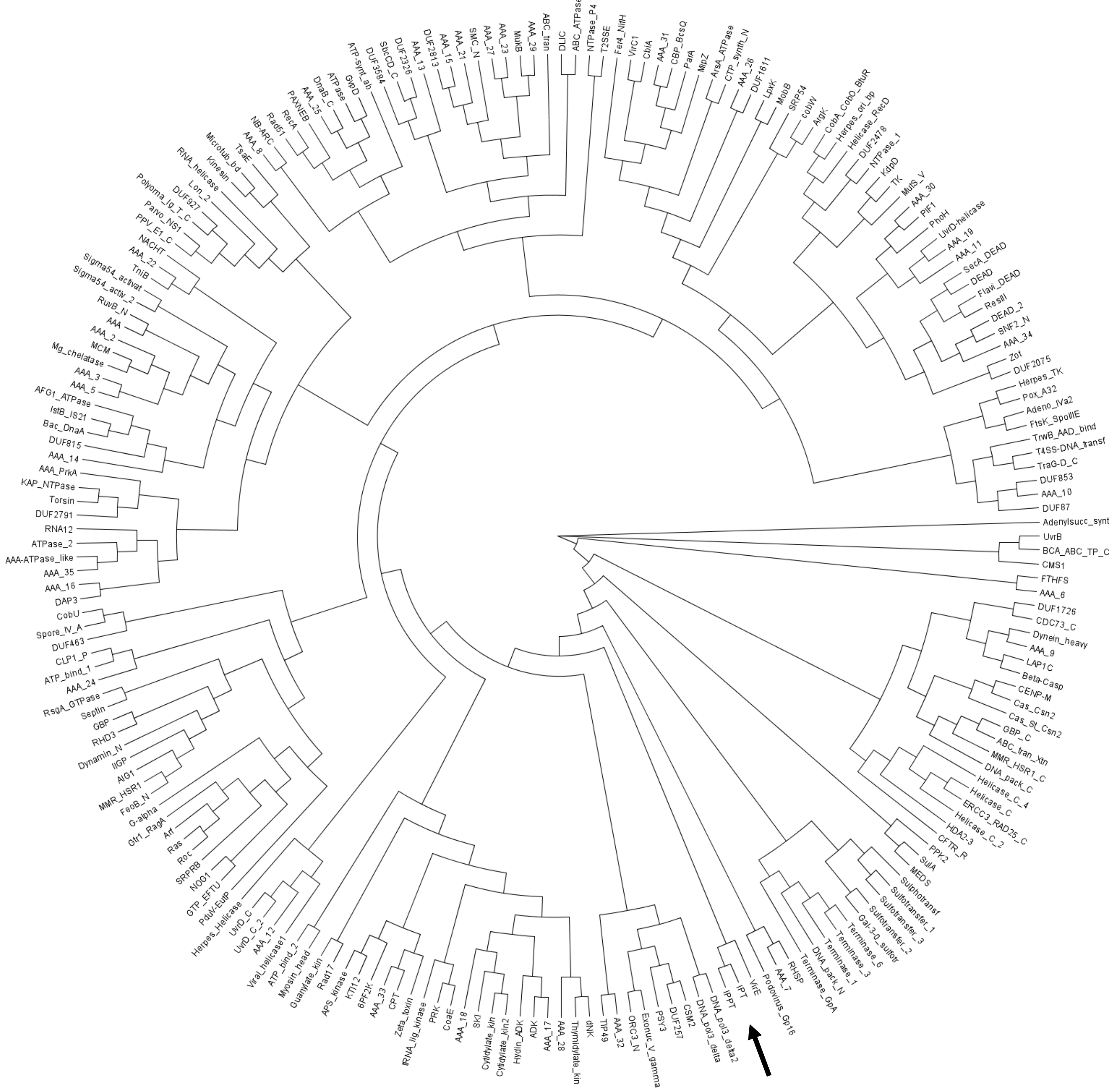

S2 Fig. Neighbor-joining tree of HMM profiles of P-loop NTPases in the Pfam database. The tree was calculated by pHMM-tree. IPT^{Pfam} and IPPT^{Pfam} families appeared as sister clades (arrow).

0.01
