# Supplementary material for: Tangled history of a multigene family: The evolution of ISOPENTENYLTRANSFERASE genes
Source: PLoS One. 2018 Aug 2;13(8):e0201198. doi: 10.1371/journal.pone.0201198 (PMC6071968; doi:10.1371/journal.pone.0201198)
Supplement: S4 Fig — Species retaining the IPPTPfam domain gene are shown in black, for species with both IPPTPfam and IPTPfam domain genes in orange, and for species lacking IPPTPfam and IPTPfam domain genes in grey. (PDF) [file pone.0201198.s004.pdf]

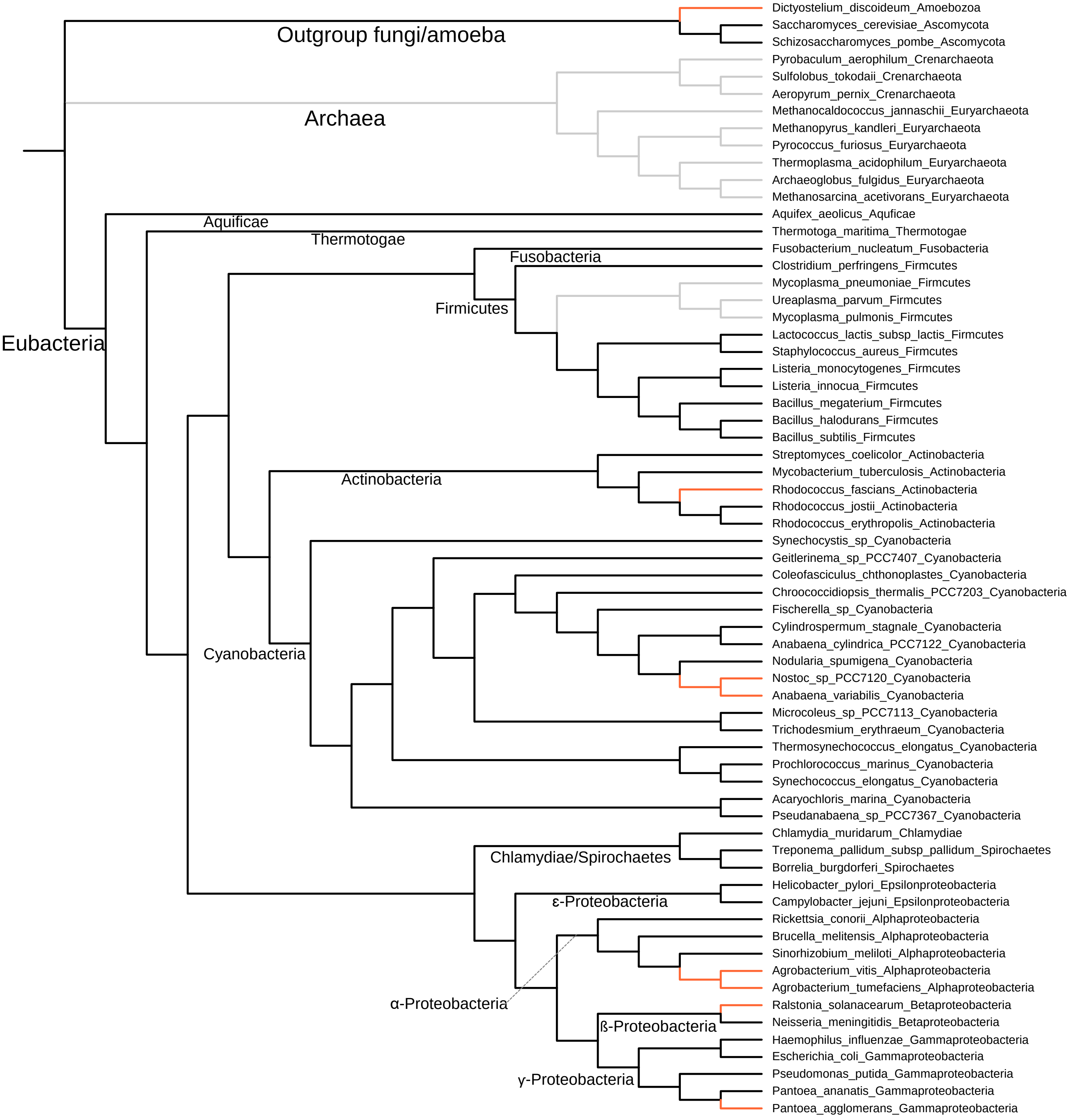

S4 Fig. Species tree of bacteria. Species retaining the IPPT^{Pfam} domain gene are shown in black, for species with both IPPT^{Pfam} and IPT^{Pfam} domain genes in orange, and for species lacking IPPT^{Pfam} and IPT^{Pfam} domain genes in grey.
