# Supplementary material for: Tangled history of a multigene family: The evolution of ISOPENTENYLTRANSFERASE genes
Source: PLoS One. 2018 Aug 2;13(8):e0201198. doi: 10.1371/journal.pone.0201198 (PMC6071968; doi:10.1371/journal.pone.0201198)
Supplement: S5 Fig — IPTPfam.hmm and the original IPPTPfam.hmm (1247 seed seq) were retrieved from Pfam v.31.0. IPPTPfam.hmm (103 seed seq) built with 103 representative out of 1247 seed sequences. Logos build from 1247 sequences in the original seed alignment and the 103 representative sequences were very similar. New HMM profile with extended N-terminus (IPPTPfam_N40.hmm) built in this study. N40: additional N-terminus region in IPPTPfam_N40.hmm. (PDF) [file pone.0201198.s005.pdf]

S5 Fig.

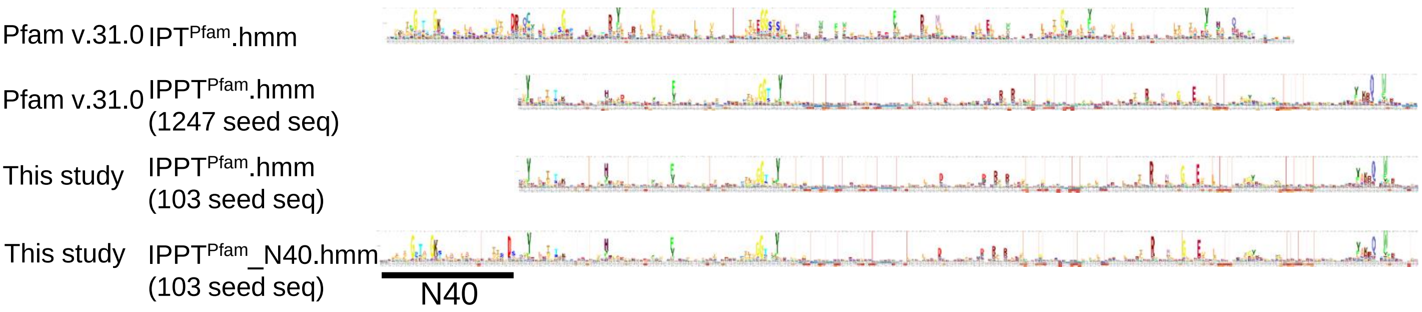

S5 Fig. Comparison of HMM logos between the original HMM registered in Pfam v.31.0 and the expanded HMM build in this study. IPT^{Pfam}.hmm and the original IPPT^{Pfam}.hmm (1247 seed seq) were retrieved from Pfam v.31.0. IPPT^{Pfam}.hmm (103 seed seq) built with 103 representative out of 1247 seed sequences. Logos build from 1247 sequences in the original seed alignment and the 103 representative sequences were very similar. New HMM profile with extended N-terminus (IPPT^{Pfam}_N40.hmm) built in this study. N40: additional N-terminus region in IPPT^{Pfam}_N40.hmm.
