# Supplementary material for: Tangled history of a multigene family: The evolution of ISOPENTENYLTRANSFERASE genes
Source: PLoS One. 2018 Aug 2;13(8):e0201198. doi: 10.1371/journal.pone.0201198 (PMC6071968; doi:10.1371/journal.pone.0201198)
Supplement: S6 Fig — Plant sequences in IPTPfam family of Pfam indicated by red arrows, and those shown in the IPPTPfam domain clade but not in the IPTPfam clade (αLRT SH-like = 1). (PDF) [file pone.0201198.s006.pdf]

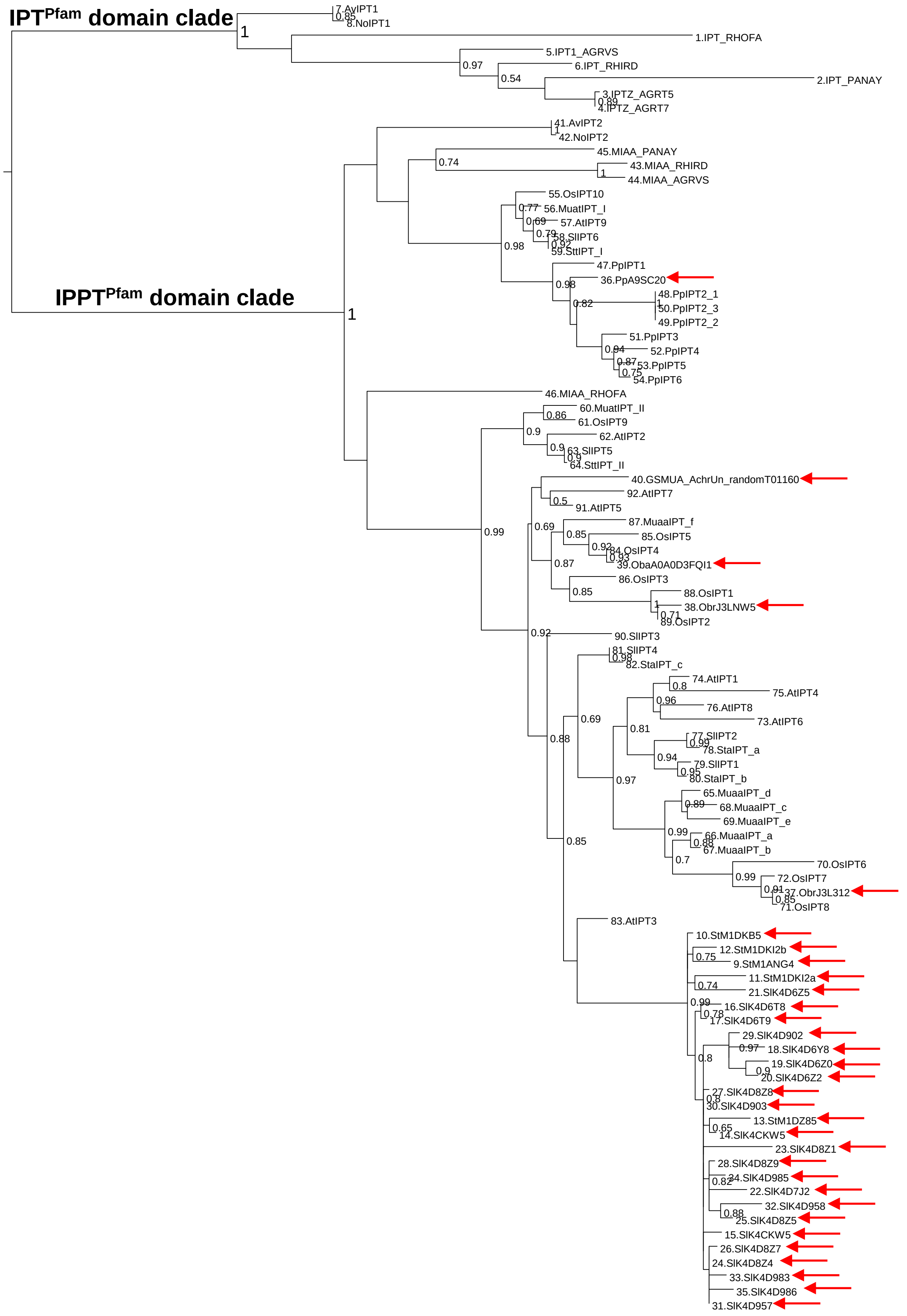

S6 Fig. ML tree calculated by PhyML including plant sequences registered in the IPT^{Pfam} family of Pfam database. Plant sequences in IPT^{Pfam} family of Pfam indicated by red arrows, and those shown in the IPPT^{Pfam} domain clade but not in the IPT^{Pfam} clade (αLRT SH-like = 1).
