# Supplementary material for: Tangled history of a multigene family: The evolution of ISOPENTENYLTRANSFERASE genes
Source: PLoS One. 2018 Aug 2;13(8):e0201198. doi: 10.1371/journal.pone.0201198 (PMC6071968; doi:10.1371/journal.pone.0201198)
Supplement: S7 Fig — The αLRT (left) and UFBT values (right) are shown along major branches. An asterisk indicates support values < 0.5 and < 50%. Thickened branches indicate support values > 0.9 and > 90%, medium-thick branches indicate > 0.7 and > 70%. The classification of the species is indicated by two characters at the end of the gene names; Ac: Actinobacteria, Al: α-Proteobacteria, Am: Amoebozoa, Aq: Aquficae, As: Ascomycota, Be: β-Proteobacteria, Ch: Chlamydiae, Cy: Cyanobacteria, Ep: ε-Proteobacteria, Fi: Firmicutes, Fu: Fusobacteria, Ga: γ-Proteobacteria, Sp: Spirochaetes, Th: Thermotogae. (PDF) [file pone.0201198.s007.pdf]

S7 Fig.

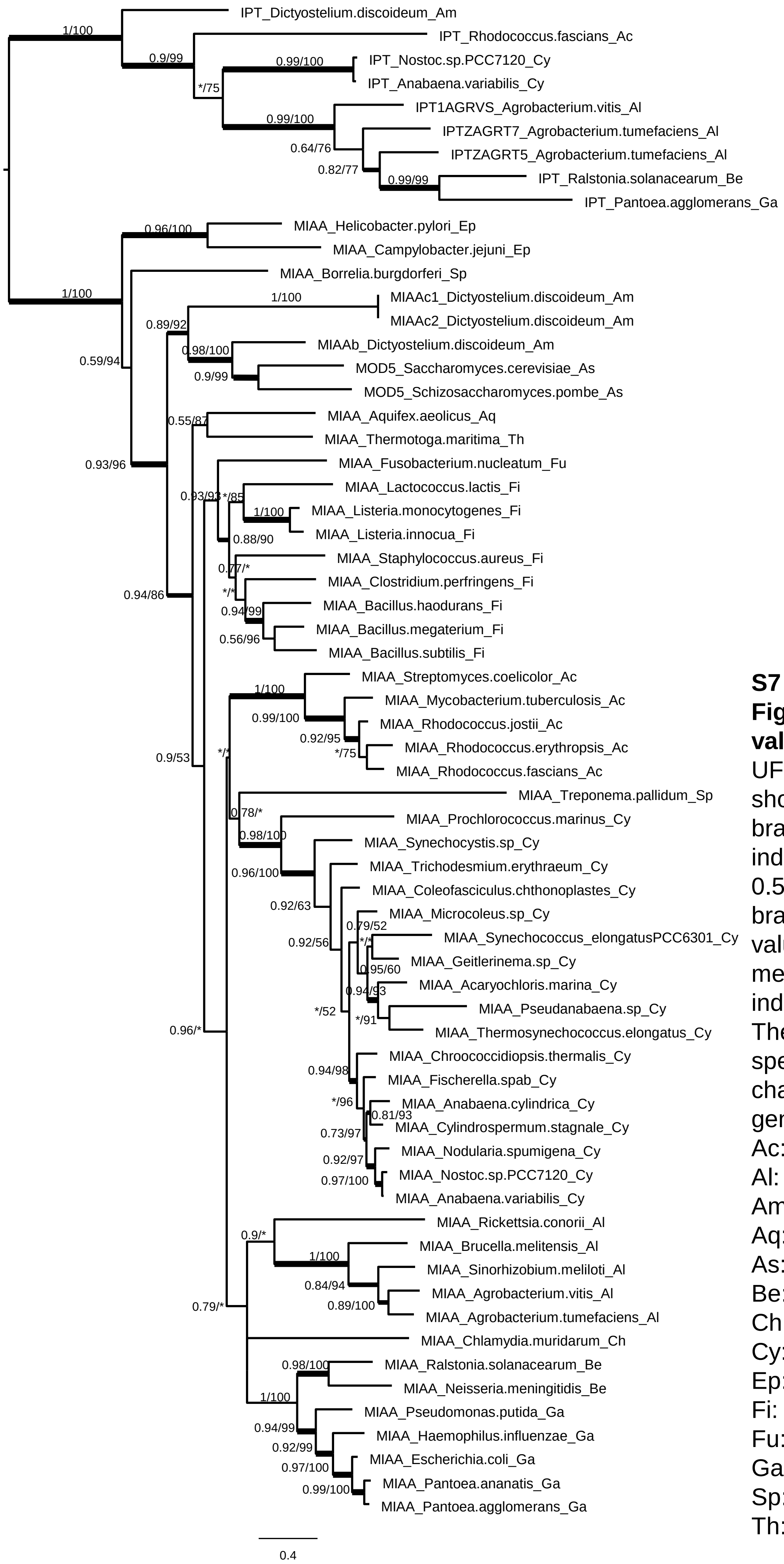

S7 Fig. ML tree shown in Fig. 2 with all support values. The αLRT (left) and UFBT values (right) are shown along major branches. An asterisk indicates support values < 0.5 and < 50%. Thickened branches indicate support values > 0.9 and > 90%, medium-thick branches indicate > 0.7 and > 70%. The classification of the species is indicated by two characters at the end of the gene names;
Ac: Actinobacteria,
Al: α-Proteobacteria,
Am: Amoebozoa,
Aq: Aquficae,
As: Ascomycota,
Be: β-Proteobacteria,
Ch: Chlamydiae,
Cy: Cyanobacteria,
Ep: ε-Proteobacteria,
Fi: Firmicutes,
Fu: Fusobacteria,
Ga: γ-Proteobacteria,
Sp: Spirochaetes,
Th: Thermotogae.
