# Supplementary material for: Tangled history of a multigene family: The evolution of ISOPENTENYLTRANSFERASE genes
Source: PLoS One. 2018 Aug 2;13(8):e0201198. doi: 10.1371/journal.pone.0201198 (PMC6071968; doi:10.1371/journal.pone.0201198)
Supplement: S8 Fig — IPTPfam domain genes were used as outgroup. The αLRT (left) and UFBT support values (right), are shown along the major branches. An asterisk indicates support values < 0.5 and < 50%. Thickened branches indicate support values > 0.9 and > 90%, medium-thick branches indicate > 0.7 and > 70%. (PDF) [file pone.0201198.s008.pdf]

S8 Fig.

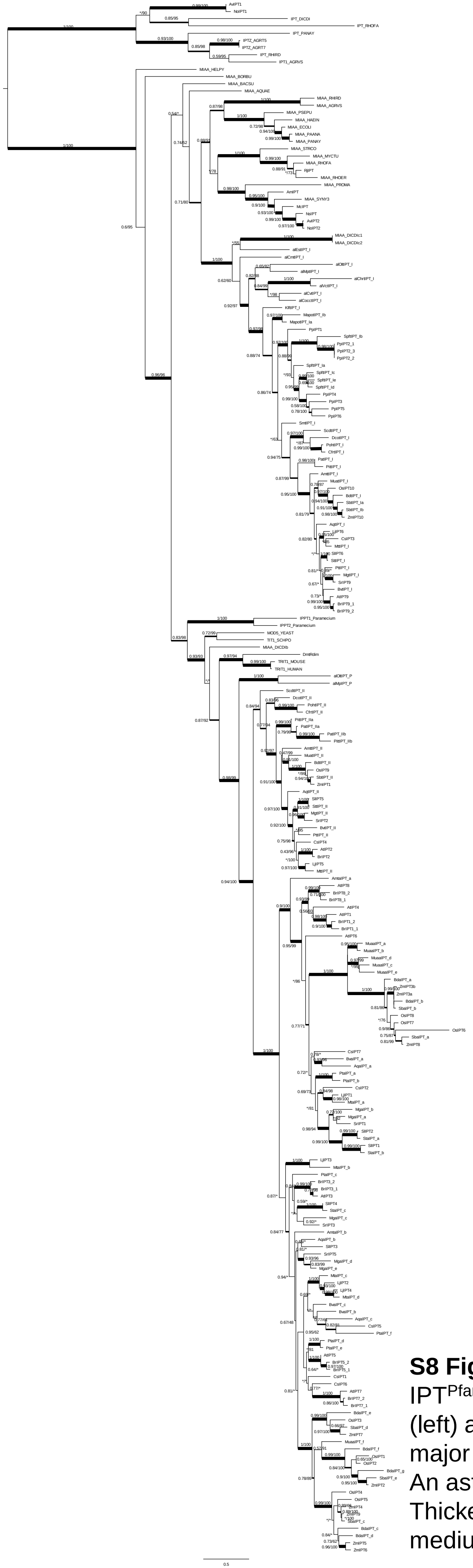

S8 Fig. ML tree shown in Fig. 3 with all support values.
IPT^{Pfam} domain genes were used as outgroup. The αLRT (left) and UFBT support values (right), are shown along the major branches.
An asterisk indicates support values < 0.5 and < 50%.
Thickened branches indicate support values > 0.9 and > 90%, medium-thick branches indicate > 0.7 and > 70%.
