# Supplementary figures and images for: Tangled history of a multigene family: The evolution of ISOPENTENYLTRANSFERASE genes
Source: PLoS One. 2018 Aug 2;13(8):e0201198. doi: 10.1371/journal.pone.0201198 (PMC6071968; doi:10.1371/journal.pone.0201198)

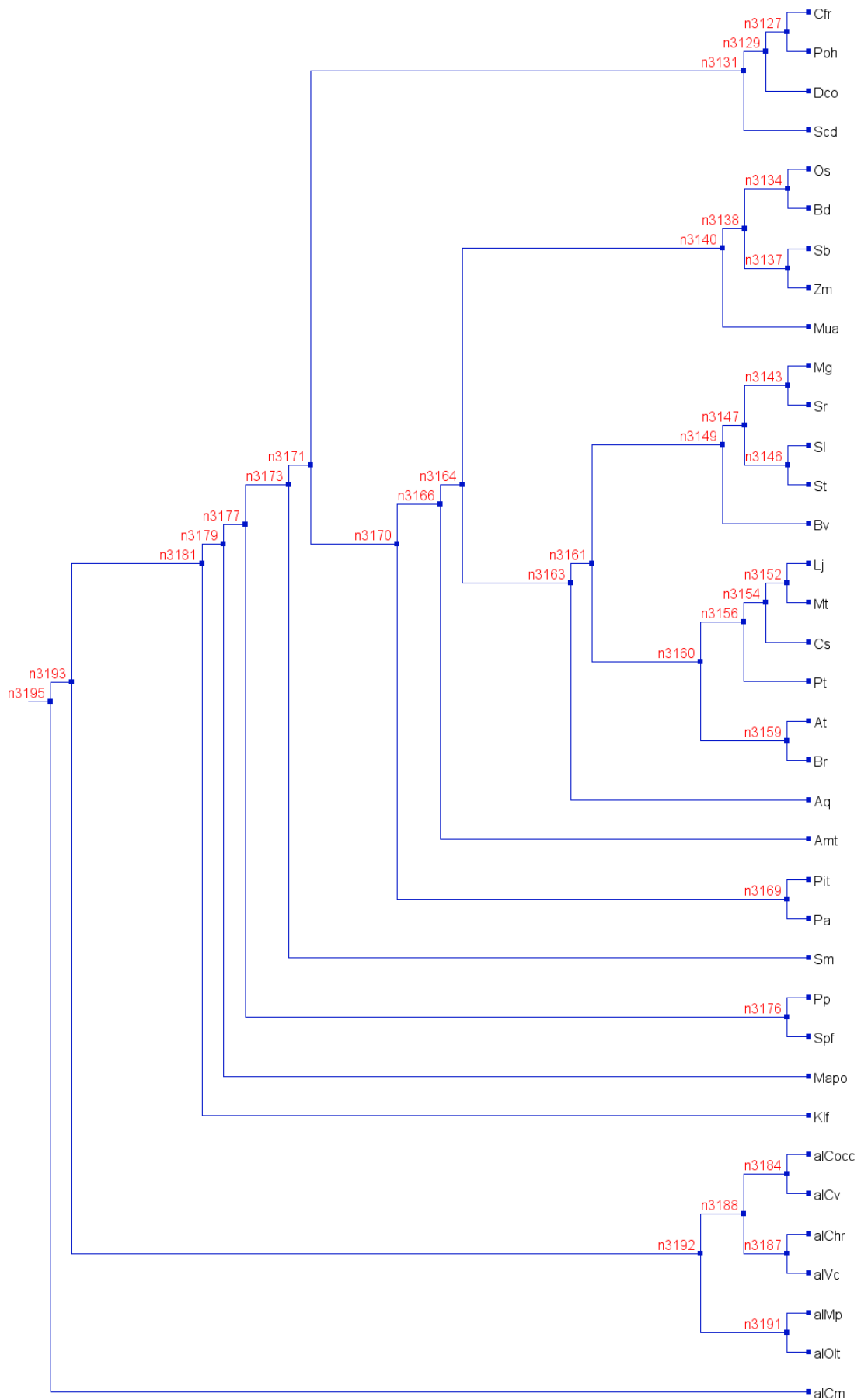

**S9 Fig. Species tree used for NOTUNG analyses of plant class I tRNA-*IPTs*.**

Supplement: S9 Fig — (PDF) [file pone.0201198.s009.pdf]

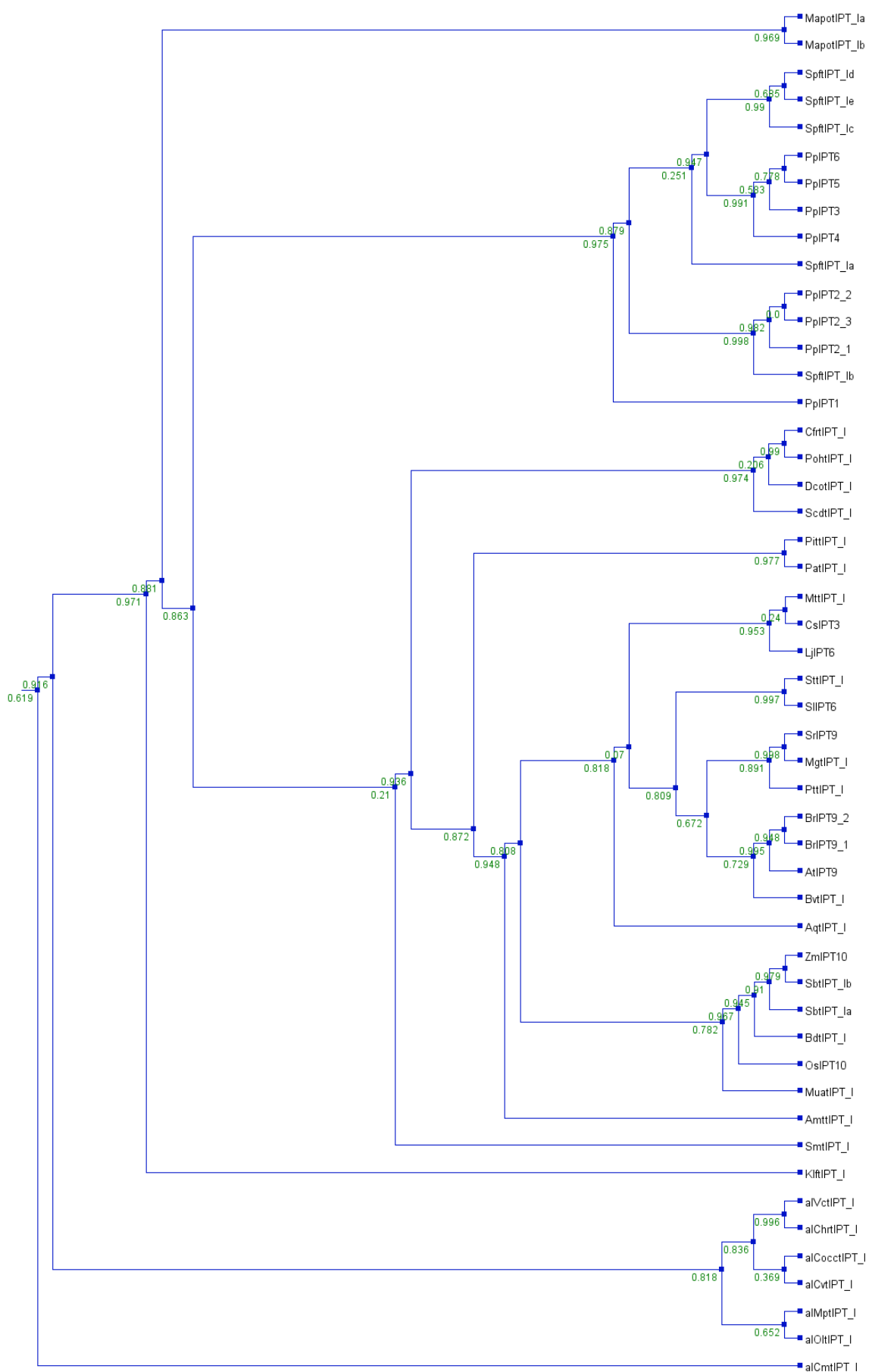

**S10 Fig. Gene tree used for NOTUNG analyses of plant class I tRNA-*IPTs*.**

Supplement: S10 Fig — (PDF) [file pone.0201198.s010.pdf]

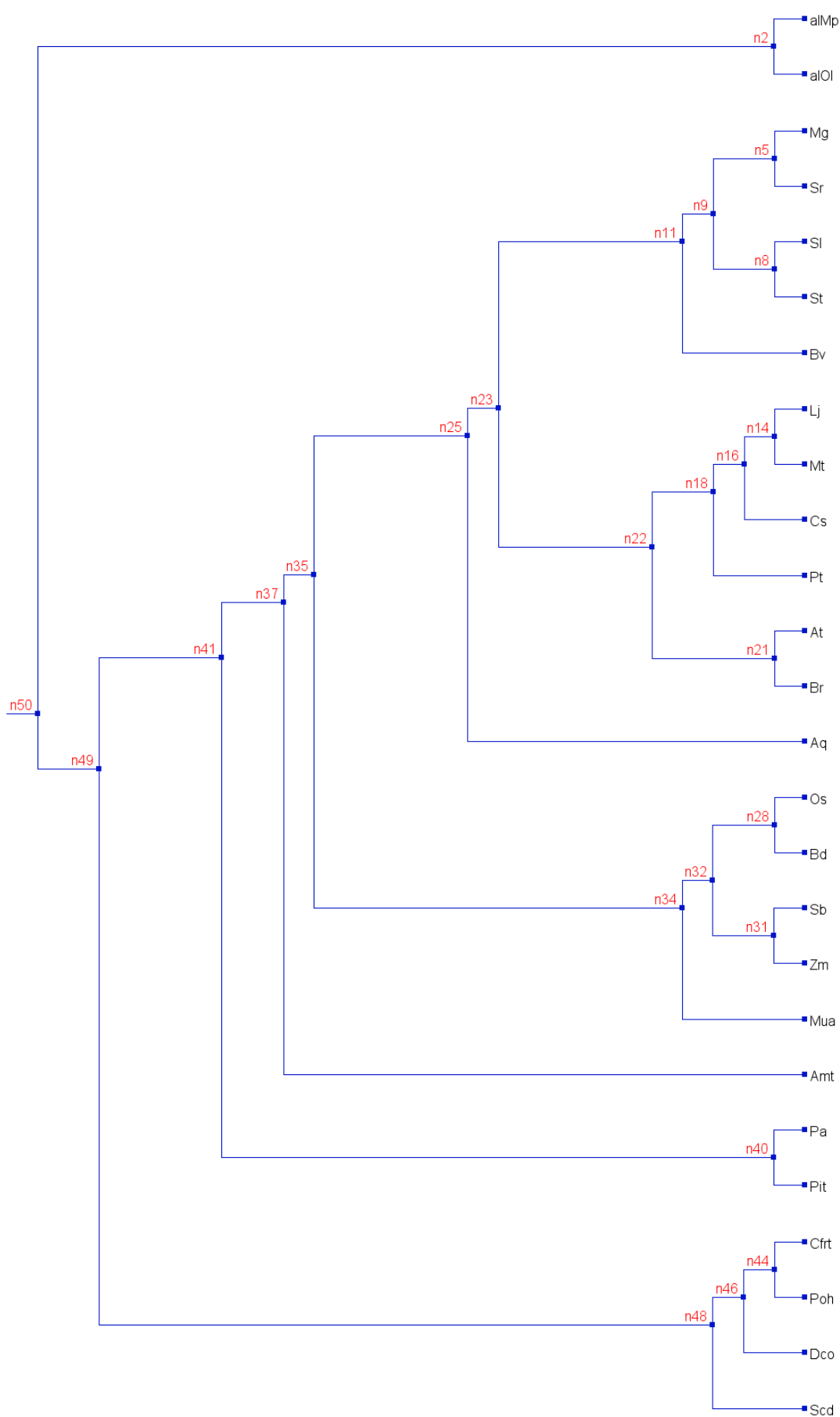

S12 Fig. Species tree used for NOTUNG analyses of plant class II tRNA-*IPTs*/AP-*IPTs*.

Supplement: S12 Fig — (PDF) [file pone.0201198.s012.pdf]

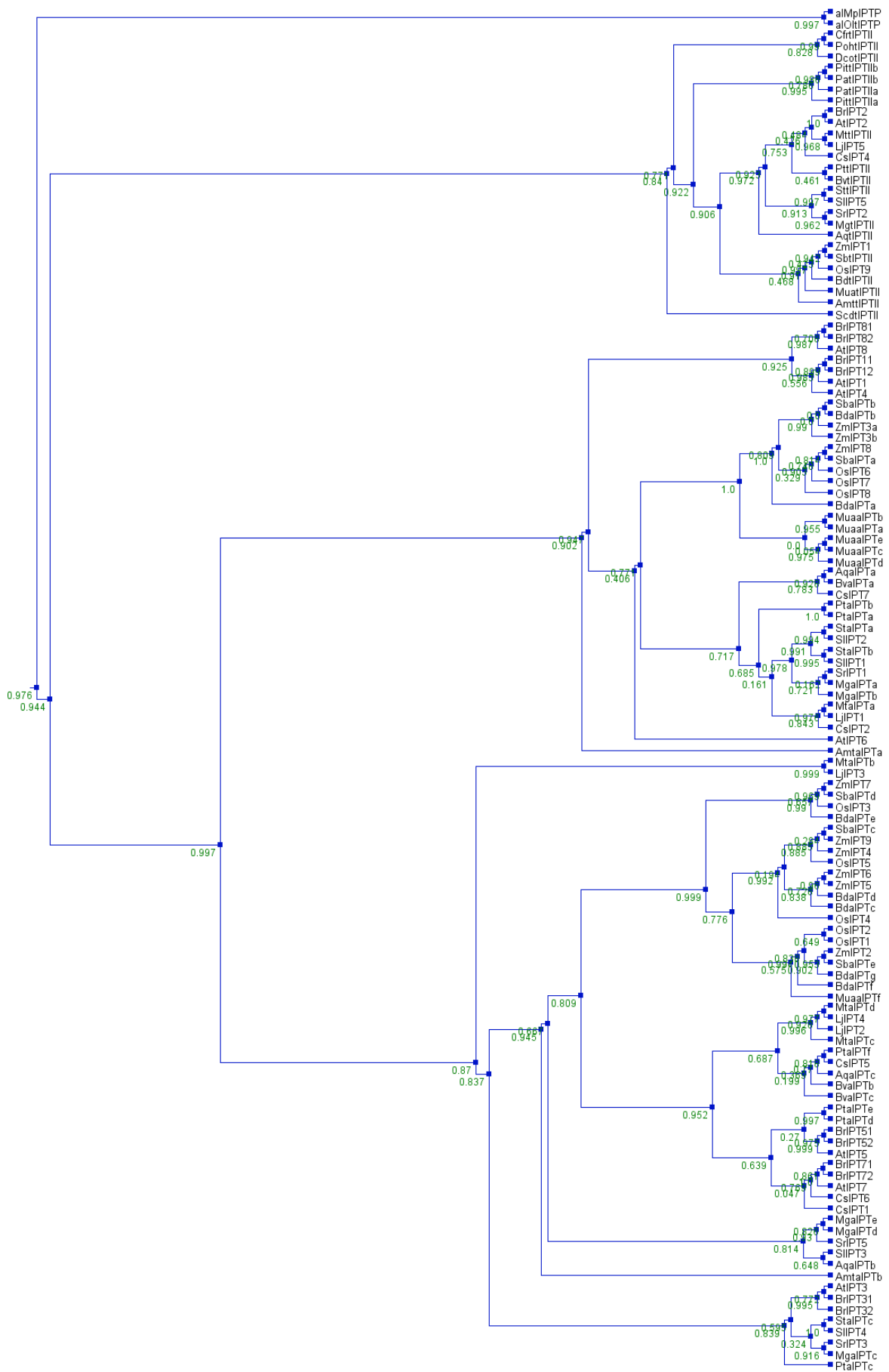

S13 Fig. Gene tree used for NOTUNG analyses of plant class II tRNA-IPTs/AP-IPTs.

Supplement: S13 Fig — (PDF) [file pone.0201198.s013.pdf]
