# Supplementary material for: Tangled history of a multigene family: The evolution of ISOPENTENYLTRANSFERASE genes
Source: PLoS One. 2018 Aug 2;13(8):e0201198. doi: 10.1371/journal.pone.0201198 (PMC6071968; doi:10.1371/journal.pone.0201198)
Supplement: S11 Fig — Weak edges highlighted yellow. Gene duplications marked by red ‘D’. (PDF) [file pone.0201198.s011.pdf]

S11 Fig.

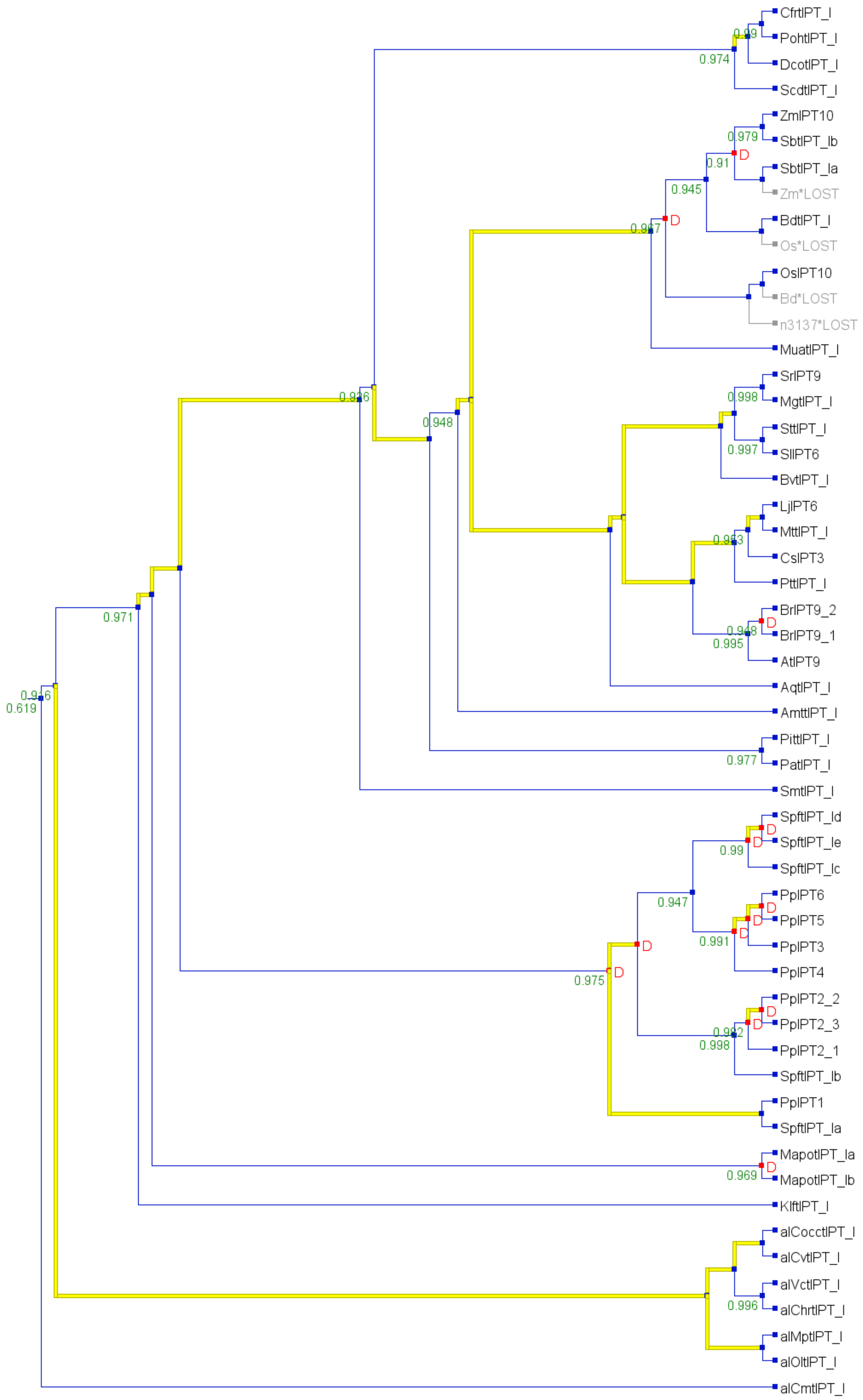

S11 Fig. NOTUNG DL analyses of plant class I tRNA-*IPT*s. Weak edges highlighted yellow. Gene duplications marked by red ‘D’.
