# Supplementary material for: Tangled history of a multigene family: The evolution of ISOPENTENYLTRANSFERASE genes
Source: PLoS One. 2018 Aug 2;13(8):e0201198. doi: 10.1371/journal.pone.0201198 (PMC6071968; doi:10.1371/journal.pone.0201198)
Supplement: S14 Fig — Weak edges highlighted yellow. Gene duplication marked by red ‘D’. (PDF) [file pone.0201198.s014.pdf]

S14 Fig.

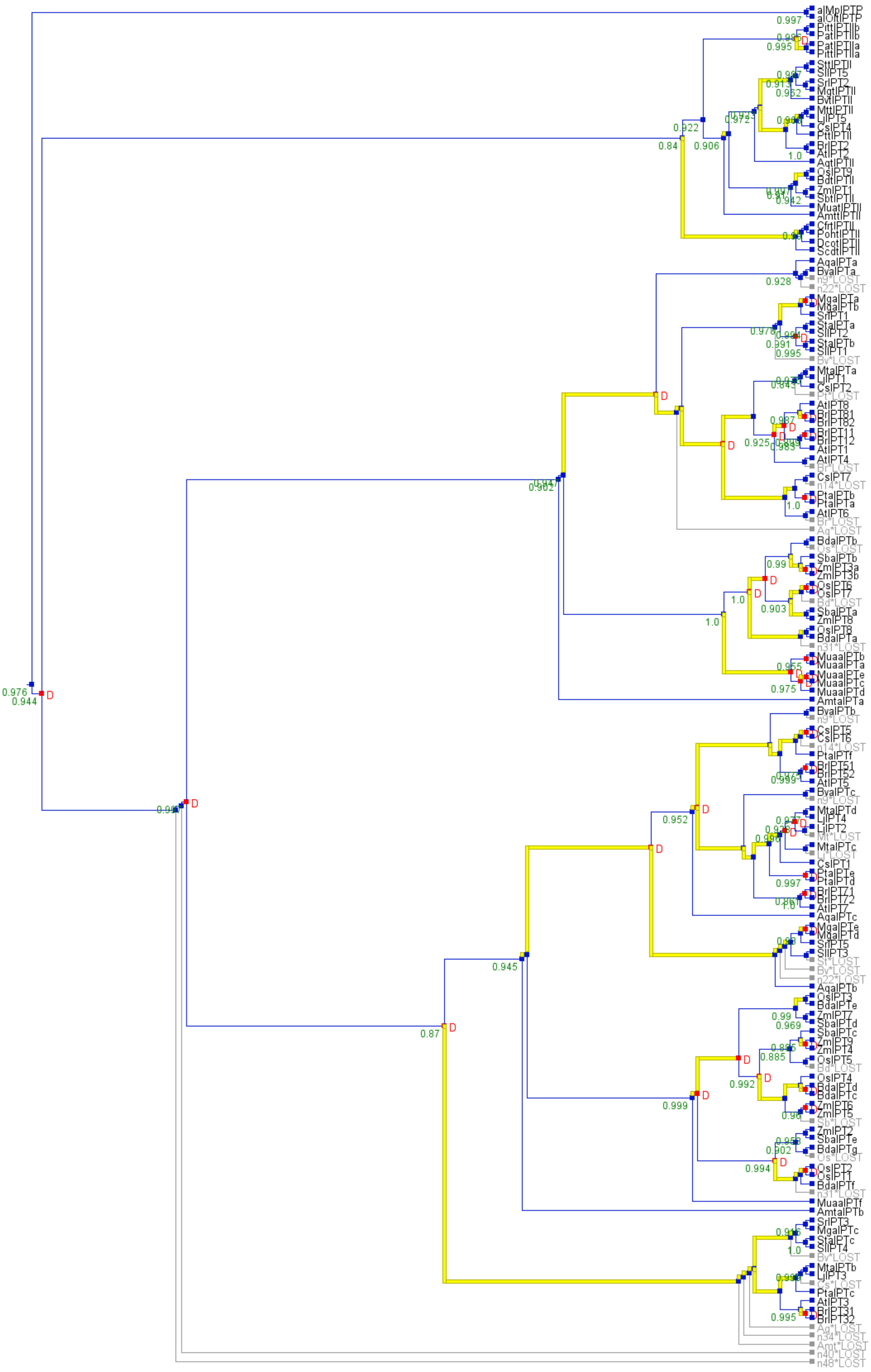

S14 Fig. NOTUNG DL analyses of plant class II tRNA-IPTs/AP-*IPTs*. Weak edges highlighted yellow. Gene duplication marked by red 'D'.
