# Supplementary material for: Tangled history of a multigene family: The evolution of ISOPENTENYLTRANSFERASE genes
Source: PLoS One. 2018 Aug 2;13(8):e0201198. doi: 10.1371/journal.pone.0201198 (PMC6071968; doi:10.1371/journal.pone.0201198)
Supplement: S15 Fig — The tree is a cladogram of the tree shown in Fig 3. Intron positions are shown as schematic illustrations. Asterisks indicate absence of introns in the gene. Genes without intron information are shown with ‘?’. Gene expressions are shown in square boxes: red indicates strong expression, orange indicates medium expression or expression without quantification, white indicates very weak or no expression in the tissues indicated (see also S3 Table). The response to external cytokinin treatments are indicated by upper or lower triangles: Upper triangles indicate the responses in the above ground parts of plants, and lower triangles indicate the responses in roots. Increase in gene expression is shown in yellow, no change in blue, and reduced expression in white (see also S4 Table). (PDF) [file pone.0201198.s015.pdf]

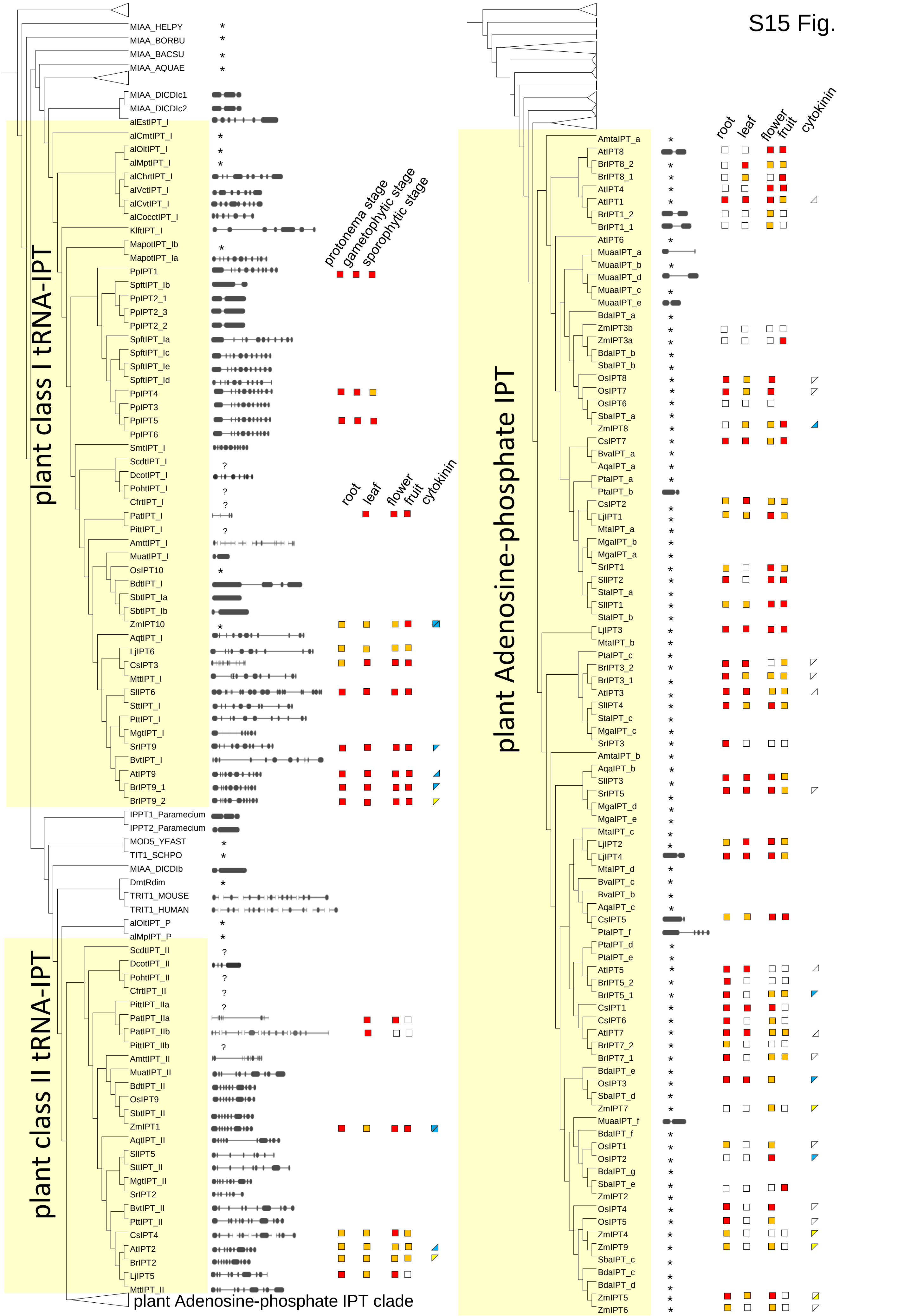

S15 Fig.
plant class I tRNA-IPT
plant class II tRNA-IPT
plant Adenosine-phosphate IPT
plant Adenosine-phosphate IPT clade
protonema stage
gametophytic stage
sporophytic stage
root
leaf
flower
fruit
cytokinin
MIAA_HELPY
MIAA_BORBU
MIAA_BACSU
MIAA_AQUAE
MIAA_DICDIc1
MIAA_DICDIc2
alEstIPT_I
alCmtIPT_I
alOltIPT_I
alMptIPT_I
alChrtIPT_I
alVctIPT_I
alCvtIPT_I
alCocctIPT_I
KlftIPT_I
MapotIPT_Ib
MapotIPT_Ia
PpIPT1
SpftIPT_Ib
PpIPT2_1
PpIPT2_3
PpIPT2_2
SpftIPT_Ia
SpftIPT_Ic
SpftIPT_Ie
SpftIPT_Id
PpIPT4
PpIPT3
PpIPT5
PpIPT6
SmtIPT_I
ScdtIPT_I
DcotIPT_I
PohtIPT_I
CfrtIPT_I
PatIPT_I
PittIPT_I
AmttIPT_I
MuatIPT_I
OsIPT10
BdtIPT_I
SbtIPT_Ia
SbtIPT_Ib
ZmIPT10
AqtIPT_I
LjIPT6
CsIPT3
MttIPT_I
SlIPT6
SttIPT_I
PttIPT_I
MgtIPT_I
SrIPT9
BvtIPT_I
AtIPT9
BrIPT9_1
BrIPT9_2
IPPT1_Paramecium
IPPT2_Paramecium
MOD5_YEAST
TIT1_SCHPO
MIAA_DICDIb
DmtRdim
TRIT1_MOUSE
TRIT1_HUMAN
alOltIPT_P
alMpIPT_P
ScdtIPT_II
DcotIPT_II
PohtIPT_II
CfrtIPT_II
PittIPT_IIa
PatIPT_IIa
PatIPT_IIb
PittIPT_IIb
AmttIPT_II
MuatIPT_II
BdtIPT_II
OsIPT9
SbtIPT_II
ZmIPT1
AqtIPT_II
SlIPT5
SttIPT_II
MgtIPT_II
SrIPT2
BvtIPT_II
PttIPT_II
CsIPT4
AtIPT2
BrIPT2
LjIPT5
MttIPT_II
AmtaIPT_a
AtIPT8
BrIPT8_2
BrIPT8_1
AtIPT4
AtIPT1
BrIPT1_2
BrIPT1_1
AtIPT6
MuaaIPT_a
MuaaIPT_b
MuaaIPT_d
MuaaIPT_c
MuaaIPT_e
BdaIPT_a
ZmIPT3b
ZmIPT3a
BdaIPT_b
SbaIPT_b
OsIPT8
OsIPT7
OsIPT6
SbaIPT_a
ZmIPT8
CsIPT7
BvaIPT_a
AqaIPT_a
PtaIPT_a
PtaIPT_b
CsIPT2
LjIPT1
MtaIPT_a
MgaIPT_b
MgaIPT_a
SrIPT1
SlIPT2
StaIPT_a
SlIPT1
StaIPT_b
LjIPT3
MtaIPT_b
PtaIPT_c
BrIPT3_2
BrIPT3_1
AtIPT3
SlIPT4
StaIPT_c
MgaIPT_c
SrIPT3
AmtaIPT_b
AqaIPT_b
SlIPT3
SrIPT5
MgaIPT_d
MgaIPT_e
MtaIPT_c
LjIPT2
LjIPT4
MtaIPT_d
BvaIPT_c
BvaIPT_b
AqaIPT_c
CsIPT5
PtaIPT_f
PtaIPT_d
PtaIPT_e
AtIPT5
BrIPT5_2
BrIPT5_1
CsIPT1
CsIPT6
AtIPT7
BrIPT7_2
BrIPT7_1
BdaIPT_e
OsIPT3
SbaIPT_d
ZmIPT7
MuaaIPT_f
BdaIPT_f
OsIPT1
OsIPT2
BdaIPT_g
SbaIPT_e
ZmIPT2
OsIPT4
OsIPT5
ZmIPT4
ZmIPT9
SbaIPT_c
BdaIPT_c
BdaIPT_d
ZmIPT5
ZmIPT6

S15 Fig. Summary of intron positions, expression patterns, cytokinin interaction of plant *ISOPENTENYLTRANSFERASEs* alongside the phylogenetic tree. The tree is a cladogram of the tree shown in Fig. 3. Intron positions are shown as schematic illustrations. Asterisks indicate absence of introns in the gene. Genes without intron information are shown with '?'. Gene expressions are shown in square boxes: red indicates strong expression, orange indicates medium expression or expression without quantification, white indicates very weak or no expression in the tissues indicated (see also S3 Table). The response to external cytokinin treatments are indicated by upper or lower triangles: Upper triangles indicate the responses in the above ground parts of plants, and lower triangles indicate the responses in roots. Increase in gene expression is shown in yellow, no change in blue, and reduced expression in white (see also S4 Table).
