# Supplementary material for: Tangled history of a multigene family: The evolution of ISOPENTENYLTRANSFERASE genes
Source: PLoS One. 2018 Aug 2;13(8):e0201198. doi: 10.1371/journal.pone.0201198 (PMC6071968; doi:10.1371/journal.pone.0201198)
Supplement: S1 Table — (PDF) [file pone.0201198.s016.pdf]

S1 Table. List of species used in this study and their classification, with the number of IPPT^{Pfam} and IPT^{Pfam} domain genes.

Classification										Num. of domain genes		Type of IPPT genes (see Table 1; Fig. 2)					
	Domain/ Kingdom	Phylum/Division/clade	Unranked/Class	Order	Family	Species	Abbreviation	Strain	Database	pIPPT	pIPT	A	B	C	D	E	F
Prokaryote	Archaea	Crenarchaeota	Thermoprotei	Thermoproteales	Thermoproteaceae	Pyrobaculum aerophilum			KEGG GENOME	0	0	0	0	0	0	0	0
Prokaryote	Archaea	Crenarchaeota	Thermoprotei	Desulfurococcales	Desulfurococcaceae	Aeropyrum pernix			KEGG GENOME	0	0	0	0	0	0	0	0
Prokaryote	Archaea	Crenarchaeota	Thermoprotei	Sulfolobales	Sulfolobaceae	Sulfolobus tokodaii			KEGG GENOME	0	0	0	0	0	0	0	0
Prokaryote	Archaea	Euryarchaeota	Methanopyri	Methanopyrales	Methanopyraceae	Methanopyrus kandleri			KEGG GENOME	0	0	0	0	0	0	0	0
Prokaryote	Archaea	Euryarchaeota	Thermococci	Thermococcales	Thermococcaceae	Pyrococcus furiosus			KEGG GENOME	0	0	0	0	0	0	0	0
Prokaryote	Archaea	Euryarchaeota	Methanococci	Methanococcales	Methanocaldococcaceae	Methanococcus jannaschii			KEGG GENOME	0	0	0	0	0	0	0	0
Prokaryote	Archaea	Euryarchaeota	Thermoplasmata	Thermoplasmatales	Thermoplasmataceae	Thermoplasma acidophilum			KEGG GENOME	0	0	0	0	0	0	0	0
Prokaryote	Archaea	Euryarchaeota	Archaeoglobi	Archaeoglobales	Archaeoglobaceae	Archaeoglobus fulgidus			KEGG GENOME	0	0	0	0	0	0	0	0
Prokaryote	Archaea	Euryarchaeota	Methanomicrobia	Methanosarcinales	Methanosarcinaceae	Methanosarcina acetivorans			KEGG GENOME	0	0	0	0	0	0	0	0
Prokaryote	Eubacteria	Aquficae	Aquificae	Aquificales	Aquificaceae	Aquifex aeolicus		VF5	KEGG GENOME	1	0	1	0	0	0	0	0
Prokaryote	Eubacteria	Thermotogae	Thermotogae	Thermotogales	Thermotogaceae	Thermotoga maritima		MSB8	KEGG GENOME	1	0	1	0	0	0	0	0
Prokaryote	Eubacteria	Firmcutes	Bacilli	Bacillales	Listeriaceae	Listeria innocua			KEGG GENOME	1	0	1	0	0	0	0	0
Prokaryote	Eubacteria	Firmcutes	Bacilli	Bacillales	Listeriaceae	Listeria monocytogenes		EGD-e	KEGG GENOME	1	0	1	0	0	0	0	0
Prokaryote	Eubacteria	Firmcutes	Bacilli	Bacillales	Bacillaceae	Bacillus halodurans			KEGG GENOME	1	0	1	0	0	0	0	0
Prokaryote	Eubacteria	Firmcutes	Bacilli	Bacillales	Bacillaceae	Bacillus megaterium		QM B1551	KEGG GENOME	1	0	1	0	0	0	0	0
Prokaryote	Eubacteria	Firmcutes	Bacilli	Bacillales	Bacillaceae	Bacillus subtilis			KEGG GENOME	1	0	1	0	0	0	0	0
Prokaryote	Eubacteria	Firmcutes	Bacilli	Bacillales	Staphylococcaceae	Staphylococcus aureus		N315	DOGAN	1	0	1	0	0	0	0	0
Prokaryote	Eubacteria	Firmcutes	Bacilli	Lactobacillales	Streptococcaceae	Lactococcus lactis subsp. Lactis		Il1403	KEGG GENOME	1	0	1	0	0	0	0	0
Prokaryote	Eubacteria	Firmcutes	Mollicutes	Mycoplasmatales	Mycoplasmataceae	Mycoplasma pneumoniae		M129	KEGG GENOME	0	0	0	0	0	0	0	0
Prokaryote	Eubacteria	Firmcutes	Mollicutes	Mycoplasmatales	Mycoplasmataceae	Mycoplasma pulmonis			KEGG GENOME	0	0	0	0	0	0	0	0
Prokaryote	Eubacteria	Firmcutes	Mollicutes	Mycoplasmatales	Mycoplasmataceae	Ureaplasma parvum		serovar 3 3V3F4	KEGG GENOME	0	0	0	0	0	0	0	0
Prokaryote	Eubacteria	Firmcutes	Clostridia	Clostridiales	Clostridiaceae	Clostridium perfringens		13	KEGG GENOME	1	0	1	0	0	0	0	0
Prokaryote	Eubacteria	Fusobacteria	Fusobacteria	Fusobacteriales	Fusobacteriaceae	Fusobacterium nucleatum subsp. nucleatum		ATCC 25586	KEGG GENOME	1	0	1	0	0	0	0	0
Prokaryote	Eubacteria	Cyanobacteria	Cyanophyceae	Chroococcales	Merismopediaceae	Synechocystis sp.			KEGG GENOME	1	0	1	0	0	0	0	0
Prokaryote	Eubacteria	Cyanobacteria	Cyanophyceae	Synechococcales	Acaryochloridaceae	Acaryochloris marina			KEGG GENOME	1	0	1	0	0	0	0	0
Prokaryote	Eubacteria	Cyanobacteria	Cyanophyceae	Synechococcales	Prochloraceae	Prochlorococcus marinus			KEGG GENOME	1	0	1	0	0	0	0	0
Prokaryote	Eubacteria	Cyanobacteria	Cyanophyceae	Synechococcales	Pseudanabaenaceae	Pseudanabaena sp.		PCC 7367	KEGG GENOME	1	0	1	0	0	0	0	0
Prokaryote	Eubacteria	Cyanobacteria	Cyanophyceae	Synechococcales	Synechococcaceae	Synechococcus elongatus		PCC 6301	KEGG GENOME	1	0	1	0	0	0	0	0
Prokaryote	Eubacteria	Cyanobacteria	Cyanophyceae	Synechococcales	Synechococcaceae	Thermosynechococcus elongatus			KEGG GENOME	1	0	1	0	0	0	0	0
Prokaryote	Eubacteria	Cyanobacteria	Oscillatoriophycideae	Oscillatoriales	Coleofasciculaceae	Coleofasciculus chthonoplastes			Genbank	1	0	1	0	0	0	0	0
Prokaryote	Eubacteria	Cyanobacteria	Cyanophyceae	Oscillatoriales	Coleofasciculaceae	Geitlerinema sp.		PCC 7407	KEGG GENOME	1	0	1	0	0	0	0	0
Prokaryote	Eubacteria	Cyanobacteria	Cyanophyceae	Oscillatoriales	Microcoleaceae	Microcoleus sp.		PCC 7113	KEGG GENOME	1	0	1	0	0	0	0	0
Prokaryote	Eubacteria	Cyanobacteria	Cyanophyceae	Oscillatoriales	Microcoleaceae	Trichodesmium erythraeum			KEGG GENOME	1	0	1	0	0	0	0	0
Prokaryote	Eubacteria	Cyanobacteria	Cyanophyceae	Chroococcidiopsidales	Chroococcidiopsidaceae	Chroococcidiopsis thermalis		PCC 7203	KEGG GENOME	1	0	1	0	0	0	0	0
Prokaryote	Eubacteria	Cyanobacteria	Cyanophyceae	Nostocales	Hapalosiphonaceae	Fischerella sp.		NIES-3754	KEGG GENOME	1	0	1	0	0	0	0	0
Prokaryote	Eubacteria	Cyanobacteria	Cyanophyceae	Nostocales	Aphanizomenonaceae	Nodularia spumigena			Genbank	1	0	1	0	0	0	0	0
Prokaryote	Eubacteria	Cyanobacteria	Cyanophyceae	Nostocales	Nostocaceae	Anabaena cylindrica		PCC 7122	KEGG GENOME	1	0	1	0	0	0	0	0
Prokaryote	Eubacteria	Cyanobacteria	Cyanophyceae	Nostocales	Nostocaceae	Anabaena variabilis			KEGG GENOME	1	1	1	0	0	0	0	0
Prokaryote	Eubacteria	Cyanobacteria	Cyanophyceae	Nostocales	Nostocaceae	Cylindrospermum stagnale			KEGG GENOME	1	0	1	0	0	0	0	0
Prokaryote	Eubacteria	Cyanobacteria	Cyanophyceae	Nostocales	Nostocaceae	Nostoc sp.		PCC 7120	UCSC Microbital Genome Browser	1	1	1	0	0	0	0	0
Prokaryote	Eubacteria	Actinobacteria	Actinobacteria	Actubintcubeae	Streptomycetaceae	Streptomyces coelicolor		A3(2)	KEGG GENOME	1	0	1	0	0	0	0	0
Prokaryote	Eubacteria	Actinobacteria	Actinobacteria	Actinomycetales	Mycobacteriaceae	Mycobacterium tuberculosis			KEGG GENOME	1	0	1	0	0	0	0	0
Prokaryote	Eubacteria	Actinobacteria	Actinobacteria	Actinomycetales	Nocardiaceae	Rhodococcus erythropsis			KEGG GENOME	1	0	1	0	0	0	0	0
Prokaryote	Eubacteria	Actinobacteria	Actinobacteria	Actinomycetales	Nocardiaceae	Rhodococcus fascians			Genbank	1	1	1	0	0	0	0	0
Prokaryote	Eubacteria	Actinobacteria	Actinobacteria	Actinomycetales	Nocardiaceae	Rhodococcus jostii			KEGG GENOME	1	0	1	0	0	0	0	0
Prokaryote	Eubacteria	Chlamydiae	Chlamydiae	Chlamydiales	Chlamydiaceae	Chlamydia muridarum		Nigg	KEGG GENOME	1	0	1	0	0	0	0	0
Prokaryote	Eubacteria	Spirochaetes	Spirochaetes	Spirochaetales	Spirochaetaceae	Borrelia burgdorferi		B31	Genbank	1	0	1	0	0	0	0	0
Prokaryote	Eubacteria	Spirochaetes	Spirochaetes	Spirochaetales	Spirochaetaceae	Borrelia burgdorferi			KEGG GENOME	1	0	1	0	0	0	0	0
Prokaryote	Eubacteria	Spirochaetes	Spirochaetes	Spirochaetales	Spirochaetaceae	Treponema pallidum subsp. pallidum		Nichols	KEGG GENOME	1	0	1	0	0	0	0	0
Prokaryote	Eubacteria	Epsilonproteobacteria	Epsilonproteobacteria	Campylobacterales	Helicobacteraceae	Helicobacter pylori		Rif1	KEGG GENOME	1	0	1	0	0	0	0	0
Prokaryote	Eubacteria	Epsilonproteobacteria	Epsilonproteobacteria	Campylobacterales	Campylobacteraceae	Campylobacter jejuni		RM1221	KEGG GENOME	1	0	1	0	0	0	0	0
Prokaryote	Eubacteria	Alphaproteobacteria	Alphaproteobacteria	Rhizobiales	Rhizobiaceae	Agrobacterium tumefaciens			KEGG GENOME	1	2	1	0	0	0	0	0
Prokaryote	Eubacteria	Alphaproteobacteria	Alphaproteobacteria	Rhizobiales	Rhizobiaceae	Agrobacterium vitis			KEGG GENOME	1	1	1	0	0	0	0	0
Prokaryote	Eubacteria	Alphaproteobacteria	Alphaproteobacteria	Rhizobiales	Rhizobiaceae	Sinorhizobium meliloti		1021	KEGG GENOME	1	0	1	0	0	0	0	0
Prokaryote	Eubacteria	Alphaproteobacteria	Alphaproteobacteria	Rickettsiales	Rickettsiaceae	Rickettsia conorii			KEGG GENOME	1	0	1	0	0	0	0	0
Prokaryote	Eubacteria	Alphaproteobacteria	Alphaproteobacteria	Rhizobiales	Brucellaceae	Brucella melitensis			KEGG GENOME	1	0	1	0	0	0	0	0
Prokaryote	Eubacteria	Betaproteobacteria	Betaproteobacteria	Burkholderiales	Burkholderiaceae	Ralstonia solanacearum		GMI1000	KEGG GENOME	1	1	1	0	0	0	0	0
Prokaryote	Eubacteria	Betaproteobacteria	Betaproteobacteria	Neisseriales	Neisseriaceae	Neisseria meningitidis		MC58	KEGG GENOME	1	0	1	0	0	0	0	0
Prokaryote	Eubacteria	Gammaproteobacteria	Gammaproteobacteria	Pasteurellales	Pasteurellaceae	Haemophilus influenzae Rd KW20		Rd KW20	KEGG GENOME	1	0	1	0	0	0	0	0
Prokaryote	Eubacteria	Gammaproteobacteria	Gammaproteobacteria	Pseudomonadales	Pseudomonadaceae	Pseudomonas putida			KEGG GENOME	1	0	1	0	0	0	0	0
Prokaryote	Eubacteria	Gammaproteobacteria	Gammaproteobacteria	Enterobacteriales	Enterobacteriaceae	Escherichia coli			KEGG GENOME	1	0	1	0	0	0	0	0
Prokaryote	Eubacteria	Gammaproteobacteria	Gammaproteobacteria	Enterobacteriales	Enterobacteriaceae	Pantoea ananatis			KEGG GENOME	1	0	1	0	0	0	0	0
Prokaryote	Eubacteria	Gammaproteobacteria	Gammaproteobacteria	Enterobacteriales	Enterobacteriaceae	Pantoea agglomerans			JGI Genome Portal	1	1	1	0	0	0	0	0
Eukaryote	Fungi	Ascomycota	Ascomycota	Saccharomycetales	Saccharomycetaceae	Saccharomyces cerevisiae			Saccharomyces Genome Database	1	0	1	0	0	0	0	0
Eukaryote	Fungi	Ascomycota	Ascomycota	Schizosaccharomycetales	Schizosaccaromycetaceae	Schizosaccharomyces pombe			JGI Genome Portal	1	0	1	0	0	0	0	0
Eukaryote	Protozoa	Amoebozoa	slime mold	Dictyosteliida	Dictyosteliidae	Dictyostelium discoideum			dictyBase	3	1	0	2	1	0	0	0
Eukaryote	Animalia	Arthropoda	insect	Diptera	Drosophilidae	Drosophila melanogaster			FlyBase	1	0	0	0	1	0	0	0
Eukaryote	Animalia	Chordata	mammal	Primates	Hominidae	Homo sapiens			KEGG GENOME	1	0	0	0	1	0	0	0
Eukaryote	Animalia	Chordata	mammal	Rodentia	Muridae	Mus musculus			KEGG GENOME	1	0	0	0	1	0	0	0
Eukaryote	Archaeplastida	Heterokontophyta	brown algae	Ectocarpales	Ectocarpaceae	Ectocarpus siliculosus			European Nucleotide Archive	1	0	0	1	0	0	0	0
Eukaryote	Archaeplastida	Rhodophyta	red algae	Cyanidiales	Cyanidiaceae	Cyanidioschyzon merolae	alCm		Cyanidioschyzon merolae Genome Project	1	0	0	1	0	0	0	0
Eukaryote	Archaeplastida	Chlorophyta	green algae	Chlorophyceae	Volvocaceae	Volvox carteri	alVc		Phytozome	1	0	0	1	0	0	0	0
Eukaryote	Archaeplastida	Chlorophyta	green algae	Chlorophyceae	Chlamydomonadaceae	Chlamydomonas reinhardtii	alChr		Phytozome	1	0	0	1	0	0	0	0
Eukaryote	Archaeplastida	Chlorophyta	green algae	Trebouxiohyceae	Chlorellaceae	Chlorella variabilis	alCv		JGI Genome Portal	1	0	0	1	0	0	0	0
Eukaryote	Archaeplastida	Chlorophyta	green algae	Trebouxiphyceae	Coccomyxaceae	Coccomyxa subellipsoidea	alCocc		Phytozome	1	0	0	1	0	0	0	0
Eukaryote	Archaeplastida	Chlorophyta	green algae	Mamiellophyceae	Mamiellaceae	Micromonas pusilla	alMp		Phytozome	2	0	0	1	0	1	0	0
Eukaryote	Archaeplastida	Chlorophyta	green algae	Mamiellophyceae	Bathycoccaceae	Ostreococcus lucimarinus	alOlt		Phytozome	2	0	0	1	0	1	0	0
Eukaryote	Sar	Ciliophora	Oligohymenophorea	Peniculida	Parameciidae	Paramecium tetraurelia			ParameciumDB	2	0	0	0	2	0	0	0
Eukaryote	Archaeplastida	Charophyta	Klebsormidiophyceae	Klebsormidiales	Klebsormidiaceae	Klebsormidium flaccidum	Klf		Klebsormidium flaccidum genome project	1	0	0	1	0	0	0	0
Eukaryote	Plantae	Marchantiophyta	Marchantiopsida	Marchantiales	Marchantiaceae	Marchantia polymorpha	Mapo		Phytozome	2	0	0	2	0	0	0	0
Eukaryote	Plantae	Bryophyta	Sphagnopsida	Sphagnales	Sphagnaceae	Sphagnum fallax	Spf		Phytozome	5	0	0	5	0	0	0	0
Eukaryote	Plantae	Bryophyta	Bryopsida	Funariales	Funariaceae	Physcomitrella patens	Pp		Phytozome	8	0*	0	8	0	0	0	0
Eukaryote	Plantae	Anthocerotopsida	Hornworts	Notothyladales	Notothyladaceae	Phaeoceros carolinianus	Phc		The 1000 plants	3	0	0	4	0	0	0	0

S1 Table. List of species used in this study and their classification, with the number of IPPT^{Pfam} and IPT^{Pfam} domain genes.

Classification										Num. of domain genes		Type of IPPT genes (see Table 1; Fig. 2)					
	Domain/ Kingdom	Phylum/Division/clade	Unranked/Class	Order	Family	Species	Abbreviation	Strain	Database	pIPPT	pIPT	A	B	C	D	E	F
Eukaryote	Plantae	Lycopodiophyta	Isoetopsida	Selaginellales	Selaginellaceae	Selaginella moellendorffii	Sm		Phytozome	1	0	0	1	0	0	0	0
Eukaryote	Plantae	Pteridophyta	Psilotopsida	Ophioglossales	Ophioglossaceae	Sceptridium dissectum	Scd		The 1000 plants	2	0	0	1	0	0	1	0
Eukaryote	Plantae	Pteridophyta	Pteridopsida	Gleicheniales	Dipteridaceae	Dipteris conjugata	Dco		The 1000 plants, USC fern genome db	2	0	0	1	0	0	1	0
Eukaryote	Plantae	Pteridophyta	Polypodiopsida	Polypodiales	Woodsiaceae	Cystopteris fragilis	Cfr		The 1000 plants	2	0	0	1	0	0	1	0
Eukaryote	Plantae	Pteridophyta	Polypodiopsida	Polypodiales	Polypodiaceae	Polypodium hesperium	Poh		The 1000 plants	2	0	0	1	0	0	1	0
Eukaryote	Plantae	Phinophyta	Gymnosperms	Pinales	Pinaceae	Picea abies	Pa		Congenie.org	3	0	0	1	0	0	2	0
Eukaryote	Plantae	Phinophyta	Gymnosperms	Pinales	Pinaceae	Pinus taeda	Pit		Dendrome Project; Congenie.org	2	0	0	1	0	0	2	0
Eukaryote	Plantae	Angiosperms	Basal angiosperms	Amborellales	Amborellaceae	Amborella trichopoda	Amt		Phytozome	4	0	0	1	0	0	1	2
Eukaryote	Plantae	Angiosperms	Monocots	Zingiberales	Musaceae	Musa acuminata	Mua		Phytozome	8	0*	0	1	0	0	1	6
Eukaryote	Plantae	Angiosperms	Monocots	Poales	Poaceae	Brachypodium distachyon	Bd		Phytozome	9	0	0	1	0	0	1	7
Eukaryote	Plantae	Angiosperms	Monocots	Poales	Poaceae	Zea mays	Zm		Phytozome	11	0	0	1	0	0	1	9
Eukaryote	Plantae	Angiosperms	Monocots	Poales	Poaceae	Oryza sativa	Os		Phytozome	10	0	0	1	0	0	1	8
Eukaryote	Plantae	Angiosperms	Monocots	Poales	Poaceae	Sorghum bicolor	Sb		Phytozome	8	0	0	2	0	0	1	5
Eukaryote	Plantae	Angiosperms	Eudicots	Ranunculales	Ranunculaceae	Aquilegia coerulea	Aq		Phytozome	5	0	0	1	0	0	1	3
Eukaryote	Plantae	Angiosperms	Rosids	Brassicales	Brassicaceae	Arabidopsis thaliana	At		Genbank	9	0	0	1	0	0	1	7
Eukaryote	Plantae	Angiosperms	Rosids	Brassicales	Brassicaceae	Brassica rapa	Br		Phytozome	13	0	0	2	0	0	1	10
Eukaryote	Plantae	Angiosperms	Rosids	Cucurbitales	Cucurbitaceae	Cucumis sativus	Cs		Phytozome	7	0	0	1	0	0	1	5
Eukaryote	Plantae	Angiosperms	Rosids	Fabales	Fabaceae	Lotus japonicus	Lj		PlantGDB	6	0	0	1	0	0	1	4
Eukaryote	Plantae	Angiosperms	Rosids	Fabales	Fabaceae	Medicago truncatula	Mt		Phytozome	6	0	0	1	0	0	1	4
Eukaryote	Plantae	Angiosperms	Rosids	Malpighiales	Salicaceae	Populus trichocarpa	Pt		Phytozome	8	0	0	1	0	0	1	6
Eukaryote	Plantae	Angiosperms	Core eudicots	Caryophyllales	Chenopodiaceae	Beta vulgaris	Bv		The Beta vulgaris Resource	5	0	0	1	0	0	1	3
Eukaryote	Plantae	Angiosperms	Asterids	Lamiales	Phrymaceae	Erythranthe (Mimulus) guttata	Mg		Phytozome	7	0	0	1	0	0	1	5
Eukaryote	Plantae	Angiosperms	Asterids	Lamiales	Gesneriaceae	Streptocarpus rexii	Sr		AngelDust	5	0	0	1	0	0	1	3
Eukaryote	Plantae	Angiosperms	Asterids	Solanales	Solanaceae	Solanum lycopersicum	Sl		Genbank	6	0*	0	1	0	0	1	4
Eukaryote	Plantae	Angiosperms	Asterids	Solanales	Solanaceae	Solanum tuberosum	St		Phytozome	5	0*	0	1	0	0	1	3

*IPT domain registered in Pfam showed more similarity to IPPTdomain
