# Supplementary material for: Tangled history of a multigene family: The evolution of ISOPENTENYLTRANSFERASE genes
Source: PLoS One. 2018 Aug 2;13(8):e0201198. doi: 10.1371/journal.pone.0201198 (PMC6071968; doi:10.1371/journal.pone.0201198)
Supplement: S2 Table — Gene ID is the ID used in the large phylogeny in Fig 3. Asterisks indicate the gene name retrieved from Frébort et al. (2011) [18]. (PDF) [file pone.0201198.s017.pdf]

S2 Table. Gene accession numbers used in this study. Gene ID is the ID used in the large phylogeny in Fig. 3. Asterisks indicate the gene name retrieved from Frébort et al. (2011).

Species	Gene	Gene ID in Fig. 3	Gene Accession Number	Pfam Domain	Num. of Exon
Aquifex aeolicus VF5	MiaA	MIAA_AQUAE	WP_010880663	IPPT	1
Thermotoga maritima MSB8	MiaA		TM0525	IPPT	1
Listeria innocua	MiaA		CAC96563	IPPT	1
Listeria monocytogenes EGD-e	MiaA		NP_464819	IPPT	1
Bacillus subtilis	MiaA	MIAA_BACSU	WP_048654889	IPPT	1
Bacillus halodurans	MiaA		BAB06085	IPPT	1
Bacillus megaterium QM B1551	MiaA		ADE71118	IPPT	1
Staphylococcus aureus N315	MiaA		SA1144	IPPT	1
Lactococcus lactis subsp. lactis Il1403	MiaA		NP_266782	IPPT	1
Clostridium perfringens 13	MiaA		BAB80863	IPPT	1
Fusobacterium nucleatum	MiaA		NP_602717	IPPT	1
Prochlorococcus marinus	MiaA	MIAA_PROMA	WP_012196313	IPPT	1
Coleofasciculus chthonoplastes	MiaA	McIPT*	ZP_05024550, WP_006099226	IPPT	1
Synechocystis sp.	MiaA	MIAA_SYNY3	WP_010872740	IPPT	1
Pseudanabaena sp. PCC 7367	MiaA		AFY70076	IPPT	1
Thermosynechococcus elongatus	MiaA		NP_681437	IPPT	1
Acaryochloris marina	MiaA	AmIPT*	YP_001518792	IPPT	1
Synechococcus elongatus PCC6301	MiaA		BAD79806	IPPT	1
Geitlerinema sp. PCC 7407	MiaA		AFY65513	IPPT	1
Microcoleus sp. PCC 7113	MiaA		AFZ17274	IPPT	1
Trichodesmium erythraeum	MiaA		ABG52987	IPPT	1
Chroococcidiopsis thermalis PCC 7203	MiaA		AFY85865	IPPT	1
Fischerella sp. NIES-3754	MiaA		BAU07238	IPPT	1
Nodularia spumigena	MiaA	NsIPT*	ZP_01631398, WP_006197862	IPPT	1
Anabaena variabilis	IPT	AvIPT1	Q3M9L2; YP323219	IPT	1
Anabaena variabilis	MiaA	AvIPT2	YP_323028	IPPT	1
Nostoc sp. PCC7120	IPT	NoIPT1	Q8Z078; BAB77744	IPT	1
Nostoc sp. PCC7120	MiaA	NoIPT2	NP_489306	IPPT	1
Cylindrospermum stagnale	MiaA		AFZ25589	IPPT	1
Anabaena cylindrica PCC 7122	MiaA		AFZ58152	IPPT	1
Streptomyces coelicolor A3(2)	MiaA	MIAA_STRCO	WP_011030456	IPPT	1
Mycobacterium tuberculosis	MiaA	MIAA_MYCTU	Rv2727c	IPPT	1
Rhodococcus jostii	MiaA	RjIPT*	YP_706705	IPPT	1
Rhodococcus erythropsis	MiaA	MIAA_RHOER	RER_27630	IPPT	1
Rhodococcus fascians	IPT	IPT_RHOFA	WP_015586134	IPT	1
Rhodococcus fascians	MiaA	MIAA_RHOFA	WP_037142908	IPPT	1
Chlamydia muridarum Nigg	MiaA		AAF39024	IPPT	1
Borrelia burgdorferi B31	MiaA		NP_212955	IPPT	1
Treponema pallidum subsp. pallidum Nichols	MiaA		AAC65611	IPPT	1
Borrelia burgdorferi	MiaA	MIAA_BORBU	WP_010889830	IPPT	1
Helicobacter pylori Rif1	MiaA	MIAA.HELPY	WP_000338435	IPPT	1
Campylobacter jejuni RM1221	MiaA		AAW34756	IPPT	1
Agrobacterium tumefaciens (fabrum)	TZS	IPTZ_AGRT5	NP_396682	IPT	1
Agrobacterium tumefaciens (fabrum)	IPT	IPTZ_AGRT7	NP_396529	IPT	1
Agrobacterium tumefaciens (fabrum)	MiaA	MIAA.RHIRD	NP_355007	IPPT	1
Agrobacterium tumefaciens	IPT	IPT_RHIAD	Q9R466	IPT	1
Agrobacterium vitis	IPT	IPT1_AGRVS	Avi_8294	IPT	1
Agrobacterium vitis	MiaA	MIAA_AGRVS	Avi_2808	IPPT	1
Rickettsia conorii	MiaA		AAL03194	IPPT	1
Sinorhizobium meliloti 1021	MiaA		NP_386224	IPPT	1
Brucella melitensis	MiaA		ACO01152	IPPT	1
Ralstonia solanacearum GMI1000	IPT		CAD18376	IPT	1
Ralstonia solanacearum GMI1000	MiaA		CAD16271	IPPT	1
Neisseria meningitidis MC58	MiaA		NP_273973	IPPT	1
Escherichia coli	MiaA	MIAA_ECOLI	WP_032208303	IPPT	1
Haemophilus influenzae Rd KW20	MiaA	MIAA_HAEIN	WP_014326548	IPPT	1
Pseudomonas putida	MiaA	MIAA_PSEPU	O30762	IPPT	1
Pantoea ananatis	MiaA	MIAA_PAANA	PP_4895	IPPT	1
Pantoea agglomerans	IPT	IPT_PANAY	Q47851	IPT	1
Pantoea agglomerans	MiaA	MIAA_PANAY	AMG59936.1	IPPT	1
Saccharomyces cerevisiae	Mod5	MOD5_YEAST	CAA99499	IPPT	1
Schizosaccharomyces pombe	Tit1	TIT1_SCHPO	NP_593436	IPPT	1
Dictyostelium discoideum	iptA	IPT_DICDI	EAL68793	IPT	1
Dictyostelium discoideum	iptB	MIAA_DICDIb	EAL61748	IPPT	2
Dictyostelium discoideum	iptC1	MIAA_DICDIc1	EAL71157	IPPT	3
Dictyostelium discoideum	iptC2	MIAA_DICDIc2	EAL70442	IPPT	3
Drosophila melanogaster	tRNA dimethyltransferase	DmtRdim	NP_733057	IPPT	1
Homo sapiens	Trit1	TRIT1_HUMAN	CAG33507	IPPT	11
Mus musculus	Trit1	TRIT1_MOUSE	AAH19812	IPPT	11
Cyanidioschyzon merolae	class I tRNA-IPT	alCmtIPT_I	CMS475C	IPPT	1
Ectocarpus siliculosus	class I tRNA-IPT	alEstIPT_I	Esi0002_0108	IPPT	7
Volvox carteri	class I tRNA-IPT	alVctIPT_I	D8TTA6	IPPT	11
Chlamydomonas reinhardtii	class I tRNA-IPT	alChrtIPT_I	Cre17.g717350	IPPT	9
Chlorella variabilis	class I tRNA-IPT	alCvtIPT_I	E1ZS74	IPPT	9
Coccomyxa subellipsoidea	class I tRNA-IPT	alCocctIPT_I	3943	IPPT	7
Micromonas pusilla	class I tRNA-IPT	alMptIPT_I	MicpuC2.gw1.11.144.1	IPPT	1
Micromonas pusilla	prasinophyte tRNA-IPT	alMptIPT_P	gm1.911_g	IPPT	1
Osteococcus lucimarinus	class I tRNA-IPT	alOltIPT_I	gwEuk.6.256.1	IPPT	1
Osteococcus lucimarinus	prasinophyte tRNA-IPT	alOltIPT_P	e_gwEuk.3.212.1	IPPT	1
Paramecium tetraurelia	IPPT1	IPPT1_Paramecium	GSPATP00010366001	IPPT	3
Paramecium tetraurelia	IPPT2	IPPT2_Paramecium	GSPATP00037654001	IPPT	2
Klebsormidium flaccidum	class I tRNA-IPT	KlfltIPT_I	kfl00008_0210	IPPT	7
Marchantia polymorpha	class I tRNA-IPT	MapotIPT_Ia	Mapoly0033s0012.1	IPPT	11
Marchantia polymorpha	class I tRNA-IPT	MapotIPT_Ib	Mapoly0012s0089.1	IPPT	1
Sphagnum fallax	class I tRNA-IPT	SpftIPT_Ia	Sphfalx0166s0043.1	IPPT	11
Sphagnum fallax	class I tRNA-IPT	SpftIPT_Ib	Sphfalx0339s0009.1	IPPT	2
Sphagnum fallax	class I tRNA-IPT	SpftIPT_Ic	Sphfalx0000s0100.1	IPPT	11

S2 Table. Gene accession numbers used in this study. Gene ID is the ID used in the large phylogeny in Fig. 3. Asterisks indicate the gene name retrieved from Frébort et al. (2011).

Species	Gene	Gene ID in Fig. 3	Gene Accession Number	Pfam Domain	Num. of Exon
Sphagnum fallax	class I tRNA-IPT	SpftIPT_Id	Sphfalx0095s0039.1	IPPT	11
Sphagnum fallax	class I tRNA-IPT	SpftIPT_Ie	Sphfalx0030s0078.1	IPPT	11
Physcomitrella patens	class I tRNA-IPT	PpIPT1	B3XXG1	IPPT	11
Physcomitrella patens	class I tRNA-IPT	PpIPT2_1	A9SYC6;Pp3c23_12330V3.1	IPPT	2
Physcomitrella patens	class I tRNA-IPT	PpIPT2_2	Pp3c23_12150V3.1	IPPT	2
Physcomitrella patens	class I tRNA-IPT	PpIPT2_3	Pp3c23_12370V3.1	IPPT	2
Physcomitrella patens	class I tRNA-IPT	PpIPT3	A9TPE9; Pp3c27_70V3.3	IPPT	11
Physcomitrella patens	class I tRNA-IPT	PpIPT4	A9SC20; Pp3c5_9400V3.1	IPPT	11
Physcomitrella patens	class I tRNA-IPT	PpIPT5	A9RKP1; Pp3c6_21360V3.1	IPPT	11
Physcomitrella patens	class I tRNA-IPT	PpIPT6	A9TW02; Pp3c16_6080V3.1	IPPT	11
Phaeoceros carolinianus	class I tRNA-IPT	PhctIPT_Ia	Phc2120145	IPPT	n/e
Phaeoceros carolinianus	class I tRNA-IPT	PhctIPT_Ib	Phc2004195	IPPT	n/e
Phaeoceros carolinianus	class I tRNA-IPT	PhctIPT_Ic	Phc2135914	IPPT	n/e
Selaginella moellendorffii	class I tRNA-IPT	SmtIPT_I	D8RG81	IPPT	11
Sceptridium dissectum	class I tRNA-IPT	ScdtIPT_I	EEAQ_2085945	IPPT	n/e
Sceptridium dissectum	class II tRNA-IPT	ScdtIPT_II	EEAQ_2015688	IPPT	n/e
Dipteris conjugata	class I tRNA-IPT	DcotIPT_I	MEKP_2106424	IPPT	n/e
Dipteris conjugata	class II tRNA-IPT	DcotIPT_II	MEKP_2101411	IPPT	n/e
Cystopteris fragilis	class I tRNA-IPT	CfrtIPT_I	XWDM_2029675	IPPT	n/e
Cystopteris fragilis	class II tRNA-IPT	CfrtIPT_II	XWDM_2017467; XWDM_2127682	IPPT	n/e
Polypodium hesperium	class I tRNA-IPT	PohtIPT_I	ZRAV_2012801	IPPT	n/e
Polypodium hesperium	class II tRNA-IPT	PohtIPT_II	ZRAV_2135602	IPPT	n/e
Picea abies	class II tRNA-IPT	PatIPT_IIa	MA_8139065g0010	IPPT	9
Picea abies	class II tRNA-IPT	PatIPT_Iib	MA_20274g0010; MA_20274g0020	IPPT	13
Picea abies	class I tRNA-IPT	PatIPT_I	MA_10433765g0010	IPPT	4
Pinus taeda	class I tRNA-IPT	PittIPT_I	5A_I16_NT_comp44677_c0_seq1	IPPT	n/e
Pinus taeda	class II tRNA-IPT	PittIPT_IIa	_000003401; 1A_I11_VO_L_1411_	IPPT	n/e
Pinus taeda	class II tRNA-IPT	PittIPT_IIb	PITA_000003404	IPPT	n/e
Amborella trichopoda	adenosine-phosphate IPT	AmtaIPT_a	AmTr_v1.0_scaffold00025.75	IPPT	1
Amborella trichopoda	adenosine-phosphate IPT	AmtaIPT_b	AmTr_v1.0_scaffold00079.46	IPPT	1
Amborella trichopoda	class II tRNA-IPT	AmttIPT_II	AmTr_v1.0_scaffold00095.58	IPPT	11
Amborella trichopoda	class I tRNA-IPT	AmttIPT_I	AmTr_v1.0_scaffold00021.85	IPPT	12
Musa acuminata	class I tRNA-IPT	MuatIPT_I	GSMUA_Achr1T06960	IPPT	2
Musa acuminata	class II tRNA-IPT	MuatIPT_II	GSMUA_Achr11T12450	IPPT	11
Musa acuminata	adenosine-phosphate IPT	MuaaIPT_a	GSMUA_Achr1T12830	IPPT	2
Musa acuminata	adenosine-phosphate IPT	MuaaIPT_b	GSMUA_Achr1T23110	IPPT	1
Musa acuminata	adenosine-phosphate IPT	MuaaIPT_c	GSMUA_Achr4T06810	IPPT	1
Musa acuminata	adenosine-phosphate IPT	MuaaIPT_d	GSMUA_Achr9T21400	IPPT	2
Musa acuminata	adenosine-phosphate IPT	MuaaIPT_e	GSMUA_Achr3T29480	IPPT	2
Musa acuminata	adenosine-phosphate IPT	MuaaIPT_f	GSMUA_Achr3T09080	IPPT	2
Brachypodium distachyon	adenosine-phosphate IPT	BdaIPT_a	Bradi2g17400	IPPT	1
Brachypodium distachyon	adenosine-phosphate IPT	BdaIPT_b	Bradi2g46920	IPPT	1
Brachypodium distachyon	adenosine-phosphate IPT	BdaIPT_c	Bradi1g04580	IPPT	1
Brachypodium distachyon	adenosine-phosphate IPT	BdaIPT_d	Bradi1g54060	IPPT	1
Brachypodium distachyon	adenosine-phosphate IPT	BdaIPT_e	Bradi2g13410	IPPT	1
Brachypodium distachyon	adenosine-phosphate IPT	BdaIPT_f	Bradi4g27861	IPPT	1
Brachypodium distachyon	adenosine-phosphate IPT	BdaIPT_g	Bradi1g61960	IPPT	1
Brachypodium distachyon	class II tRNA-IPT	BdtIPT_II	Bradi1g70200	IPPT	10
Brachypodium distachyon	class I tRNA-IPT	BdtIPT_I	Bradi1g29485	IPPT	4
Zea mays	class II tRNA-IPT	ZmIPT1	GRMZM2G097258	IPPT	10
Zea mays	adenosine-phosphate IPT	ZmIPT2	GRMZM2G084462	IPPT	1
Zea mays	adenosine-phosphate IPT	ZmIPT3a	GRMZM2G415751	IPPT	1
Zea mays	adenosine-phosphate IPT	ZmIPT3b	GRMZM2G393014	IPPT	1
Zea mays	adenosine-phosphate IPT	ZmIPT4	GRMZM2G104559	IPPT	1
Zea mays	adenosine-phosphate IPT	ZmIPT5	AC210013.4_FG005	IPPT	1
Zea mays	adenosine-phosphate IPT	ZmIPT6	GRMZM2G116878	IPPT	1
Zea mays	adenosine-phosphate IPT	ZmIPT7	GRMZM2G436770	IPPT	1
Zea mays	adenosine-phosphate IPT	ZmIPT8	GRMZM2G025429	IPPT	1
Zea mays	adenosine-phosphate IPT	ZmIPT9	GRMZM2G018046	IPPT	1
Zea mays	class I tRNA-IPT	ZmIPT10	GRMZM2G102915	IPPT	1
Oryza sativa	adenosine-phosphate IPT	OsIPT1	AB239797	IPPT	1
Oryza sativa	adenosine-phosphate IPT	OsIPT2	AB239798	IPPT	1
Oryza sativa	adenosine-phosphate IPT	OsIPT3	AB239799	IPPT	1
Oryza sativa	adenosine-phosphate IPT	OsIPT4	AB239800	IPPT	1
Oryza sativa	adenosine-phosphate IPT	OsIPT5	AB239801	IPPT	1
Oryza sativa	adenosine-phosphate IPT	OsIPT6	AB239807	IPPT	1
Oryza sativa	adenosine-phosphate IPT	OsIPT7	AB239804	IPPT	1
Oryza sativa	adenosine-phosphate IPT	OsIPT8	AB853903	IPPT	1
Oryza sativa	class II tRNA-IPT	OsIPT9	AB239806	IPPT	10
Oryza sativa	class I tRNA-IPT	OsIPT10	AB239807	IPPT	1
Sorghum bicolor	adenosine-phosphate IPT	SbaIPT_a	Sobic.009G223100	IPPT	1
Sorghum bicolor	adenosine-phosphate IPT	SbaIPT_b	Sobic.003G261700	IPPT	1
Sorghum bicolor	adenosine-phosphate IPT	SbaIPT_c	Sobic.002G076700	IPPT	1
Sorghum bicolor	adenosine-phosphate IPT	SbaIPT_d	Sobic.003G162200	IPPT	1
Sorghum bicolor	adenosine-phosphate IPT	SbaIPT_e	Sobic.006G059900	IPPT	1
Sorghum bicolor	class II tRNA-IPT	SbtIPT_II	Sobic.003G442000	IPPT	10
Sorghum bicolor	class I tRNA-IPT	SbtIPT_Ia	Sobic.002G407800	IPPT	1
Sorghum bicolor	class I tRNA-IPT	SbtIPT_Ib	Sobic.010G277700	IPPT	2
Aquilegia coerulea	adenosine-phosphate IPT	AqaIPT_a	Aquca_026_00321	IPPT	1
Aquilegia coerulea	adenosine-phosphate IPT	AqaIPT_b	Aquca_135_00019	IPPT	1
Aquilegia coerulea	adenosine-phosphate IPT	AqaIPT_c	Aquca_008_00244	IPPT	1
Aquilegia coerulea	class II tRNA-IPT	AqtIPT_II	Aquca_122_00011	IPPT	10
Aquilegia coerulea	class I tRNA-IPT	AqtIPT_I	Aquca_055_00066	IPPT	11
Arabidopsis thalliana	adenosine-phosphate IPT	AtIPT1	NM_105517	IPPT	1
Arabidopsis thalliana	class II tRNA-IPT	AtIPT2	NM_128335	IPPT	10
Arabidopsis thalliana	adenosine-phosphate IPT	AtIPT3	NM_116176	IPPT	1
Arabidopsis thalliana	adenosine-phosphate IPT	AtIPT4	NM_118598	IPPT	1

S2 Table. Gene accession numbers used in this study. Gene ID is the ID used in the large phylogeny in Fig. 3. Asterisks indicate the gene name retrieved from Frébort et al. (2011).

Species	Gene	Gene ID in Fig. 3	Gene Accession Number	Pfam Domain	Num. of Exon
Arabidopsis thalliana	adenosine-phosphate IPT	AtIPT5	NM_121909	IPPT	1
Arabidopsis thalliana	adenosine-phosphate IPT	AtIPT6	NM_102352	IPPT	1
Arabidopsis thalliana	adenosine-phosphate IPT	AtIPT7	NM_113267	IPPT	1
Arabidopsis thalliana	adenosine-phosphate IPT	AtIPT8	NM_112803	IPPT	2
Arabidopsis thalliana	class I tRNA-IPT	AtIPT9	NM_001203415	IPPT	11
Brassica rapa	adenosine-phosphate IPT	BrIPT1_1	Brara.G02522	IPPT	1
Brassica rapa	adenosine-phosphate IPT	BrIPT1_2	Brara.B01793	IPPT	1
Brassica rapa	class II tRNA-IPT	BrIPT2	Brara.D01658	IPPT	10
Brassica rapa	adenosine-phosphate IPT	BrIPT3_1	Brara.D00029	IPPT	1
Brassica rapa	adenosine-phosphate IPT	BrIPT3_2	Brara.I04365	IPPT	1
Brassica rapa	adenosine-phosphate IPT	BrIPT5_1	Brara.J01679	IPPT	1
Brassica rapa	adenosine-phosphate IPT	BrIPT5_2	Brara.B00796	IPPT	1
Brassica rapa	adenosine-phosphate IPT	BrIPT7_1	Brara.G00714	IPPT	1
Brassica rapa	adenosine-phosphate IPT	BrIPT7_2	Brara.A02630	IPPT	1
Brassica rapa	adenosine-phosphate IPT	BrIPT8_1	Brara.A02893	IPPT	2
Brassica rapa	adenosine-phosphate IPT	BrIPT8_2	Brara.C03730	IPPT	2
Brassica rapa	class I tRNA-IPT	BrIPT9_1	Brara.C00907	IPPT	11
Brassica rapa	class I tRNA-IPT	BrIPT9_2	Brara.B00861	IPPT	11
Cucumis sativus	adenosine-phosphate IPT	CsIPT1	Cucsa.253940	IPPT	1
Cucumis sativus	adenosine-phosphate IPT	CsIPT2	Cucsa.283510	IPPT	1
Cucumis sativus	class I tRNA-IPT	CsIPT3	Cucsa.273490	IPPT	11
Cucumis sativus	class II tRNA-IPT	CsIPT4	Cucsa.199980	IPPT	10
Cucumis sativus	adenosine-phosphate IPT	CsIPT5	Cucsa.240260	IPPT	2
Cucumis sativus	adenosine-phosphate IPT	CsIPT6	Cucsa.260900	IPPT	1
Cucumis sativus	adenosine-phosphate IPT	CsIPT7	Cucsa.365450	IPPT	1
Lotus japonicus	adenosine-phosphate IPT	LjIPT1	DQ436462	IPPT	1
Lotus japonicus	adenosine-phosphate IPT	LjIPT2	DQ436463	IPPT	1
Lotus japonicus	adenosine-phosphate IPT	LjIPT3	DQ436464	IPPT	1
Lotus japonicus	adenosine-phosphate IPT	LjIPT4	DQ436465	IPPT	2
Lotus japonicus	class II tRNA-IPT	LjIPT5	EU195535	IPPT	10
Lotus japonicus	class I tRNA-IPT	LjIPT6	LjSGA_068775	IPPT	11
Medicago truncatula	adenosine-phosphate IPT	MtaIPT_a	Medtr1g110590	IPPT	1
Medicago truncatula	adenosine-phosphate IPT	MtaIPT_b	Medtr1g072540	IPPT	1
Medicago truncatula	adenosine-phosphate IPT	MtaIPT_c	Medtr4g117330	IPPT	1
Medicago truncatula	adenosine-phosphate IPT	MtaIPT_d	Medtr2g022140	IPPT	1
Medicago truncatula	class II tRNA-IPT	MttIPT_II	Medtr4g055110	IPPT	10
Medicago truncatula	class I tRNA IPT	MttIPT_I	Medtr2g078120	IPPT	11
Populus trichocarpa	adenosine-phosphate IPT	PtaIPT_a	Potri.008G121500	IPPT	1
Populus trichocarpa	adenosine-phosphate IPT	PtaIPT_b	Potri.010G123900	IPPT	2
Populus trichocarpa	adenosine-phosphate IPT	PtaIPT_c	Potri.014G139300	IPPT	1
Populus trichocarpa	adenosine-phosphate IPT	PtaIPT_d	Potri.008G202200	IPPT	1
Populus trichocarpa	adenosine-phosphate IPT	PtaIPT_e	Potri.010G030500	IPPT	1
Populus trichocarpa	adenosine-phosphate IPT	PtaIPT_f	Potri.008G033300	IPPT	5
Populus trichocarpa	class II tRNA IPT	PttIPT_II	Potri.009G147600	IPPT	11
Populus trichocarpa	class I tRNA IPT	PttIPT_I	Potri.001G376600	IPPT	11
Beta vulgaris	adenosine-phosphate IPT	BvaIPT_a	Bv6_139300_ctns	IPPT	1
Beta vulgaris	adenosine-phosphate IPT	BvaIPT_b	Bv7_163440_zorj	IPPT	1
Beta vulgaris	adenosine-phosphate IPT	BvaIPT_c	Bvu_00420_aqe1	IPPT	1
Beta vulgaris	class I tRNA IPT	BvtIPT_II	Bv7_179790_odyi1	IPPT	10
Beta vulgaris	class II tRNA IPT	BvtIPT_I	Bv4_085070_mmpt.t1	IPPT	11
Erythranthe (Mimulus) guttata	adenosine-phosphate IPT	MgaIPT_a	Mgv1a010491	IPPT	1
Erythranthe (Mimulus) guttata	adenosine-phosphate IPT	MgaIPT_b	Mgv1a017953	IPPT	1
Erythranthe (Mimulus) guttata	adenosine-phosphate IPT	MgaIPT_c	Mgv1a026307	IPPT	1
Erythranthe (Mimulus) guttata	adenosine-phosphate IPT	MgaIPT_d	Mgv1a022025	IPPT	1
Erythranthe (Mimulus) guttata	adenosine-phosphate IPT	MgaIPT_e	Mgv1a020459	IPPT	2
Erythranthe (Mimulus) guttata	class II tRNA IPT	MgtIPT_II	Mgv1a025771	IPPT	10
Erythranthe (Mimulus) guttata	class I tRNA IPT	MgtIPT_I	Mgv1a026415	IPPT	11
Solanum lycopersicum	adenosine-phosphate IPT	SlIPT1	NM_001257984	IPPT	1
Solanum lycopersicum	adenosine-phosphate IPT	SlIPT2	NM_001257985	IPPT	1
Solanum lycopersicum	adenosine-phosphate IPT	SlIPT3	NM_001279341	IPPT	1
Solanum lycopersicum	adenosine-phosphate IPT	SlIPT4	NM_001257986	IPPT	1
Solanum lycopersicum	class II tRNA IPT	SlIPT5	NM_001257987	IPPT	8
Solanum lycopersicum	class I tRNA IPT	SlIPT6	XR_738728; Solyc12g014190.1	IPPT	11
Solanum tuberosum	adenosine-phosphate IPT	StaIPT_a	PGSC0003DMT400015411	IPPT	1
Solanum tuberosum	adenosine-phosphate IPT	StaIPT_b	PGSC0003DMT400037749	IPPT	1
Solanum tuberosum	adenosine-phosphate IPT	StaIPT_c	PGSC0003DMT400002509	IPPT	1
Solanum tuberosum	class II tRNA IPT	SttIPT_II	PGSC0003DMT400068271	IPPT	9
Solanum tuberosum	class I tRNA IPT	SttIPT_I	PGSC0003DMT400039795	IPPT	11
Streptocarpus rexii	adenosine-phosphate IPT	SrIPT1	MH230161	IPPT	1
Streptocarpus rexii	class II tRNA IPT	SrIPT2	MH230162	IPPT	7
Streptocarpus rexii	adenosine-phosphate IPT	SrIPT3	MH230163	IPPT	1
Streptocarpus rexii	adenosine-phosphate IPT	SrIPT5	MH230164	IPPT	1
Streptocarpus rexii	class I tRNA IPT	SrIPT9	MH230165	IPPT	11
