# Supplementary material for: Tangled history of a multigene family: The evolution of ISOPENTENYLTRANSFERASE genes
Source: PLoS One. 2018 Aug 2;13(8):e0201198. doi: 10.1371/journal.pone.0201198 (PMC6071968; doi:10.1371/journal.pone.0201198)
Supplement: S3 Table — (PDF) [file pone.0201198.s018.pdf]

S3 Table. References used for summary of gene expression in S15 Fig. R: root, L: leaf, Fr: flower, Fl: fruit are the categories shown in S15 Fig. in which the sample information is shown

Taxon	References	Methods	Tissues examined and summarized in S15 Fig.
Physcomitrella patens	Patil and Nicander 2011	RT-PCR	Whole organ;<br>Protonema stage, mature gametophytic stage, sporophytic stage
Picea abies	Nystedt et al. 2013	RNA-seq	Needles (L), cones (Fr)
Oryza sativa	Sakamoto et al. 2006	RT-PCR	Root (R), leaf blade (L), flower (Fr)
Zea mays	Vyroubalová et al. 2009	Real-time PCR	Mature root (R), mature leaf (L), silk + tassel (Fl)[a],<br>embryo + endosperm (Fr)[b]
Arabidopsis thaliana	Miayawaki et al. 2004	RT-PCR; promoter:GUS	Root (R), leaf (L), open flowers (Fl), siliques (Fr),
Brassica rapa	Ando et al. 2005;<br>Liu et al. 2013	Northern blotting;<br>real-time PCR	Root (R), leaf (L), flower (Fl),
Cucumis sativus	Zhang et al. 2013	Real-time PCR	Root (R), leaf (L), flowers (Fl), immature siliques (Fr)
Lotus japonicus	Chen et al. 2014	RT-PCR	Root (R), leaf (L), flower (Fl), silique (Fr)
Solanum lycopersicum	Matsuo et al. 2012	Real-time PCR	Root (R), leaf (L), flower (Fl), fruit (Fr)
Steptocarpus rexii	Chen et al. 2017	Real-time PCR; RT-PCR	Root (R), phyllomorph (L), open flower (Fl), fruit (Fr),

a original data shown separately silk and tassel, and the values are combined as the expression of flower in S15 Fig. for simplicity

b original data shown separately embryo and endosperm, and the values are combined as the expression of fruit in S15 Fig. for simplicity

Additional references for S3 Table

1. Ando S, Asano T, Tsushima S, Kamachi S, Hagio T, Tabei Y. Changes in gene expression of putative isopentenyltransferase during clubroot development in Chinese cabbage (*Brassica rapa* L.). Physiol Mol Plant Pathol. 2005; 67: 59-67.

2. Chen Y, Chen W, Li X, Jiang H, Wu P, Xia K, et al. Knockdown of *LjIPT3* influences nodule development in *Lotus japonicus*. Plant Cell Physiol. 2014; 55: 183-193.

3. Chen YY, Nishii K, Spada A, Wang CN, Sakakibara H, Kojima M, et al. Cytokinin biosynthesis *ISOPENTENYLTRANSFERASE* genes are differentially expressed during phyllomorph development in the acaulescent *Streptocarpus rexii* (Gesneriaceae). South Afr J Bot. 2017; 109: 96-111.

4. Liu Z, Lv Y, Zhang M, Liu Y, Kong L, Zou M, et al. Identification, expression, and comparative genomic analysis of the IPT and CKX gene families in Chinese cabbage (*Brassica rapa ssp. pekinensis*). BMC Genomics. 2013; 14: 594.

5. Vyroubalová S, Václavíková K, Turečková V, Novák O, Šmehilová M, Hluska T, et al. Characterization of new maize genes putatively involved in cytokinin metabolism and their expression during osmotic stress in relation to cytokinin levels. Plant Physiol. 2009; 151: 433-447A.

6. Zhang T, Li J, Li C, Peng S, Xu J, Chen J. Identification and characterization of *CsIPT* genes in cucumber. Acta Hort Sinica. 2013; 40: 58-68.
