# Supplementary material for: Tangled history of a multigene family: The evolution of ISOPENTENYLTRANSFERASE genes
Source: PLoS One. 2018 Aug 2;13(8):e0201198. doi: 10.1371/journal.pone.0201198 (PMC6071968; doi:10.1371/journal.pone.0201198)
Supplement: S4 Table — (PDF) [file pone.0201198.s019.pdf]

S4 Table. References used for cytokinin interaction in S15 Fig.

Taxon	Reference	Methods	Tissues examined	Chemicals used as cytokinin
Oryza sativa	Sakamoto et al. 2006	Real-time PCR	Whole seedling (◸ upper triangle)	iP
Zea mays	Vyroubalová et al. 2009	Real-time PCR	Leaf (◸), root (◺ lower triangle)	BAP
Arabidopsis thaliana	Miyawaki et al. 2004	RT-PCR	Root (◺)	BAP
Brassica rapa	Liu et al. 2013	Real-time PCR	Leaf (◸)	BAP
Streptocarpus rexii	Chen et al. 2017	Real-time PCR	Seedling (◸)	BAP

Additional references for S4 Table

1. Chen YY, Nishii K, Spada A, Wang CN, Sakakibara H, Kojima M, et al. Cytokinin biosynthesis *ISOPENTENYLTRANSFERASE* genes are differentially expressed during phyllomorph development in the acaulescent *Streptocarpus rexii* (Gesneriaceae). South Afr J Bot. 2017; 109: 96-111.

2. Liu Z, Lv Y, Zhang M, Liu Y, Kong L, Zou M, et al. Identification, expression, and comparative genomic analysis of the IPT and CKX gene families in Chinese cabbage (*Brassica rapa ssp. pekinensis*). BMC Genomics. 2013; 14: 594.

3. Vyroubalová S, Václavíková K, Turečková V, Novák O, Šmehilová M, Hluska T, et al. Characterization of new maize genes putatively involved in cytokinin metabolism and their expression during osmotic stress in relation to cytokinin levels. Plant Physiol. 2009; 151: 433-447A.
