# Supplementary material for: Tangled history of a multigene family: The evolution of ISOPENTENYLTRANSFERASE genes
Source: PLoS One. 2018 Aug 2;13(8):e0201198. doi: 10.1371/journal.pone.0201198 (PMC6071968; doi:10.1371/journal.pone.0201198)
Supplement: S5 Table — (PDF) [file pone.0201198.s020.pdf]

S5 Table. List of plant IPT^{Pfam} domain genes in Pfam database and results of the hmmsearch.

			IPT^{Pfam} domain in protein		IPT^{Pfam}.hmm matches		hmmsearch result					
			(in `alignment`)		(total 233 AA)		IPT^{Pfam}.hmm		original IPPT^{Pfam}.hmm		IPPT^{Pfam}_N40.hmm	
			Position		Position							
**Taxon**	**Gene Accesion**	**Protein length**	**start**	**end**	**from**	**to**	**E-value**	**score**	**E-value**	**score**	**E-value**	**score**
Musa acuminata	GSMUA_AchrUn_randomT01160	287	13	55	3	45	1.40E-08	25.5	no matches	no matches	6.60E-09	26.6
Oryza barthii	ObaA0A0D3FQI1	433	55	104	4	53	6.60E-10	29.7	no matches	no matches	1.00E-11	35.8
Oryza brachyntha	ObrJ3L312	159	59	100	4	45	1.80E-11	34.8	no matches	no matches	4.10E-09	27.3
Oryza brachyntha	ObrJ3LNW5	139	15	51	2	38	5.40E-09	26.8	no matches	no matches	2.60E-10	31.2
Physcomitrella patens	PpA9SC20	717	191	224	3	36	9.30E-10	29.2	no matches	no matches	7.60E-09	26.4
Solanum lycopersicum	SlK4CKW0	105	14	72	2	61	9.70E-10	29.1	2.40E-09	27.8	1.20E-14	45.5
Solanum lycopersicum	SlK4CKW5	103	14	84	2	73	4.00E-10	30.4	6.30E-08	23.2	5.50E-14	43.3
Solanum lycopersicum	SlK4D6T8	54	1	50	7	56	2.40E-07	21.3	0.0001	12.7	1.10E-10	32.4
Solanum lycopersicum	SlK4D6T9	304	128	185	2	60	2.40E-10	31.1	2.20E-09	27.9	1.00E-14	45.7
Solanum lycopersicum	SlK4D6Y8	96	14	84	2	68	8.90E-09	26	no matches	no matches	3.20E-12	37.5
Solanum lycopersicum	SlK4D6Z0	198	14	127	2	107	3.50E-10	30.6	3.90E-07	20.6	6.30E-12	36.5
Solanum lycopersicum	SlK4D6Z2	113	3	107	9	105	1.00E-07	22.5	4.10E-06	17.2	1.80E-09	28.5
Solanum lycopersicum	SlK4D6Z5	73	1	56	7	62	2.40E-07	21.3	2.30E-06	18	8.40E-08	23
Solanum lycopersicum	SlK4D7J2	55	1	49	7	55	5.60E-09	26.7	1.80E-05	15.1	8.20E-11	32.9
Solanum lycopersicum	SlK4D8Z1	81	13	70	2	60	4.80E-08	23.6	2.10E-06	18.1	5.00E-09	27
Solanum lycopersicum	SlK4D8Z4	91	13	63	2	52	6.50E-09	26.4	9.20E-05	12.8	2.20E-14	44.6
Solanum lycopersicum	SlK4D8Z5	204	13	71	2	61	9.40E-10	29.2	1.90E-09	28.1	4.90E-14	43.5
Solanum lycopersicum	SlK4D8Z7	62	13	54	2	43	2.80E-08	24.4	no matches	no matches	1.60E-10	31.9
Solanum lycopersicum	SlK4D8Z8	139	13	73	2	60	2.80E-09	27.7	2.70E-08	24.4	3.40E-14	44
Solanum lycopersicum	SlK4D8Z9	204	13	71	2	61	9.90E-10	29.1	2.00E-09	28.1	9.50E-15	45.8
Solanum lycopersicum	SlK4D902	197	13	131	2	112	8.70E-09	26	2.20E-07	21.4	1.30E-13	42.1
Solanum lycopersicum	SlK4D903	87	13	70	2	60	1.10E-09	29	1.10E-08	25.7	4.90E-15	46.7
Solanum lycopersicum	SlK4D957	87	13	70	2	60	5.10E-10	30.1	1.30E-08	25.4	5.60E-15	46.5
Solanum lycopersicum	SlK4D958	71	13	55	2	44	2.30E-07	21.4	no matches	no matches	1.20E-07	22.5
Solanum lycopersicum	SlK4D983	204	13	71	2	60	6.40E-09	26.5	1.90E-09	28.2	2.40E-14	44.4
Solanum lycopersicum	SlK4D985	49	1	43	7	49	8.10E-08	22.9	7.90E-05	13	4.00E-11	33.9
Solanum lycopersicum	SlK4D986	204	15	70	4	59	2.70E-09	27.7	2.60E-09	27.7	3.10E-14	44.1
Solanum tuberosum	StM1ANG4	331	176	235	2	61	6.40E-09	26.5	1.40E-06	18.8	9.60E-13	39.2
Solanum tuberosum	StM1DKB5	369	15	82	2	70	2.10E-09	28	6.30E-09	26.4	7.70E-13	39.5
Solanum tuberosum	StM1DKI2a	369	143	207	2	66	1.30E-10	31.9	1.00E-05	15.9	3.00E-12	37.6
Solanum tuberosum	StM1DKI2b	369	238	296	2	60	5.30E-09	26.7	1.10E-07	22.4	7.80E-12	36.2
Solanum tuberosum	StM1DZ85	112	1	82	7	86	8.40E-10	29.3	2.60E-12	37.5	7.70E-12	36.2
