# Supplementary material for: Tangled history of a multigene family: The evolution of ISOPENTENYLTRANSFERASE genes
Source: PLoS One. 2018 Aug 2;13(8):e0201198. doi: 10.1371/journal.pone.0201198 (PMC6071968; doi:10.1371/journal.pone.0201198)
Supplement: S6 Table — (PDF) [file pone.0201198.s021.pdf]

S6 Table. Domains assigned in *ISOPENTENYLTRANSFERASE* genes in model plants and cytokinin biosynthesizing bacteria.

Classification	Organism	Gene	Accession	Pfam family		Pfam clan	
				name	ID	name	ID
α-proteobacteria	*Agrobacterium tumefaciens*	*Tzs*	NP_396682	IPT	PFAM01745	P-loop NTPase	CL0023
α-proteobacteria	*Agrobacterium tumefaciens*	*Ipt*	NP_396529	IPT	PFAM01745	P-loop NTPase	CL0023
Cyanobacteria	*Nostoc sp.* PCC7120	*IPT1*	BAB777444	IPT	PFAM01745	P-loop NTPase	CL0023
Angiosperm	*Arabidopsis thaliana*	*AtIPT1*	NM_105517	IPPT	PFAM01715	P-loop NTPase	CL0023
Angiosperm	*Arabidopsis thaliana*	*AtIPT2*	NM_128335	IPPT	PFAM01715	P-loop NTPase	CL0023
Angiosperm	*Arabidopsis thaliana*	*AtIPT3*	NM_116176	IPPT	PFAM01715	P-loop NTPase	CL0023
Angiosperm	*Arabidopsis thaliana*	*AtIPT4*	NM_118598	IPPT	PFAM01715	P-loop NTPase	CL0023
Angiosperm	*Arabidopsis thaliana*	*AtIPT5*	NM_121909	IPPT	PFAM01715	P-loop NTPase	CL0023
Angiosperm	*Arabidopsis thaliana*	*AtIPT6*	NM_102352	IPPT	PFAM01715	P-loop NTPase	CL0023
Angiosperm	*Arabidopsis thaliana*	*AtIPT7*	NM_113267	IPPT	PFAM01715	P-loop NTPase	CL0023
Angiosperm	*Arabidopsis thaliana*	*AtIPT8*	NM_112803	IPPT	PFAM01715	P-loop NTPase	CL0023
Angiosperm	*Arabidopsis thaliana*	*AtIPT9*	NM_001203415	IPPT	PFAM01715	P-loop NTPase	CL0023
Angiosperm	*Oryza sativa*	*OsIPT1*	AB239797	IPPT	PFAM01715	P-loop NTPase	CL0023
Angiosperm	*Oryza sativa*	*OsIPT2*	AB239798	IPPT	PFAM01715	P-loop NTPase	CL0023
Angiosperm	*Oryza sativa*	*OsIPT3*	AB239799	IPPT	PFAM01715	P-loop NTPase	CL0023
Angiosperm	*Oryza sativa*	*OsIPT4*	AB239800	IPPT	PFAM01715	P-loop NTPase	CL0023
Angiosperm	*Oryza sativa*	*OsIPT5*	AB239801	IPPT	PFAM01715	P-loop NTPase	CL0023
Angiosperm	*Oryza sativa*	*OsIPT6*	AB239807	IPPT	PFAM01715	P-loop NTPase	CL0023
Angiosperm	*Oryza sativa*	*OsIPT7*	AB239804	IPPT	PFAM01715	P-loop NTPase	CL0023
Angiosperm	*Oryza sativa*	*OsIPT8*	AB853903	IPPT	PFAM01715	P-loop NTPase	CL0023
Angiosperm	*Oryza sativa*	*OsIPT9*	AB239806	IPPT	PFAM01715	P-loop NTPase	CL0023
Angiosperm	*Oryza sativa*	*OsIPT10*	AB239807	IPPT	PFAM01715	P-loop NTPase	CL0023
