# Supplementary material for: Tangled history of a multigene family: The evolution of ISOPENTENYLTRANSFERASE genes
Source: PLoS One. 2018 Aug 2;13(8):e0201198. doi: 10.1371/journal.pone.0201198 (PMC6071968; doi:10.1371/journal.pone.0201198)
Supplement: S3 Fig — The tree is rooted on VirEPfam sequences. The αLRT SH-like values (left) and UFBT values (right) are shown on major branches leading to each protein family. Branches with above 70% support values are emphasized by a thick line. (PDF) [file pone.0201198.s003.pdf]

S3 Fig.

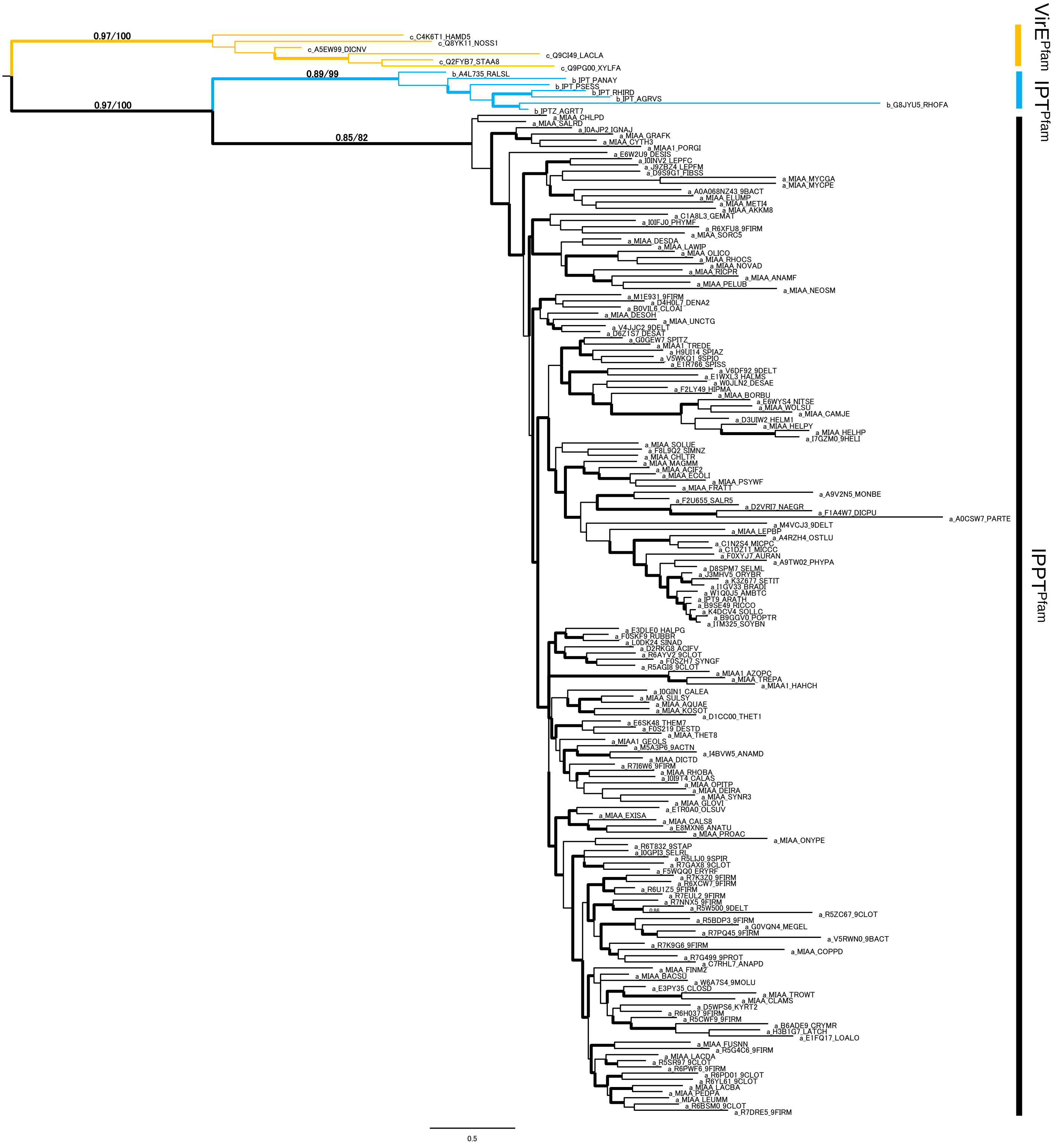

S3 Fig. ML tree based on sequences of IPPT^{Pfam}, IPT^{Pfam}, and pVirE^{Pfam} seed alignments. The tree is rooted on VirE^{Pfam} sequences. The αLRT SH-like values (left) and UFBT values (right) are shown on major branches leading to each protein family. Branches with above 70% support values are emphasized by a thick line.
